# Reassessment of the enigmatic crocodyliform "*Goniopholis*" *paulistanus* Roxo, 1936: Historical approach, systematic, and description by new materials

**DOI:** 10.1371/journal.pone.0199984

**Published:** 2018-08-01

**Authors:** André E. Piacentini Pinheiro, Paulo Victor Luiz Gomes da Costa Pereira, Rafael G. de Souza, Arthur S. Brum, Ricardo T. Lopes, Alessandra S. Machado, Lílian P. Bergqvist, Felipe M. Simbras

**Affiliations:** 1 Faculdade de Formação de Professores (FFP), Universidade do Estado do Rio de Janeiro (UERJ), *Campus* São Gonçalo, Rua Dr. Francisco Portela, Bairro do Patronato, São Gonçalo, Rio de Janeiro, Brazil; 2 Laboratório de Macrofósseis, Departamento de Geologia (DEGEO), Universidade Federal do Rio de Janeiro (UFRJ), Ilha do Fundão, Rio de Janeiro, Brazil; 3 Laboratório de Sistemática e Tafonomia de Vertebrados Fósseis, Setor de Paleovertebrados, Departamento de Geologia e Paleontologia, Museu Nacional, Universidade Federal do Rio de Janeiro (UFRJ), São Cristóvão, Rio de Janeiro, RJ, Brazil; 4 Laboratório de Instrumentação Nuclear—COPPE/UFRJ, Ilha do Fundão, Rio de Janeiro, Brazil; Università di Roma, ITALY

## Abstract

The Crocodyliformes are the most represented vertebrate clade in the Upper Cretaceous sequences of the Bauru Group, Paraná Basin. However, some of the species described have an uncertain taxonomic status and phylogenetic position. For instance, *“Goniopholis” paulistanus* has been assigned as a *nomem dubium*, due to its description being based on scarce material. The “*G”*. *paulistanus* specimens (i.e. teeth and a left tibia) were discovered in two different localities in São Paulo state: Mirandópolis and Valparaíso municipalities; where the upper interval of the Adamantina Formation (Early Maastrichtian of Bauru Group) crops out. Revisiting these specimens, we observed multicrenulated teeth in middle dentary toot- row, a remarkable feature only shared with teleosaurids *Machimosaurus hugii* (Upper Jurassic of Laurasia) and *M*. *rex* (Lower Cretaceous of Tunisia). This apomorphy was also recognized in new material from the Alfredo Marcondes municipality (Presidente Prudente Formation), which are here also referred to “*G*”. *paulistanus*. We recognized the teeth of “*G*.” *paulistanus* as the lectotype, however the tibia cannot be assigned to a species as it was not collected in association with the teeth. We performed a phylogenetic analysis with a data matrix composed of 388 characters and 86 taxa, analyzed in TNT. The strict consensus tree recovered Neosuchia and Ziphosuchia (Notosuchia + Sebecia) within Mesoeucrocodylia. The species *“G” paulistanus* is valid, as a distinct and new genus within Sebecia, in a polytomy with *Barreirosuchus*, *Pepesuchus*, *Itasuchus* and *Peirosaurus*, forming the clade Itasuchidae. *Stolokrosuchus* is the sister taxon to Itasuchidae, the sister group of all other Sebecia (Peirosauridae (Mahajangasuchidae + Sebecidae and taxa *affinis*)). The clades Ziphosuchia, Sebecia and Itasuchidae are here redefined, and we find the last two clades to be more closely related to terrestrial notosuchids than to semiaquatic neosuchians.

## Introduction

The Bauru Group (Lower–Upper Cretaceous) is a famous lithostratigraphic unit because of its fossiliferous content, which mainly comprises vertebrate fossils (e.g. [1–5]). The vertebrate paleobiota of the Bauru Group is remarkable by the diversity of Crocodyliformes, with about 26 described species (i.e. [6–31]). Regarding the fossiliferous context of the Bauru Group, the Adamantina Formation (Turonian–Maastrichtian) is exceptional, mainly by the abundance and its diversity of crocodyliforms. According to Godoy et al. [30], its fossil record provides suggests a complex trophic relationships among the mesoeucrocodyliform paleofauna, probably due to the scarcity or even the absence of other vertebrate groups, such as theropod and ornithischian dinosaurs, which were more common in other areas of Gondwana. Here, we analyzed the record of the entire interval of the Adamantina Formation (and other units depending on the stratigraphic proposal used), in which the temporal range comprises the Turonian-Maastrichtian age, and could exhibit more than 25Ma. The lack of an appropriated biostratigraphic correlation of the clades or assemblage zones implies in innacurate results in the trophic analysis performed by Godoy et al [30]. Besides, recent works [32] suggest a potential bias on the vertebrate fossil record from Bauru Group, with the mesoeucrocodylians providing a more complete specimens due its life habit (near river plains and streams) and burial behavior.

Additionally, some of these species of the Bauru Group are of doubtful validity, such as *Brasileosaurus pachecoi* (e.g. [33]), *Caipirasuchus montealtensis* (e.g. [34; 31]), and *Uberabasuchus terrificus* (e.g. [35]). One of the most debated species is *Goniopholis paulistanus*, especially concerning the presence of the genus *Goniopholis* in Gondwana [10, 33, 36].

*Goniopholis* is a semiaquatic goniopholidid neosuchians genus from the Upper Jurassic–Lower Cretaceous of Europe (e.g. [36, 37]). However, three species have already been assigned to this genus in Brazil. The first was *Sarcosuchus hartti*, from the Recôncavo Basin (Salvador Formation, Lower Cretaceous of Bahia), which was first considered as a member of *Crocodylus*, and subsequently, was assigned to *Goniopholis* by Mawson & Woodward in 1907 [38]), and lately to the current genus (see [39]). The second species was *Thoracosaurus bahiensis*, also from the Salvador Formation, Recôncavo Basin, which was previously grouped within *Goniopholis* genus [38], but lately it was moved back to *Thoracosaurus* [40], but now considered to be a *nomem dubium* [41]. The last species is *Goniopholis paulistanus*, from the Adamantina Formation, Upper Cretaceous of the Bauru Group, which was also considered a *nomem dubium* by some authors (e.g. [36, 42]).

In the present work we reevaluate the taxonomic status of *Goniopholis paulistanus* with description of new specimens from the uppermost depositional sequence of the Presidente Prudente Formation (Late Campanian–Early Maastrichtian), Bauru Group. The validity of this species is supported by the presence of diagnosable features, and a new generic combination is proposed here. We provide a new stratigraphic interpretation for the levels of the Crocodyliformes occurrences of the Bauru Group, suggesting Assemblage Zones of crocodyliforms based on sequence stratigraphic analysis. The newly described specimens also reveals another tooth morphology to the already diverse dentition exhibited by the Bauru’s mesoeucrocodylians. The systematic affinities is supported by a phylogenetic analysis herein, suggesting a complex evolutionary relationship and niche occupations between the “notosuchians” (*sensu* [31]) during Cretaceous of Gondwana. We also redefine some inclusive taxa as Ziphosuchia, Sebecia, and Itasuchidae.

### Historical review of platyrostral mesoeucrocodylians from the Bauru Group

Price [43] in the first review of the mesoeucrocodylian fossils of the non-marine Cretaceous formations of the Bauru Group, pointed out the wide distribution of this taxon in these sedimentary strata. For long, the few Late Cretaceous mesoeucrocodylians remain from the Bauru Group that have moderate to long snouts, and a platyrostral shape (according to [44]) with conical monocuspided and tooth with circular cross-section (e.g. *Itasuchus jesuinoi*, *Pepesuchus deiseae*, *Barreirosuchus franciscoi*) have been considered as semi-aquatic morphotypes, despite their controversial phylogenetic systematic. Many authors, based on cladistic methods (e.g. [36]) or not (e.g. [7, 10, 28, 43, 45–48]), suggested that those species are related to the neosuchian lineage. While other authors suggest that some species (e.g. *Pepesuchus deiseae*) have affinities with notosuchians and peirosauromorphs (e.g. [24]). These relative scarce morphotypes are markedly distinct when compared with the prolific notosuchians species from the same lithostratigraphic units (e.g. peirosauromorphs, sphagesaurids, baurusuchids), many of which have brevirostrine and oreinirostral snouts and heterodont dentition (e.g. [31, 49, 50]).

The first identification of crocodyliforms in the Bauru Group was made by Von Ihering [45]. He also made the first vertebrate fossil collection from these strata, recovered from a well made for water supply in São José do Rio Preto municipality (Northwestern São Paulo State) in 1909. After analyzing the external morphology of two isolated teeth from this material, von Ihering recognized several similarities with the teeth of *Machimosaurus* and *Goniopholis* [45]. *Machimosaurus* is a marine teleosaurid genus (Thalattosuchia) from the Upper Jurassic (Oxfordian to Late Kimmeridgian–Early Tithonian) of central Europe (i.e. *Machimosaurus* sp., *M*. *buffetauti*, *M*. *hugii* and *M*. *mosae*), and from Upper Jurassic (Oxfordian-Kimmeridgian) to Lower Cretaceous (Hauterivian) of north and east Africa (i.e. *M*. *nowackianus* and *M*. *rex*) (e.g. [51, 52, 53]). *Goniopholis* is a semi-aquatic goniopholidid genus (Neosuchia) known from coastal marine and brackish ecosystems during the Upper Jurassic (Kimmeridgian) to Lower Cretacesous (Berriasian) of continental Europe and England, and was recently limited to just the species *G*. *simus*, *G*. *baryglyphaeus* and *G*. *kiplingi* by [36] (e.g. [36, 37, 54]).

Due to the uncertainty as to which genus to assign the teeth to (i.e. *Machimosaurus* or *Goniopholis*), von Ihering [45] considered them as belonging to Goniopholididae. Later, von Huene (1931) revisiting the brazilian teeth materials, corroborated the hypothesis that one of them belong to *Machimosaurus*, specifically to *M*. *hugii* von Meyer, 1837, from the Upper Jurassic (Upper Kimmeridgian—Lower Tithonian) of Europe (Portugal, Spain, and Switzerland [51]). However, Price [43] warned that in the brief description of the tooth given by von Ihering [45], which is very similar for both teeth, exhibiting as the unique distinction the acute apex, which could be an artifact provided by the Tooth wear.

A second collection of vertebrate fossils from Bauru Group was assembled in 1911 and described by J. Pacheco in 1913 [55], who dubiously considered a tooth collected in the Municipality of Colina (São Paulo State) as belonging to *Goniopholis*. Von Huene [6] considered it to belong to a long-snouted mesoeucrocodylian, but he did not assign it to *Goniopholis*. Price [43] doubted the “*Goniopholis* hypothesis” for referred tooth, but unfortunately, it was lost.

**Fig 1:**
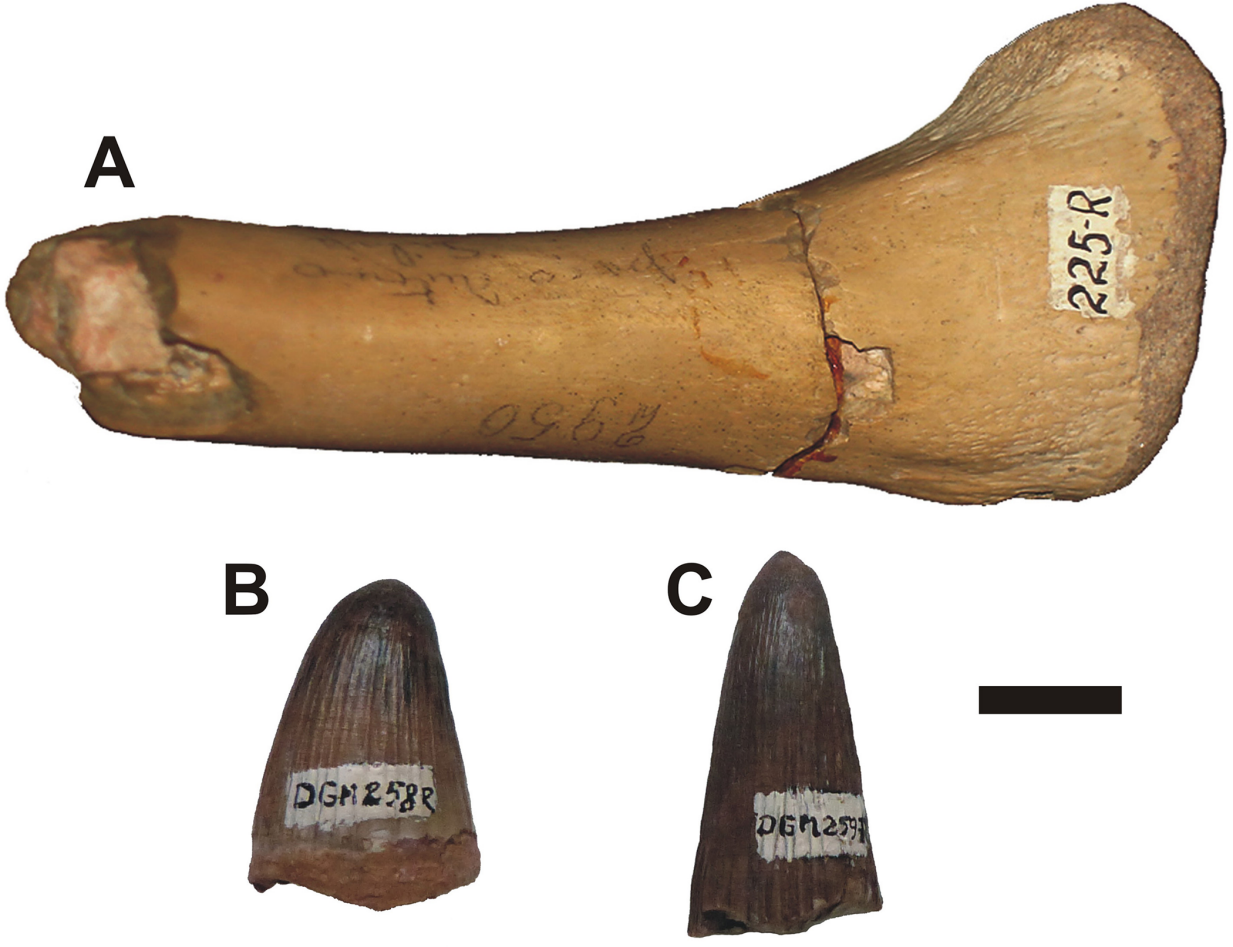
"*Goniopholis*" *paulistanus*, original material described by Roxo [7]. A- right proximal fragment of a tibia (DGM 225-R); B- (DGM 258-R) and C- (DGM 259-R) isolated teeth. Scale bar = 10 mm.

The most important record of *Goniopholis*, and the one used to describe its only Brazilian species is cited in the work of Roxo [7]. This author assigned two teeth (DGM 258-R and DGM 259-R) to this genus, associated with “one bone”, and to a supposed amphicoelic caudal vertebrae, currently missing and not figured in his work [7], and the epiphyseal fragment of a right tibia (DGM 225-R), without diagnostic features (Fig 1). According Roxo [7] and Campos and Castro [56], the teeth were found by Mr. Alberto F. L. Wanderley in 1935, in a road section known as mark 103.7 km of the Northwest Railway, localized near Amandaba city, in the Mirandópolis municipality. Whereas Mr. Heitor Serapião collected the tibia at 20 m of depth in a well for supply water, beside the same railway in the Valparaíso station of the homonymous municipality. The tibia site is around 40 km from the teeth site. Due to the tooth morphology (i.e., acute, robust, circular to subcircular cross-section, having high relief apicobasal low ridges on the labio-lingual crown surfaces, and smooth or crenulated mesial and distal carinae) and dental comparisons, the specimens DGM 258-R and DGM 259-R were originally assigned to *Goniopholis* [7]. Roxo [7] considered this specimen to be closely related to “*G*.” *affinis* (Lower Cretaceous, Aptian, of North America), and described a new species, *Goniopholis paulistanus*, for the material. Also, Roxo [7] considered “von Ihering’s teeth”, “Pacheco’s tooth” and the fragmentary materials (donated by Prof. G.B. Milward to SGB, MCT Rio de Janeiro in 1917) from the disabled Guajussara section between Guarucaia (Actually Presidente Bernardes municipality) and Santo Anastácio railway stations of “*Sorocabana Railway*” (rail network in Mesoregion of Presidente Prudente, west of São Paulo state (*vide* [7, 46, 56]), as being referrable to *G*. *paulistanus*.

After the erection of *G*. *paulistanus* and the scientific impact of the putative presence of a setentrional group, until them, for South America, isolated teeth with the previoysly described morphotype recovered from the Bauru Group were reluctantly assigned to Goniopholididae in some works (e.g. [57, 58]). Suárez, in 1973 [59] assigned to Goniopholididae some cranial material (maxillary fragments) with associated and isolated teeth, collected from fine sandstones of Bauru Group in Southwest São Paulo State. Bertini described another record of Goniopholididae in 1993, comprising twenty-three isolated teeth collected in rocks of the Adamantina Formation (URC R.1–R.14 and URC R.16–R.24) from the Santo Anastácio quarry, to Goniopholididae indet. [60, 61].

Most published studies recover Goniopholididae as a monophyletic group within Neosuchia (e.g. [36, 62–66]). Goniopholidids were semi-aquatic forms with a covergently similar body-plan to crown group Crocodylia, and were the first crocodyliforms to evolvet platyrostrine snouts (although some species, such as *Calsoyasuchus vallicepsi*, had tubular snouts, however the ancestral snout morphotype for the group remains unkown) in freshwater environments [36, 62]. However, reliable data indicates a group restricted to the Early Jurassic (Sinemurian–Pliensbachian) to Upper Cretaceous (Campanian) in the Laurasian landmasses (i.e. USA, Portugal, Spain, England, France, Belgium, Germany, Kirghizistan, China, Mongolia and Thailand, e.g. [36, 54, 66–68]).

According to Andrade *et al*. [36], goniopholidids might have been occurred in Gondwana, as reported by Sereno [69] based on an undescribed “*Sunosuchus*"-like taxon from North Africa. Otherwise, there is no indisputable evidence that goniopholidids were present in South America. Worldwide reports of goniopholidids have been based mostly on teeth or fragmentary material, and a wide sample of teeth and osteoderms can be found in most palaeontological collections in Europe, North America, Brazil and Asia [36]. However, Andrade *et al*. [36] warned that despite tooth crown morphology beinga useful tool for gross recognition of morphotypes, taxonomic assignment or species definition must also be supported by a phylogenetic framework, or by other meaningful associated remains. Andrade *et* al. [36] in their revision of *Goniopholis*, regarded the scarcity of and non-diagnostic characters for *Goniopholis paulistanus*, and proposed it as a *nomen dubium* [36].

Reinforced by the absence of diagnostic goniopholidid fossils, some authors have been more skeptical about their presence in South America, and have referred similar teeth to the controversial group Buffetaut erected Trematochampsidae (e.g. [48]) in 1974, as a monospecific group, to hold the remains of *Trematochampsa taqueti* [70], a fragmentary and enigmatic mesoeucrocodylian from the Upper Cretaceous of Niger, Africa. Despite the presence of a small antorbital fenestra, festooned maxilla (a classic neosuchian synapomorphy of Benton and Clark [71]) and double craniomandibular articulation (quadrate and quadratojugal in skull *versus* articular and surangular in the mandible), Trematochampsidae diagnosis was based on tooth number and dental size variation [70–75]. After *T*. *taqueti*, some even fragmented taxa from Gondwana (except *Ischyrochampsa meridionalis*, from Upper Cretaceous of France [73]) have been included in this clade (i.e. *Amargasuchus minor* from Early Cretaceous of Argentina [71]; *Miadanosuchus oblita* from the Upper Cretaceous of Madagascar [74, 75]; and the first remains of *Hamadasuchus rebouli*, from the Early Cretaceous of Morocco [76]). In addition, many presumed semiaquatic morphotypes from the Cretaceous of Brazil with uncertain affinities were referred as trematochampsids (i.e. *Itasuchus jesuinoi* [47], *Caririsuchus camposi* [77] and *Barreirosuchus franciscoi* [28]).

Before crocodylomorph systematics used a phylogenetic approach, Trematochampsidae was frequently treated as a member the “Suborder Mesosuchia” (e.g. [47, 72–74]). However, after Benton and Clark [71] and after the establishment of the systematic phylogenetic methods, Trematochampsidae and its related taxa started to be considered within the newly proposed Neosuchia clade, which in parts replaces the taxon Mesosuchia, even without results that properly supports these statements (e.g. [28, 78]). As pointed by Larsson and Sues [35] and Turner and Buckley [63], the type material of *T*. *taqueti* comprises multiple individuals that, despite being collected from the same locality, makes the validity of the species questionable. The fragmentary nature of many trematochampsid species, and the issues surrounding the *T*. *taqueti* holotype, have led many authors to doubt and reject Trematochampsidae as a natural group (e.g. [35, 63, 79–82]).

Price [10] noted some differences between the tooth morphology of *Itasuchus jesuinoi* (a more complete material than the one of the species *G*. *paulistanus* [i.e. maxillary posterior fragment with four alveoli; left jugal, quadratojugal and quadrate; dentaries; articulars and some postcranial elements]) and the genus *Goniopholis*. Despiste this Price [10], in the absecense of better anatomical information, assigned *I*. *Jesuinoi* to Goniopholididae. *Caririsuchus camposi*, a semiaquatic species from the Lower Cretaceous of the Araripe Basin (Northeast Brazil) and originally designed as belonging to an uncertain family (see [77]), was referred to the genus *Itasuchus* by Buffetaut (47) through photography analysis. Therefore, both species, *I*. *jesuinoi* and *C*. *camposi*, are referred by him (op. cit.) to Trematochampsidae family.

*Pepesuchus deiseae* is a mesorostral (“normal” *sensu* Busbey III [44]), tubular platyrostral form from the Presidente Prudente Formation. Based on some cranial characters (e.g. five premaxillary teeth, the two anterior premaxillary alveoli nearly confluent), this taxon was originally assigned as a peirosaurid [24].

The last platyrostral mesoeucrocodylian described for Bauru Group (Adamantina Formation) is the large *Barreirosuchus franciscoi*, with holotype body size estimated to be 4 meters long [28]. Despite the well-preserved braincase, the mid-anterior region of the snout is lost, but even so, the preorbital region remains exhibits a clear broad platyrostral form. The taxon *Barreirosuchus franciscoi*, which is distinct from any taxa found in the Bauru Group, was originally considered a neosuchian belonging to “Trematochampsidae” family, however without any phylogenetic analysis [28].

## Geological setting

### Historical background

**Fig 2:**
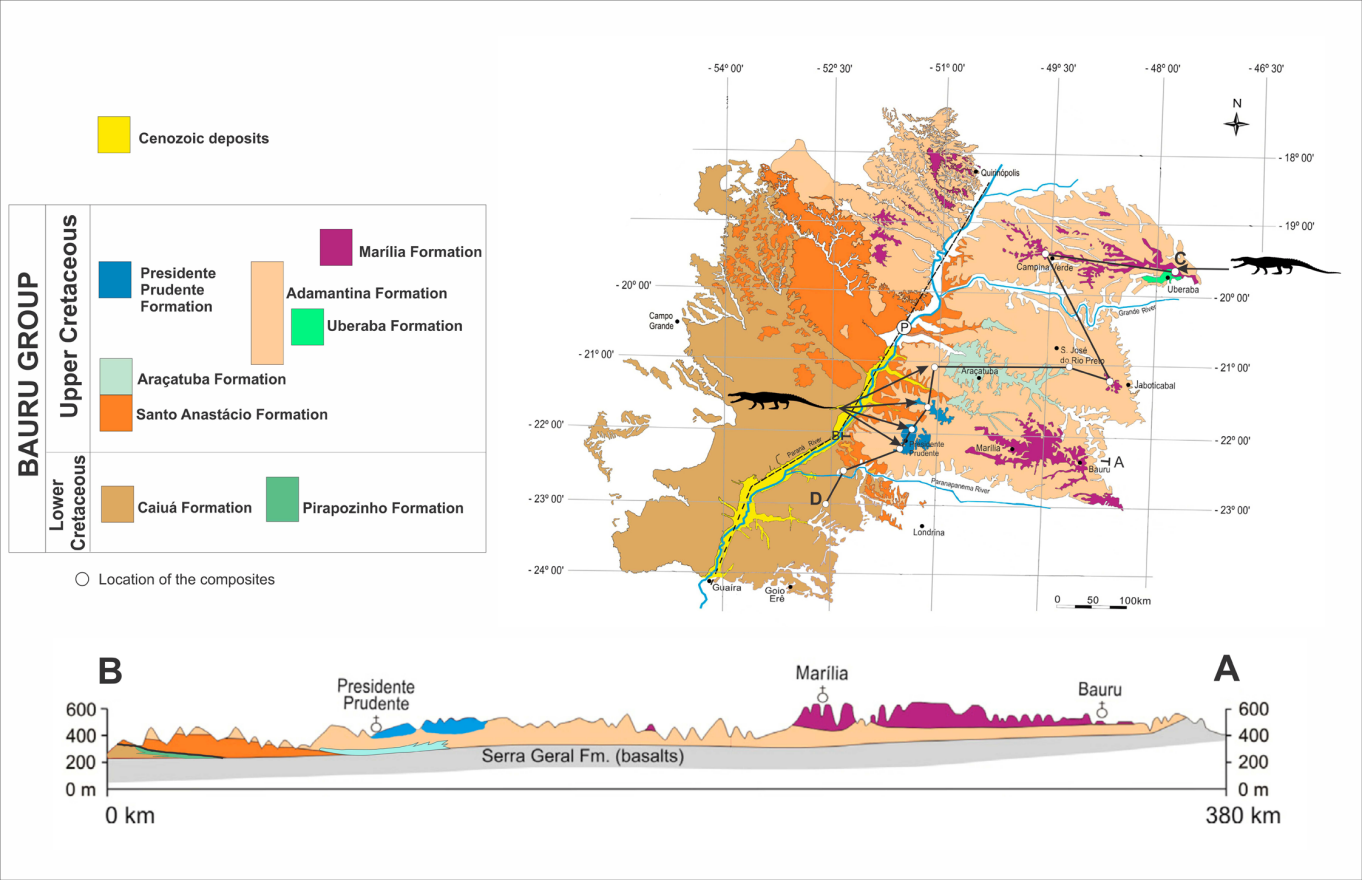
Geological map of the Bauru Group, Paraná Basin (modified from [104]). The outcrops that yielded Itasuchidae specimens is located with the crocodile outlines. A-B and C-D are regional geological sections.

The Bauru Group is a continental unit dated from the Aptian to the Maastrichtian age (*sensu* [83, 84]), Early-Late Cretaceous of the Paraná Basin. This unit crops out in an expressive area of approximately 370.000 square kilometers, and covers part of the current Brazilian states of Paraná, São Paulo, Mato Grosso do Sul, Mato Grosso, Goiás and Minas Gerais, as well as parts of Paraguay (Fig 2). It recovers the basalts of the Serra Geral Group (*sensu* [85]), and in a sequence stratigraphic approach is denominated Bauru Supersequence, one of the six second-order depositional sequences proposed by Milani et al. [86] that filled the Paraná Basin Two distinct intervals compose the Bauru Group: a lower one formed by eolian to fluvial—eolian sandstones of the Aptian, and an upper one formed by alluvial and fluvial conglomerates, sandstones and mudstones, with subordinated lacustrine mudstones dated in the Late Cretaceous (*sensu* [83, 84]).

**Fig 3:**
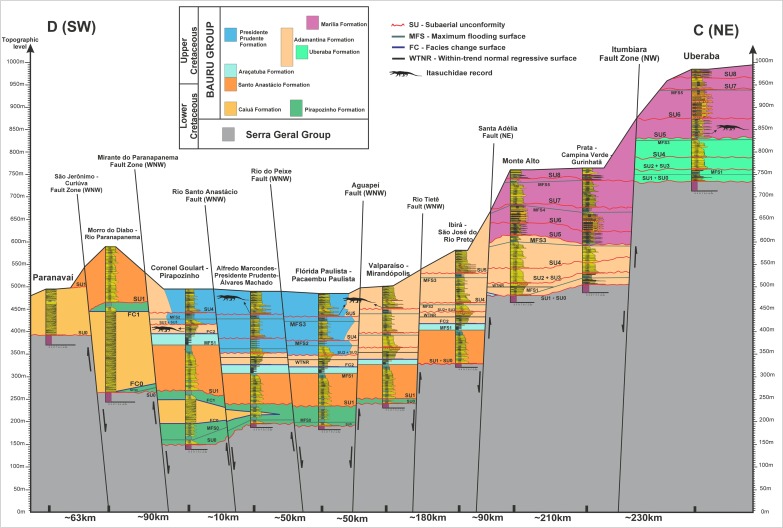
Cross-section of correlation of vertical profile composites of the Bauru Group. Showing the lithostratigraphic framework at Formation level of the Bauru Group.

**Fig 4:**
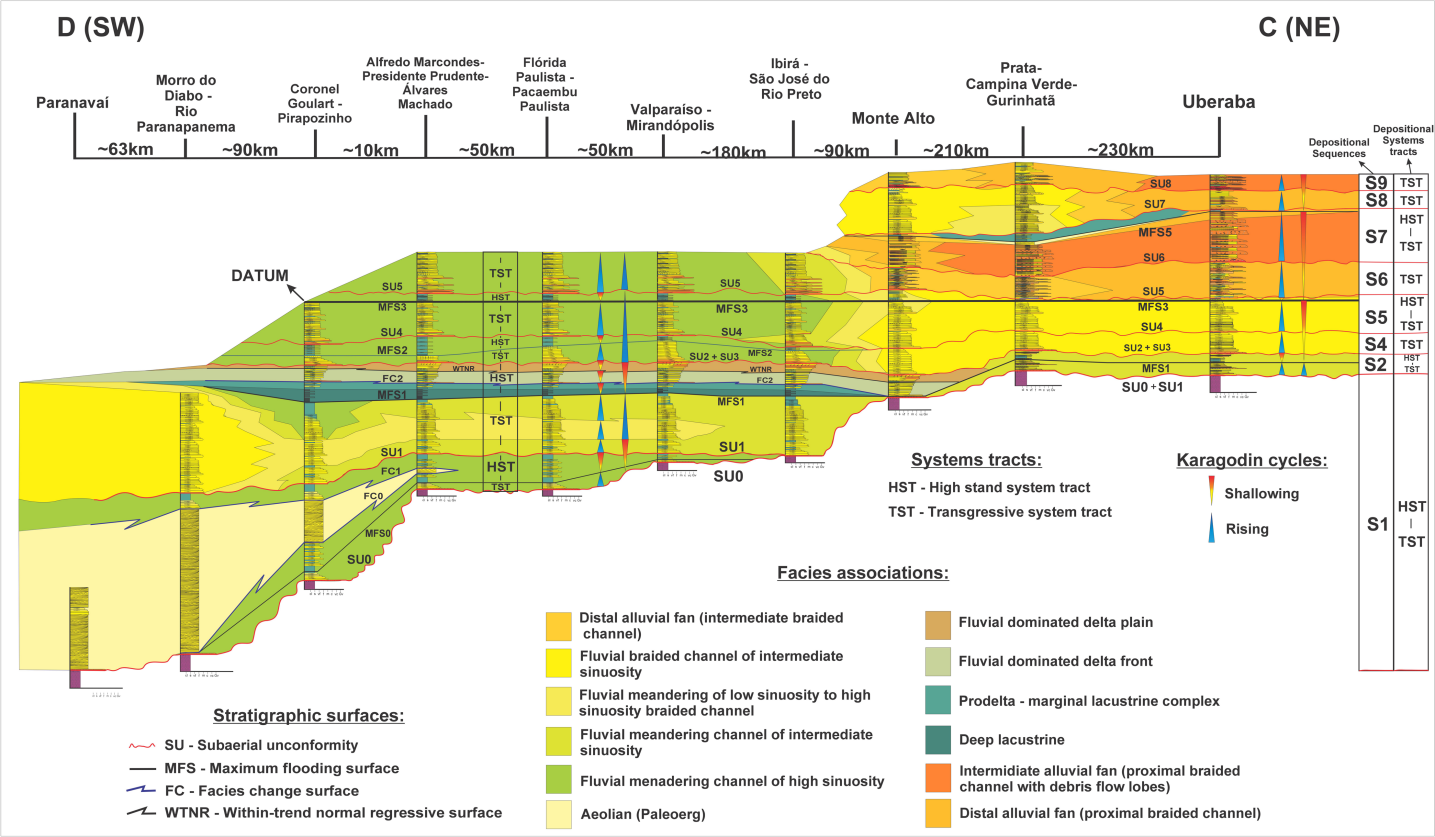
Cross-section of correlation of vertical profile composites of the Bauru Group. Showing the sequence stratigraphic framework at facies association level of the Bauru Group.

**Fig 5:**
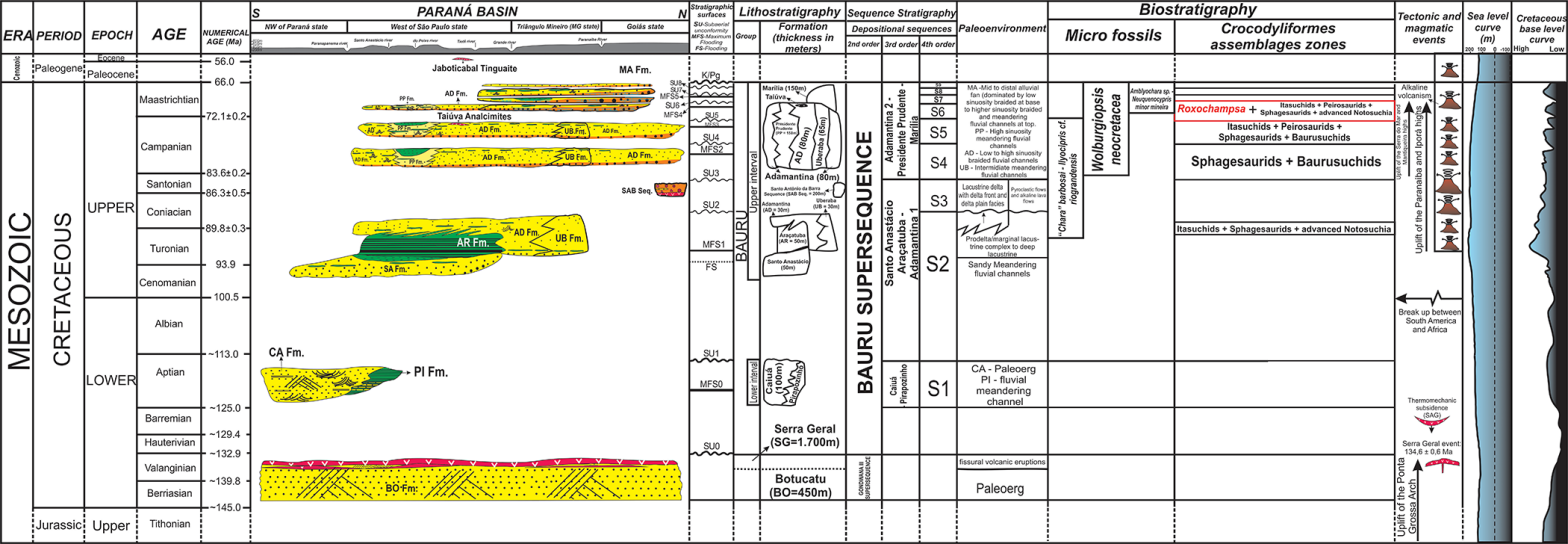
New stratigraphic chart of cretaceous of the Paraná Basin. Proposition of a revised chrono-lithostratigraphy of the Bauru Group.

Some stratigraphic proposals tried to organize the Bauru rocks in different sets of formations (see discussion in [83, 84, 87–105]). Here, we follow the main lithostratigraphic proposal of Soares et al. [84], where the Bauru Group is formed by Caiuá, Santo Anastácio, Adamantina and Marília formations, together with four more units derived from recent stratigraphic refinement derived from the first integrative proposal. These four formations are: Uberaba [106]; Araçatuba [107]; Presidente Prudente [87]; Pirapozinho [100]. Therefore, these eight formations ofthe Bauru Group have the following ages based on the integration of all studies done so far here analyzed: Caiuá (Aptian), Pirapozinho (Aptian), Santo Anastácio (Cenomanian), Araçatuba (Turonian), Adamantina (Turonian–Early Maastrichtian), Uberaba (Coniacian–Campanian), Presidente Prudente (Campanian–Early Maastrichtian), and Marília (Maastrichtian) (Figs 2–5). Our stratigraphic framework is based on [84, 87, 100, 106], moreover, we present a new chart for the Cretaceous of the Paraná Basin (Fig 5), including the crocodilian biostratigraphic data for the Bauru Group restricted to the sequences of the Bauru Group, establishing crocodyliforms Assemblages Zones. This chart was based on the sequence stratigraphic framework (Fig 4), which was compared with a regional lithostratigraphic analysis (Fig 3).

### Stratigraphic analysis

The stratigraphic analysis consists of the correlation of the depositional sequences and stratigraphic surfaces found in type composite vertical profiles where longirostrine crocodyliforms species are recorded (Uberaba, Monte Alto, Valparaíso-Mirandópolis, Alfredo Marcondes, and Pirapozinho areas). The locality and stratigraphic levels were interpreted based on the subsuperficie published data [101–104] and based on outcrop data acquired from papers post-2008 [104–106]. According to Roxo [7], and Campos and Castro [56], the holotype of “*Goniopholis” paulistanus*, which is composed by two teeth (DGM-258-R and DGM-259-R) and a broken tibia (DGM-225-R), were collected in two different localities, both in the western São Paulo state. The teeth were discovered in a section of the Northwest Railway, localized near Amandaba city, in Mirandópolis municipality, whereas the tibia was collected in a well for supply water, beside the same railway of the teeth, in the Valparaíso station of the homonymous municipality. These specimens were recovered from fine sandstones facies of lower sinuosity meandering channels at the heights around 455m (teeth) and 433m (tibia), top of the Adamantina Formation (Late Campanian) (Figs 3 and 4).

The Adamantina Formation is primarily composed of fine to mid sandstones, with rare conglomerates and siltstones facies of braided to meandering fluvial paleoenvironment (*sensu* [84, 87]), and is the most prolific unit for fossils crocodilyforms. Most of the crocodyliform discoveries in the Adamantina Formation were made in different levels of fluvial sandstones facies, normally not correlated. Therefore, authors usually assigned a large temporal range of Turonian-Santonian age, as proposed by Dias-Brito et al. [83], an interval of more than 10 Ma according to Cohen et al. [108], for the single horizon that provided the holotype specimen (e.g. [109]). This chronostratigraphic interpretation is influenced by the absence of tentative positioning of the fossil in the unit interval (for example lower, middle, or upper interval). Also, the absence of a more refined stratigraphic analyses or high-resolution stratigraphy approach for the Bauru Group and its formations, makes it difficult to temporally calibrate paleontological data.

However, we can be assured that their lower record is at the base of the Adamantina Formation, which is dominated by sandstones and mudstones of distributary channels of lacustrine delta front in Pirapozinho and in Marília regions (e.g. [14, 17, 24]). The crocodylomorphs were also discovered in the middle interval of the Adamantina Formation, also dominated by braided sandstones facies, as in the regions of General Salgado (e.g. [13, 20, 21, 29, 30]) and in Campina Verde county in Minas Gerais state [26]. Additionally, there were discoveries in the uppermost meandering levels of Monte Alto [16, 27, 28], Catanduva, Ibirá and São José do Rio Preto regions [18, 27].

The new two specimens studied here were collected in the rural area of the Alfredo Marcondes municipality, southwestern São Paulo state, from the AM1 site, which provided many others dinosaur materials [110–112] (Fig 2). The larger specimen was found in the second conglomeratic level, and the smaller one was collected on the surface of recent alluvial to colluvial deposits, between the second and the first levels of conglomerate, indicating its provenience from the layer above (Fig 4). According to Simbras [113] and Azevedo et al. [112], these conglomerate facies filled gravel bars of the base of high sinuosity fluvial meandering channels of the Presidente Prudente Formation. In the AM1 site outcrops the upper sequence of the Presidente Prudente Formation, its boundary is a subaerial unconformity marked by the conglomerate 1 in the vertical profile (Fig 4). This unconformity is correlated with the upper unconformity of the Adamantina Formation in Ibirá, and São José do Rio Preto regions, as well as with the subaerial unconformity of the contact between the Adamantina and Marília formations in Monte Alto and Catanduva municipalities. Based on the Maastrichtian age for the Marília Formation, we consider this subaerial unconformity to be the boundary between the Campanian and Maastrichtian in the Bauru Group. Therefore, the new specimens of Alfredo Marcondes municipalities are dated as late Campanian to early Maastrichtian in age, regarding the temporal lapse involved in the unconformity.

## Materials and methods

### Anatomical nomenclature and abbreviations

We employed the Romerian terminology and the directional terms instead of veterinarian alternatives. ‘‘Anterior” and ‘‘posterior,” for example, are used as directional terms rather than the veterinarian alternatives ‘‘rostral” or ‘‘cranial” and ‘‘caudal” (except for dentition anatomical orientation [114]). For the anatomical description of skull and dentition, we followed [50, 114–118].

### Anatomical abbreviations

**ab**- alveolar bone; **ad7**- d7 alveolus; **ad8**- d8 alveolus; **ag18**,**19**-, alveolar groove for d8 and d9; **d**- dentary; **d1**-**d26**- respective dentary teeth; **dg**- dentary lateral groove; **dr**- dentin ridge; **ds**- dentary symphysis; **Mkc** and ***mc***- Meckel’s channel; **MS**- mandibular symphysis; **os**- occlusal scar; **pc**- pulp cavity; **po**- primary osteon; **rd**- replacement dentary teeth; **sp**- splenial; **sps**- splenial symphysis; **tc**- tooth crown; **tr**- tooth root; **vc**- vascular channels.

### Institutional acronyms

**COPPE-UFRJ—**Instituto Alberto Luiz Coimbra de Pós-graduação e Pesquisa de Engenharia of the Universidade Federal do Rio de Janeiro, RJ, Brazil; **CPRM**- Companhia de Pesquisas e Recursos Minerais, Rio de Janeiro, RJ, Brazil; **DEGEO**- Departamento de Geologia of UFRJ *Campus* Fundão, RJ, Brazil; **DGM**- Divisão de Geologia e Mineralogia, extint departament of Departamento Nacional de Produção Mineral (DNPM), RJ, Brazil; **DGP**- Departamento de Geologia e Paleontologia of Museu Nacional / UFRJ, Rio de Janeiro, RJ, Brazil; **FFP**- Faculdade de Formação de Professores of UERJ *Campus* São Gonçalo, RJ, Brazil; **IPB**- Institut für Palaontologie der Universitat Bonn, Germany; **LIN**- Laboratório de Instrumentação Nuclear (Nuclear Instrumentation Laboratory) of COPPE-UFRJ; **MCT**- Museu de Ciências da Terra, Rio de Janeiro, RJ, Brazil; **MN**- Museu Nacional of UFRJ, RJ, Brazil; **MPMA**- Museu de Paleontologia de Monte Alto, SP, Brazil; **MUGEO**- Museu Geológico Valdemar Lefèvre, São Paulo, SP, Brazil; **PVL**- Istituto Miguel Lillo, San Miguel de Tucumán, Tucumán province, Argentina; **SGB**- Serviço Geológico do Brasil (Brazilian Geological Survey), Rio de Janeiro, Brazil; **SMNS**- Staatliches Museum für Naturkunde Stuttgart, Germany; **UERJ**- Universidade do Estado do Rio de Janeiro, RJ, Brazil; **UFRJ**- Universidade Federal do Rio de Janeiro, RJ, Brazil; **URC**- Geology department of Universidade Estadual Paulista, *Campus* Rio Claro, SP, Brazil.

### Nomenclatural acts

The electronic edition of this article conforms to the requirements of the amended International Code of Zoological Nomenclature, and hence the new names contained herein are available under that Code from the electronic edition of this article. This published work and the nomenclatural acts it contains have been registered in ZooBank, the online registration system for the ICZN. The ZooBank LSIDs (Life Science Identifiers) can be resolved and the associated information viewed through any standard web browser by appending the LSID to the prefix "http://zoobank.org/". The LSID for this publication is: urn:lsid:zoobank.org:pub:2F405960-34F6-449F-9B29-6E157E51C6C6. The electronic edition of this work was published in a journal with an ISSN, and has been archived and is available from the following digital repositories: PubMed Central and LOCKSS.

## Methods

### Tree search and support

The phylogenetic analysis used herein is derived from Barrios *et al*. [119], but which originally corresponds to Pol et al. [31, 120] dataset. This datset originally had 109 taxa and 412 characters [119]. Herein we revise the dataset, for both character writing/proposition and codification, resulting in a new database with 25 characters excluded. The following criteria for character exclusion, based on Poe and Wiens [121], also see [122], was applied to the present work: 1) comparative (High variation, Substantial missing data and Continuous variation); 2) logical (Character correlation and Mixed character statements); and, 3) operational (Ambiguous or imprecise descriptions, Incomplete quantitative-relative and character statements). The excluded characters matched at least one of these criteria. Some characters were rewritten to give clearer comprehension. Also, new statements were proposed for some characters to include variations not originally contemplated. Finally, the original character 103 was here divided in two (characters 94 and 388). Regarding the taxa, 24 were removed from initial analysis (i.e. Kayenta Form, *Edentosuchus*, *Fruitachampsa*, *Shantungosuchus*, *Sokotosuchus*, *Dyrosaurus*, *Rhabdognathus*, *Alligatorium*, Glen Rose Form, *Bernissartia*, *Asiatosuchus germanicus*, *Leidyosuchus canadensis*, *Borealosuchus*, *Argochampsa*, *Eothoracosaurus mississippiensis*, *Araripesuchus tsangatsangana*, *Araripesuchus buitreraensis*, *Araripesuchus patagonicus*, *Pakasuchus*, *Chimaerasuchus*, *Morrinhosuchus*, MSZP PV 139, *Cynodontosuchus* and Lumbrera Form) for the absence of data for them and their irrelevance to the topological relationship of “*Goniopholis*” *paulistanus* including *Peirosaurus tormini* due to its fragmentary nature and possible synonym with *Uberabasuchus terrificus* (e.g. [24, 35, 123]). For outgroup polarization we used the species *Gracilisuchus stipanicicorum*. The 388 characters and 85 taxa, together with our modifications, are listed in the supplementary material.

The data matrix was constructed using NDE version 0.5.0 [124] and the program Mesquite version 3.03 [125] was employed to convert NDE NEXUS file to a new TNT compatible file. The data matrix was analyzed on TNT version 1.1 [126]. All analyses performed in the present work followed this protocol: traditional search; random seed = 0; 3.000 replications; swapping algorithm was TBR with 15 trees saved per replicate; replace existing trees; and, collapse trees after search. For the polarization of characters, the principle of outgroup was implied, with *Gracilisuchus* at the root of the tree. The protocol “stats.run” was used to calculate the ensemble consistency and retention indices. After analyses, the strict consensus of all minimum-length trees was produced.

### Micro CT-scan imaging

The micro-Computed Tomography-scan (micro CT-scan) of the UFRJ-DG 451-R was performed at the SkyScan/Bruker 1173 system in the Laboratório de Instrumentação Nuclear of the Universidade Federal do Rio de Janeiro (LIN-UFRJ). The parameters set up for the sample scanning are listed in Table 1.

**Table 1:** Set up of the micro CT-scan scanning conditions.

Parameters	Values
**Voltage (kV)**	130
**Current (μA)**	61
**Matrix (pixels)**	2240x2240
**Exposition time (ms)**	1000
**Filter**	0.5 mm Cu
**Pixel Size (μm)**	35.62

The micro-tomographic analysis resulted in 3031 BMP images, showing coronal sections of the fragmented hemimandible UFRJ-DG 451-R. These images were compiled to generate sagittal and transversal sections by the software Skyscan DataViewer version 1.5.1.2 [127]. The sagittal sections were converted and reduced to 640 PNG images, which were segmented and reconstructed in 3D models by SPIERS version 2.2 [128], following the protocol proposed by Abel et al. [129]. The measurements of the evident deposition lines of dentine from micro CT-scan images were performed with Image J software [130]. We selected the sagittal and coronal slices that intercepted the mid region of each tooth. We calculated via ImageJ the ratio between the length of the dentine line to the pulp cavity and the thickness of the tooth (Rdt).

## Results

### Systematic paleontology

MESOEUCROCODYLIA Whetstone and Whybrow, 1983

ZIPHOSUCHIA modified from Ortega, Gasparini, Buscalioni and Calvo, 2000

SEBECIA modified from Larsson and Sues, 2007

ITASUCHIDAE modified from Carvalho, Ribeiro and Ávilla, 2004

**Modified phylogenetic definition**: all species closer to *Itasuchus jesuinoi* than to *Barreirosuchus franciscoi*, *Montealtosuchus arrudacamposi*, *Mahajangasuchus insignis* and *Sebecus icaeorhinus* (stem-based).

*Roxochampsa* **gen**. **nov**.

urn:lsid:zoobank.org:act:5A667044-7574-48CF-A547-5B5849436E85

**Genus etymology**: *Roxo* in honor to an important vertebrate paleontologist from DGM, Mathias de Oliveria Roxo, and the Greek suffix ***Xαμψαι*** *(Champsai* Latinized as ‘‘*champsa*”) meaning crocodile.

**Type species**: *Roxochampsa paulistanus* Roxo, 1936.

**Diagnosis for genus**: same as for the only known species.

*Roxochampsa paulistanus* (Roxo, 1936) **comb**. **nov**.

**Basionym**: *Goniopholis paulistanus* Roxo, 1936.

**Lectotype**: DGM 259-R, an isolated and acute tooth apex.

**Paralectotype:** DGM 258-R, an isolated and acute tooth apex (wider but lower than DGM 259-R).

**Lectotype-paralectotype locality**, **lithology and horizon**: Northwest of São Paulo state, between Três Lagoas (Jupiá old designation) and Araçatuba municipality, collected in a railway section of Noroeste do Brazil Railway [7, 56]. Top of the Adamantina Formation, Upper Cretaceous (Early Maastrichtian) of the Bauru Group, Paraná Basin (e.g. [84, 113]).

**Referred materials**: UFRJ-DG 451-R, a left hemimandible fragment with three preserved alveoli and one complete tooth; UFRJ-DG 501-R, a right hemimandible partially complete (the anterior portion with first three teeth missing), with eleven single alveoli, one alveolar groove partially preserved and with nine dental crowns remaining.

**Referred material locality**, **lithology and horizon**: AM1 site in the outskirts of the Alfredo Marcondes municipality, conglomerates of the upper interval of the Presidente Prudente Formation, Upper Cretaceous (Late Campanian–Early Maastrichtian) of the Bauru Group, Paraná Basin.

**Diagnosis**: *Roxochampsa paulistanus* comb. nov. is a platyrostral notosuchian with an unique set of characters: at least nineteen teeth per hemimandible (eighteen teeth for *Itasuchus* and *Pepesuchus*, and at last twenty two for *Caririsuchus*); festooned dentary formed by two waves, being the first between d4-d5 and the second and more smoothy between d8-d14 (similar feature is shared with the others Itasuchidae species); presence of two alveoli couplets in mid-anterior portion of the mandible (d6/d7 and d8/d9) separated by a small diastema (shared with *Itasuchus*); splenial well participating in the mandibular symphysis, anteriorly reaching to d5-d6 (shared with *Pepesuchus*); mandibular symphysis is long, and is as broad as high (shared with Itasuchidae species); last two mandibular teeth sit in an alveolar groove (autapomorphy among Itasuchidae species); interlocking teeth arrangement known as "crocodyloid occlusion" (shared with *Itasuchus* and *Pepesuchus*); rostral and mid dentary teeth with apicobasal high relief ridges fully crenulated by pseudo-denticles (autapomorphy among Itasuchidae species).

### Description of UFRJ-DG specimens

The mandible design of *Roxochampsa paulistanus* comb. nov. (UFRJ-DG 501-R) is similar to the ones of *Itasuchus jesuinoi* (DGM 434-R and MUGEO 218-V) and *Pepesuchus deiseae* (MN 7005-V and MCT 1788-R). These mandible profiles is moderately low and festooned by two smoothly waves, with first concavity at d6-d9 region, and the second at d13-d15 region. The mandibular symphysis comprises both dentaries and splenials and extends until d9 level.

#### Dentaries

The preserved right dentary of *Roxochampsa paulistanus* comb. nov. (UFRJ-DG 501-R) is moderate in anteroposterior length (33.7cm), corresponding to a mesorostrine species (“normal” rostrum length *sensu* [44]), and low between occlusal and ventral margins, pointing a platyrostral form.

**Fig 6:**
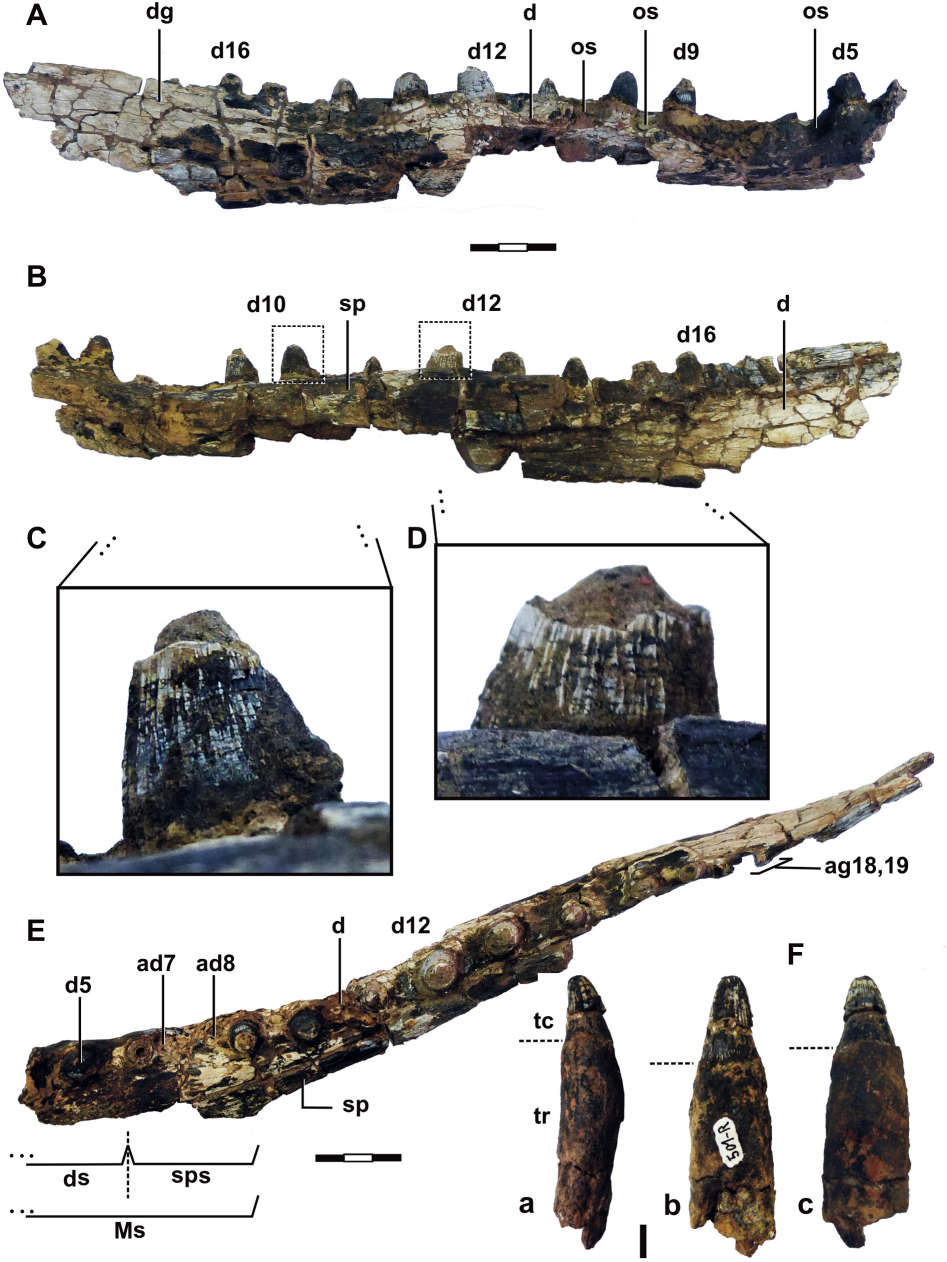
*Roxochampsa paulistanus* comb. nov. (UFRJ-DG 501-R). A- lateral view; B- medial view; C- detail, mesio-lingual surface view of tenth hemimandibular tooth; D- detail, lingual surface view of twelfth hemimandibular tooth; E- oclusal view; F- isolate tooth: a- lateral profile; b-lingual view; c- labial view. Each scale bar = 10 mm. Legend in text.

The mandibular symphysis region is moderately long, narrow, shallow and well-formed either by dentary and splenial, similarly to occur in *Itasuchus jesuinoi*, *Pepesuchus deiseae* and *Barcinosuchus gradilis*. The anteriormost hemimandible symphyseal portion is not preserved in UFRJ-DG 501-R, but, by the analysis of a partial alveolus in front of the fragment, and also by the alveolar size comparisons, we consider such missing portion as comprising the four first hemimandible teeth. Thus, we propose that the mandibular symphysis of *Roxochampsa paulistanus* has nine teeth (Fig 6), with five teeth in dentary symphysis and four in splenial.

The alveoli countdown points nineteen dentary teeth for *Roxochampsa paulistanus*, the last two settled in the alveolar groove. The dentary composes the medial osseous alveoli wall, but progressively lesser contribution front to rear, until the interalveolar region of d13 and d14 when it ceases and occurs splenial alveoli walls medially from this point (d14 to d18-19).

From an occlusal view, the dentary abrupt open and makes an angle about 25° at the end point of the mandibular symphysis, conferring a narrow "Y shape" for mandible of *Roxochampsa paulistanus*, with a straight lateral outline in mandibular symphysis region, and slightly bowed (convex) after it.

**Fig 7:**
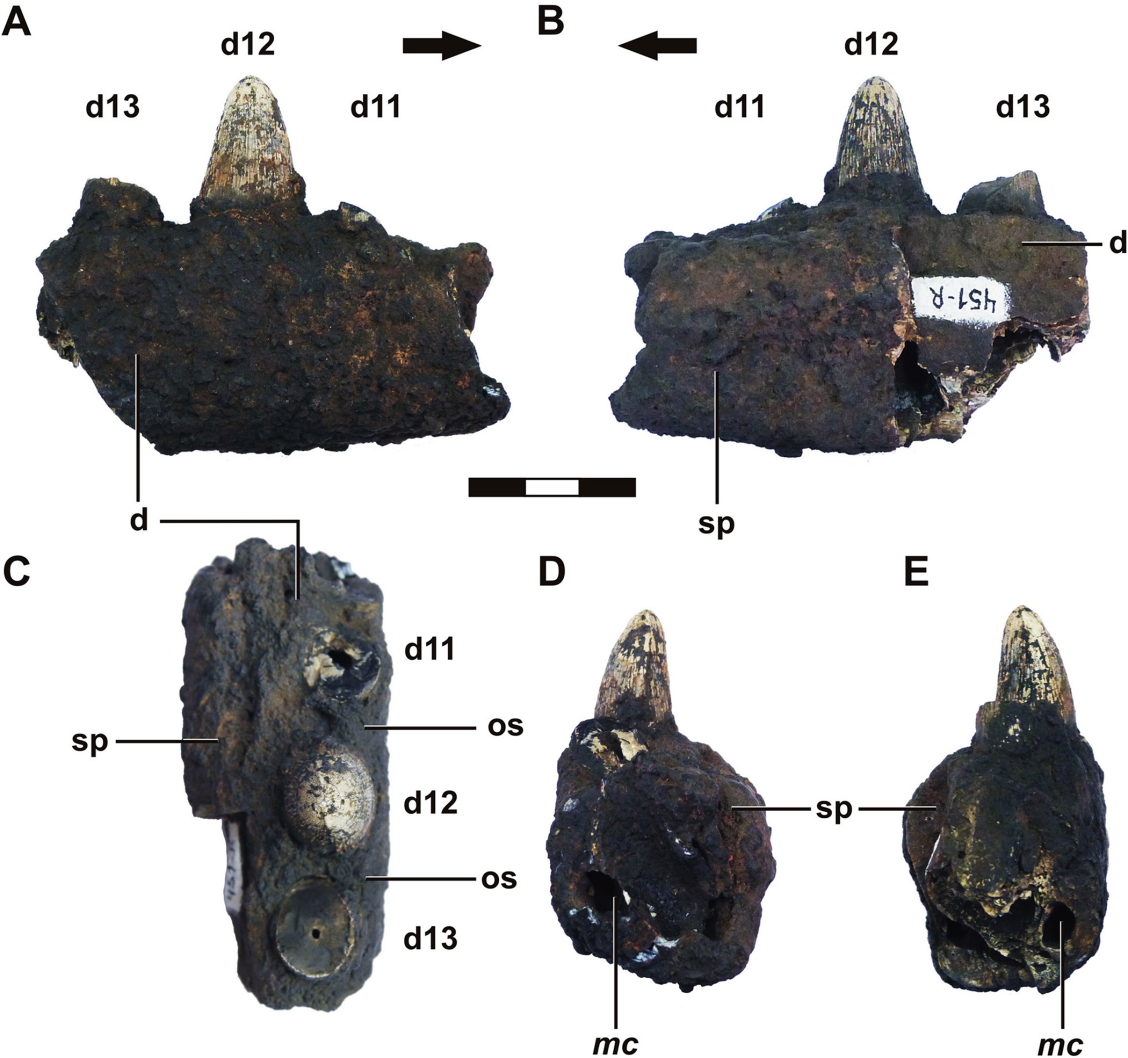
*Roxochampsa paulistanus* comb. nov. (UFRJ-DG 451-R). A- lateral view; B- medial view; C- oclusal view; D- anterior view; E- posterior view. Each scale bar = 10 mm. Legend in text.

Laterally, the dentaries of both specimens UFRJ-DG 451-R (Fig 7) and 501-R (Fig 6) and, are impregnated by a dark, moderate thick manganese crust which masks the dermal ornamentation. Even so, the sculpturing look as typical mesoeucrocodylian pattern: many pits for anterior portion, followed by a relatively smooth portion, being rugous by sulci pattern to the end of hemimandible. Similarly to crocodiles and other cretacic platyrostral species (i.e. *Itasuchus jesuinoi* and *Pepesuchus deiseae*) two sinusoidal waves gently festoon the mandible occlusal margin of *Roxochampsa paulistanus*. The first concavity is between d5-d9, with the second between d13,d14-d15.

Regardless of lacking cranial material preserved of *Roxochampsa paulistanus* comb. nov., the occlusal scars in the lateral surface of dentary near the occlusal edge (a feature shared with *Itasuchus*, but less marked in *Pepesuchus* specimens) indicate an interlocking arrangement for the teeth of the upper and lower jaw, producing an alternate occlusion.

The deep sulcus that crosses the dentary and surangular ending in a nutrient foramen at both ends (more developed and pronounced in metriorhynchidae thalattosuchians) is partially preserved in the dentary of *Roxochampsa paulistanus* UFRJ-DG 501-R.

**Fig 8:**
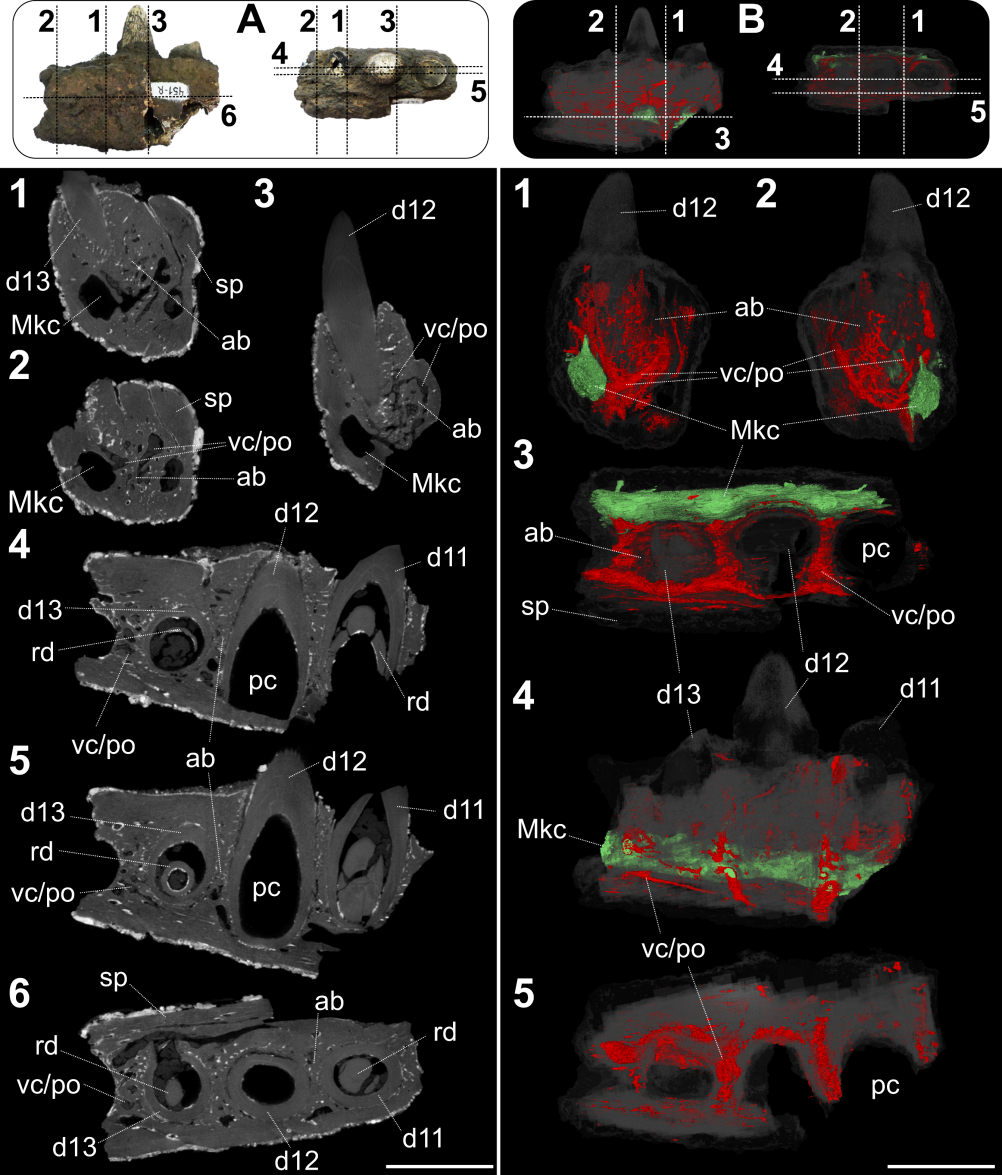
Internal structures of the fragmented hemimandible of *Roxochampsa paulistanus* comb. nov. UFRJ-DG 451-R from μCT-scan slices. Boxes on top indicate planes, while below the corresponding cuts from the fossil (A) and the 3D model (B). (A): A1-3- transverse planes and coronal cuts; A4-5- median planes and sagittal cuts; A6- frontal plane and horizontal cut. (B): B1-2- transverse planes and coronal cuts; B3-frontal plane and horizontal cut; B4-5- median planes and sagittal cuts. Scale bar = 2.5 cm.

The micro CT-scan of UFRJ-DG 451-R (*Roxochampsa paulistanus*) revealed four replacement teeth in the alveoli (three complete and one partial in the most posterior region), the Meckel’s channel and the vascularization in the interalveolar area (Fig 8). The suture between the splenial and the dentary is observable running from the occlusal to the alveolar cavities (see Fig 8A1 and 8A2) in the medial region. The Meckel’s channel (i.e. primordial channel of the mandible (*sensu* Iordansky [115]) extends along the dentary bone in the lateral region, below dentary alveolus. The alveolar bone exhibits vascular channels, which are medially connected with the Meckel’s channel and with the basalmost portion of the teeth alveolus.

The micro CT images of conoral, sagittal and horizontal slices from the *Roxochampsa paulistanus* (UFRJ-DG 451-R) presents some pneumatization in the dentary restricted to the interalveolar portions, revealed by conspicuous cancellous bone therein (Fig 8). The micro CT images from the parasagittal cuts show that Meckel's channel emits several intraosseous ramifications (Fig 8), probably to nourish the *cartilago Meckeli* and the *M*. *intramandibularis* (Mim according to Iordansky [116]).

Unfortunately, the posterior dentary region is not preserved, and so there are no evidence of an external mandibular fenestra at that point.

#### Splenials

Only the right splenial of UFRJ DG 501-R is partially preserved. It is shallow in the symphyseal region and comprises little more than one-third of this structure, comprising d6-d9 portion. However, the splenial is thick, robust and little medially convex in postsymphyseal hemimandible. The splenial makes the medial alveoli wall from the fourteenth to the last alveolus and alveolar groove (for d18-19). Unfortunately, due to the preservation, the intramandibular foramina were not preserved.

#### Dentition

Only dentary teeth and tooth fragments were preserved in both UFRJ-DG 451-R, a small fragment, and 501-R (except by a single isolated tooth, found it crossing the right hemimandible UFRJ-DG 501-R and presumably a tooth from the non-preserved upper jaw).

Despite the loss of the first dentary teeth in UFRJ-DG 501-R, the comparative analysis with many other related mesoeucrocodylian taxa points nineteen teeth for *Roxochampsa paulistanus* comb. nov. mandible, the last two teeth set together in a small alveolar groove.

From an occlusal view, the alveoli are normal to well interspaced. However there is a singular construction at symphyseal posterior level. In this region, the d7 and d8 are very small (the smallest from the tooth row) and contiguous to adjacents d6 and d9, with a short diastema between them (Fig 5).

The mandible tooth row vary in size until the d14, being gradually smaller toward the tooth row end. This tooth patterns is referred as moderately heterodonty and anisodonty (Iordansky [115] use the term "pseudoheterodonty" for size differences, but we prefer "anisodont" as such term by considering pseudoheterodonty as a non-precise descriptional term). There is a very small portion of right d4 alveolus preserved, and by its dimension with some broken of the anterior region, we consider a possible hypertrophied d4 tooth for *Roxochampsa paulistanus*. Commonly, this is the biggest, or one of them, in the mandible tooth row of mesoeucrocodylians, except in eusuchians gavialids as in *Kaprosuchus saharicus*, many small and omnivorous notosuchians (e.g. uruguaysuchids and advanced notosuchians according to Pol et al. [31]), and thalattosuchians. Once the d4 is lost, the d12 is the bigger tooth of the mandible row, being d7 and d8 the smaller ones.

All preserved teeth of *Roxochampsa paulistanus* are monocuspided with blunt and round apices, with just a slightly acute apex on top. The preserved teeth have sub-conical crowns but are circular in basal section. The neck or the region between tooth crown and root (*cervix dentis* [114]) has a discreet constriction, different from the accentuated condition found in many peirosaurids and some eusuchians taxa in which the neck is more pronounced.

**Fig 9:**
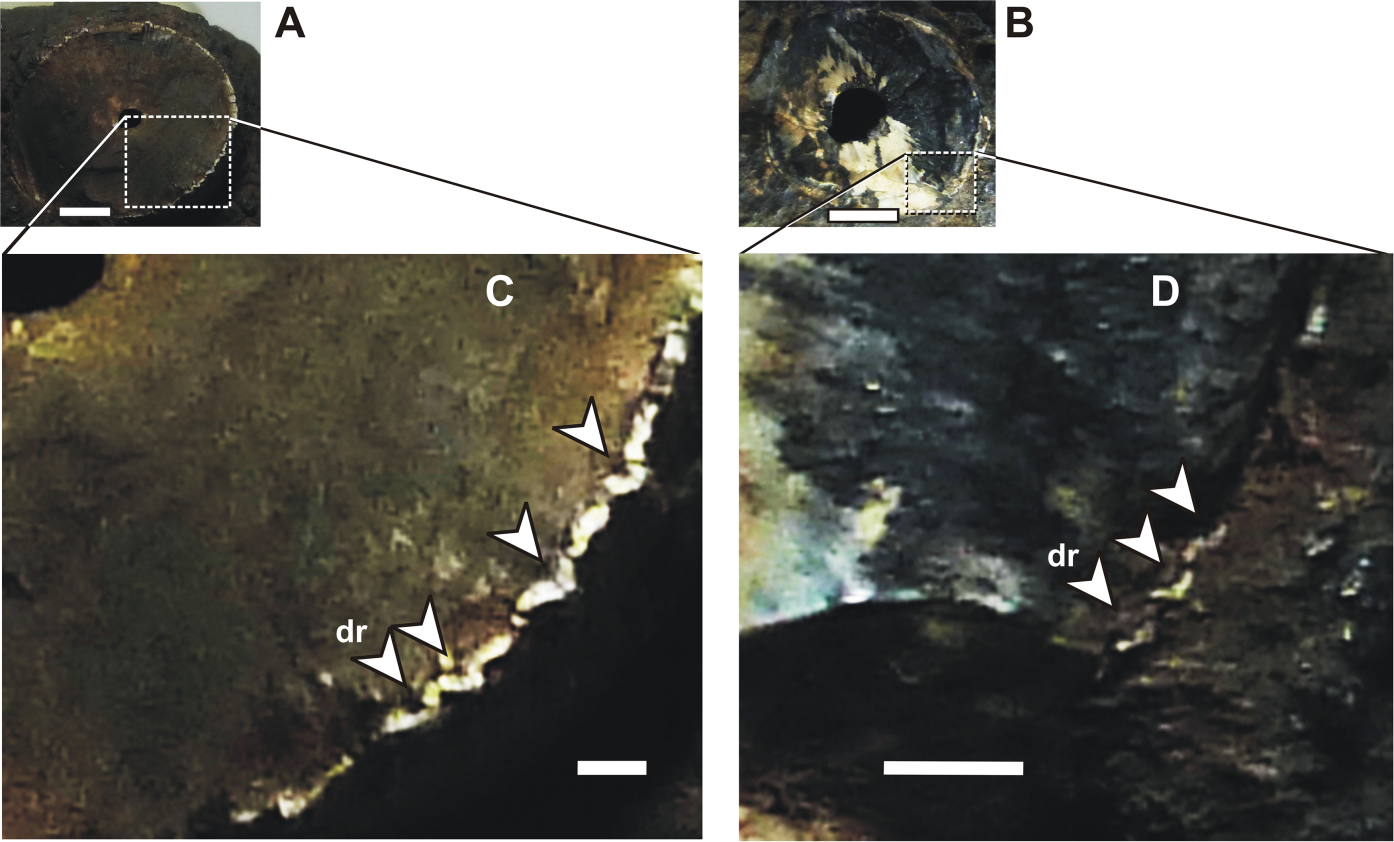
*Roxochampsa paulistanus* comb. nov. UFRJ-DG 451-R. The enamel and dentine of the d11 and d13 broken crowns. A- d11; B- d13. Scale bar = 1 mm.

The crown surfaces of rostral [114] and middle mandibulary teeth are labio-lingually asymmetric, with the labial surface wider and slightly convex due to a lingual curvature (lingual surface little concave or even straight). The taxon presents the main, mesial and distal crown carinae serrated by fine crenulations formed by interaction of crease enamel ornamentation from the main body of the crown surface in a "false ziphodont teeth" pattern [117], not formed by truly individualized denticles. In the crown surface of *Roxochampsa paulistanus* the enamel is fully fluted and ornamented by many subparallel and irregular longitudinal high relief ridges (apicobasal keels/ridges) that run from base to mid crown surface as subparallel crests and some braided at the base, as lesser secondary carinae. Such keels are formed by both enamel and dentine (Fig 9).

**Fig 10:**
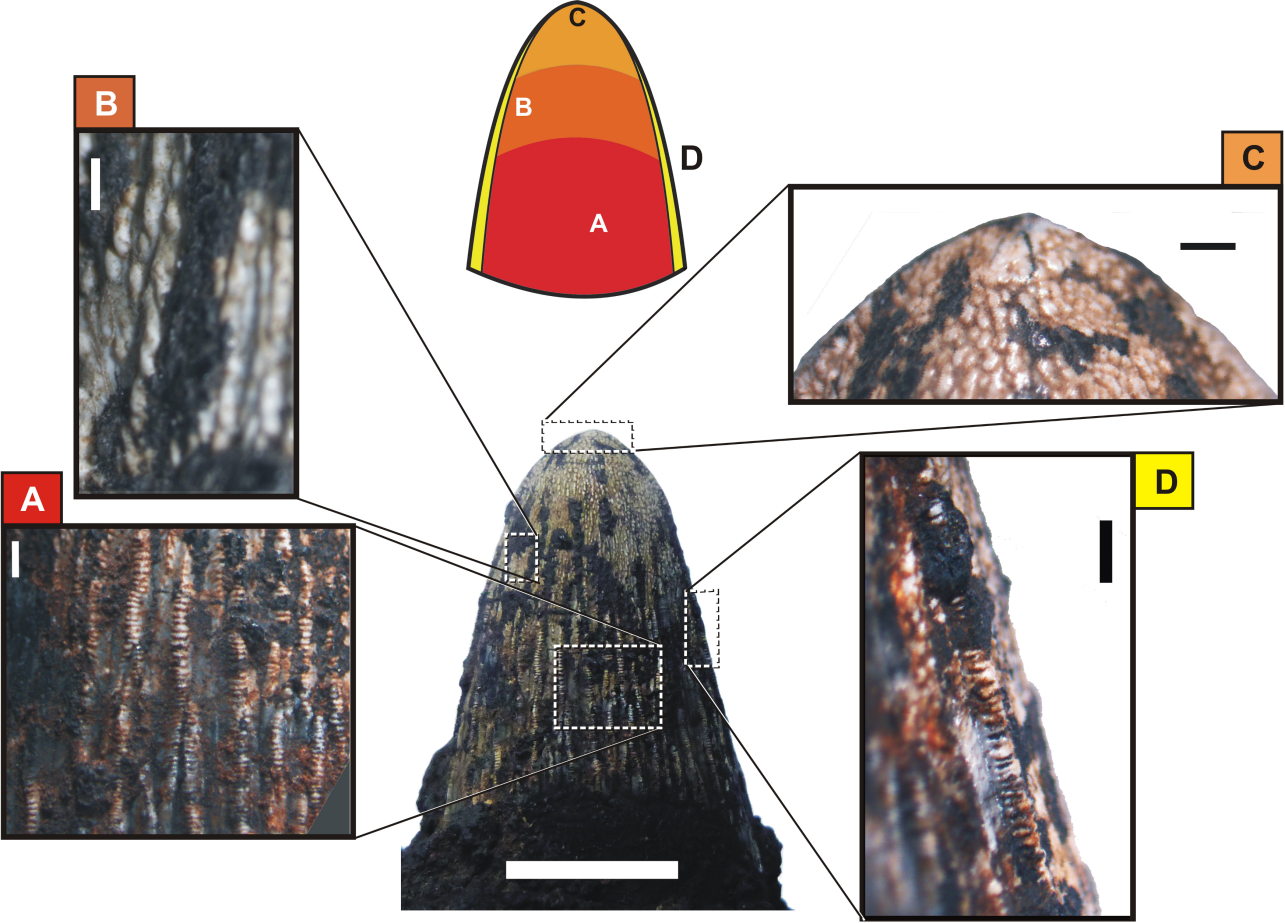
*Roxochampsa paulistanus* comb. nov. UFRJ-DG 451-R, d12 multicrenulate tooth features. A- detail of the basal region, showing multicrenulated high relief enamel ridges; B- detail of apical region, showing granulated protuberances “scaly-forms”; C- detail of mid-apex region, showing overlapping projections; D- section of distal and crenulated carena. Scale bar for the tooth = 10 mm; in A, C and D scale bars = 1 mm; in B scale bar = 0.1mm.

Not for all but mainly for the great anteromedian teeth from the row, especially developed in the great d12 of UFRJ-DG 451-R, those irregular and longitudinal ridges are even crenulated, but differently from the observed to the main carinae the crenulations from apicobasal ridges are more coarse and formed by enamel pseudo-denticles (according to [131]), which are better individualized (Fig 10) and producing a serration like morphology too [131, 132]. In UFRJ-DG 451-R and 501-R, these multiple crenulated and apicobasally aligned ridges are better present in the lingual enamel surface, and also better formed from the base to the middle portion of the crown, present until the middle-upper region of the crown.

From middle through last quarter of the d12 tooth crown (DGM 258 and 259-R, UFRJ-DG 451-R), the apicobasal kells give rise to another dental feature, being like blunt hooked and overlapped structures (Fig 10). These vertical lines of imbricate hooked structures progressively subside and give way to some rugous and “scaly forms” feature to the tooth apex (Fig 9).

In general, the *Roxochampsa paulistanus* teeth (mainly DGM 259-R, UFRJ-DG 451-R and UFRJ-DG 501-R) are characterized by: (*i*) conical and circular in section; (*ii*) neck slightly constricted; (*iii*) asymmetric crown surfaces; (*iv*) mesial and distal main carinae crenulated by false denticles; (*v*) crown surfaces ornamented by many high relief crenulated apicobasal ridges formed by pseudo-denticles; (*vi*) longitudinal lines of imbricate hook like structures from mid to top; and (*vii*) rugous and “scaly form” tooth apex.

The μCT-scan images of UFRJ-DG 451-R revealed three complete alveolus, with two replacement teeth in the eleventh and the thirteenth dentary alveolus (Fig 8A4–8A6). They exhibit long and wide tooth root, which occupies about 85% of the height of the hemimandible. The μCT-scan analysis revealed lines of dentine deposition (see μCT-scan Fig 8A3–8A6). These evident dentine lines suggest events of variation in the dentine deposition along the teeth formation (e.g. [133–137]). The eleventh tooth presents two defined lines, with a ratio between the length of the dentine line to the pulp cavity and the thickness of the tooth (Rdt) comprises, from inside to outside, 0.259 and 0.618 respectively, in coronal slice, and 0.277 (only to the first line) in sagittal slice (Fig 8). The second line in coronal slice was not evident in the μCT-scan image in the mid region of the teeth. The twelfth tooth is the one that preserved more dentine lines, comprising five lines. The coronal slice reveals the Rdt of the lines comprising 0.056, 0.249, 0.414, 0.722 and 0.780, while in the sagittal was 0.075, 0.240, 0.627, 0.707 and 0.798. The thirteenth tooth comprised three lines. The coronal slice resulted in a Rdt with 0.318, 0.571 and 0.744, while the Rdt of the sagittal slice was 0.353, 0.705 and 0.737. The record of the dentine lines, the Rdt and the replacement teeth in the eleventh and thirteenth alveolus corroborates the “wavy pattern” of tooth replacement (with alternate, neighboring, teeth being replaced in separate waves), commonly found among extant crocodylians [138].

### Main phylogenetic results

**Fig 11:**
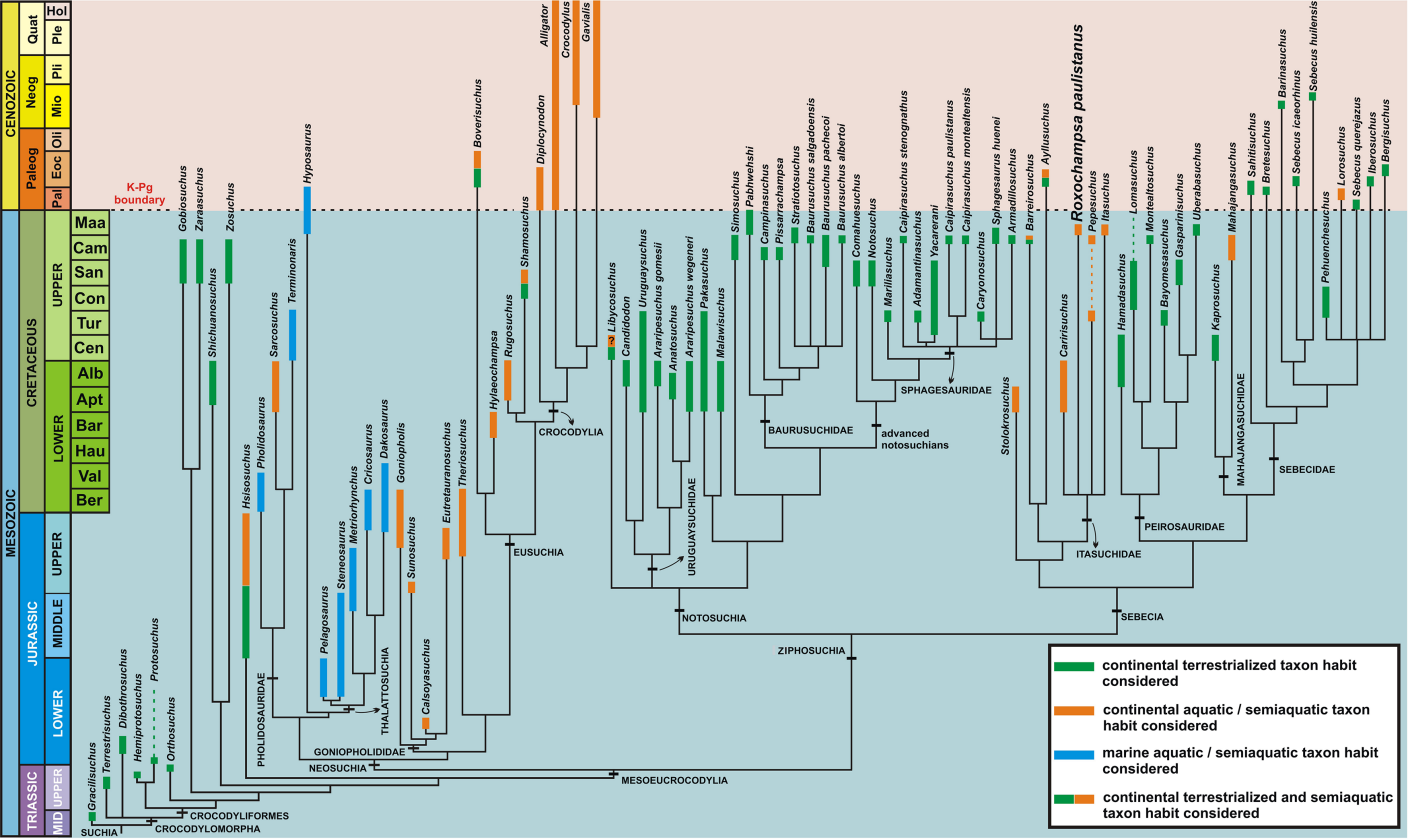
Calibrated strict consensus tree from 225 MLTs (1520 steps, CI = 0.304 and RI = 0.692). The phylogenetic relationships (thin lines) come from the strict consensus tree calibrated with the geological ages (chronostratigraphic chart). Thick lines represents the species temporal range based on specimens occurrence. Also, colors in thick lines represent the inferred habit for studied species.

Our phylogenetic analysis resulted in a consensus cladogram (Fig 11), from 225 minimum-length trees (MLTs) with 1520 steps, and a well-resolved topology (CI = 0.304 and RI = 0.692).

The clade Mesoeucrocodylia was supported in all trees by: quadratojugal dorsal process narrow, contacting only a small part of postorbital (char. 17: 1 -> 0); palatines firmly sutured to pterygoids on anterior region of secondary palate (char. 183: 0 -> 1); postorbital bordering infratemporal fenestra (char. 187: 0 -> 1); and absence of a “primary palate” (choanal opening between maxillaries and palatines [char. 195: 1 -> 0]).

Neosuchia is monophyletic, supported in all trees by: external naris dorsally separated by a premaxillary bar from anterior tip of the rostrum (char. 6: 0 -> 2 [independently shared with *Lorosuchus*, in some trees, and in all trees with Mahajangasuchidae]); posterolateral process of squamosal poorly developed and projected horizontally at the same level of the skull roof (char. 33: 2 -> 0); external nares confluent (char. 60: 0 -> 1 [independently shared with advanced notosuchians]); closure of antorbital fenestra (char. 61: 1 -> 2 [independently shared with clade Baurusuchidae plus advanced notosuchians and Sebecidae minus *Sahitisuchus*]); dorsal osteoderms with a well-developed anterolateral process (char. 87: 0 -> 1); verticalized basioccipital (char. 102: 1 -> 0 [independently shared with Itasuchidae and its close related species]); vertebral centra spool shaped (char. 103: 0 -> 1); the insertion of the *m*. *iliotibialis* narrow and facing dorsally or slightly laterodorsally in ilium (char. 106: 1 -> 0); quadrate distal end with only one plane facing posteriorly (char. 140: 1 -> 0); dentary not compressed at the lateroventral surface anterior to mandibular external fenestra (char. 150: 0 -> 1 [independently shared with Itasuchidae and its close related species and with *Zosuchus* and *Lorosuchus*]); absence of a large nutrient foramen on palatal surface of premaxilla-maxilla contact (char. 265: 1 -> 0 [independently shared with advanced notosuchians minus *Comahuesuchus*]); tibial articular surfaces with medial and lateral regions subequally extended, with distal margin subhorizontally oriented (char. 316: 0 -> 1); and supraoccipital lateromedially occupying less than one third of the lateromedial width of the occipital surface (char. 337: 0 -> 1).

The clade Ziphosuchia was resurrected, and is supported in all trees by: parieto-postorbital suture excluded from dorsal surface of skull roof, being broadly visible within supratemporal fossa (char. 21: 0 -> 1); posterior premaxillary teeth smaller than the anterior ones (char. 72: 0-> 2); only the posterior cervical neural spines are rodlike (char. 81: 0 -> 1); hypapophysis in cervicodorsal vertebrae present up to the fourth dorsal vertebrae (char. 82: 0 -> 4); notch in premaxilla on lateral edge of external nares present on the dorsal half (char. 113: 0 -> 1); prezygapophyses of axis exceeding the anterior margin of neural arch (char. 142: 0 -> 1); anterior margin of femur at the area of insertion of *m*. *puboischiofemoralis internus 1* (PIFI1) and *m*. *caudofemoralis longus* (CFL) bearing a distinct flange and a marked concavity above this region (char. 147: 0 -> 1); cheek teeth constricted between the root and crown (char. 152: 0 -> 1 [independently shared with the clade *Theriosuchus* plus Eusuchia]); short paroccipital process lateral to cranioquadrate passage (char. 253: 1 -> 0 [independently shared with the clade *Calsoyasuchus* plus *Eutretauranosuchus* and *Hylaeochampsa*]); proximal-most portion of fibular head very sharply projecting posteriorly, forming a distinct extension (char. 257: 0 -> 1); prezygapophyseal process of anterior cervical vertebrae projects dorsally and is medially recurved (char. 276: 0 -> 1); abrupt change in position of parapophysis, with fourth dorsal vertebra (4dv) bearing the parapophysis at the neurocentral suture with the 5dv with parapophysis leveled with diapophysis forming a transverse process (char. 279: 0 -> 1); presence of a distinct rounded depression on the dorsal surface of neural arches of the anterior to mid dorsal vertebrae, located between the base of the neural spine and the postzygapophyseal process (char. 282: 0 -> 1); scapular blade is very broad and greater than twice the length of the scapulocoracoid articulation (char. 285: 0 -> 1); presence of a circular depression on the posterior surface of the proximal end of the humerus, related to the insertion of the *m*. *scapulohumeralis caudalis* (char. 294: 0 -> 1); fibular facet on the astragalus trapezoidal with the proximodistal height of its anterior margin lower than the posterior margin (char. 322: 1 -> 2); foramen *intramandibularis oralis* located on the anterior region of splenial (char. 346: 0 -> 1); and medial flange of the retroarticular process facing medially and strongly deflected (char. 356: 0 -> 1 [independently shared with *Crocodylus*]).

Within Ziphosuchia two clades are recovered, Notosuchia and Sebecia. The first, Notosuchia, is supported in all trees by: absence of a ventrally opened notch on ventral edge of rostrum at premaxilla-maxilla contact (char. 8: 1 -> 0 [reversal in Baurusuchidae and independently shared with some neosuchian clades]); anterior dentary teeth opposite premaxilla-maxilla contact no more than twice the length of other dentary teeth (char. 74: 1 -> 0 [reversal in Baurusuchidae and independently shared with some Neosuchia clades]); presence of a small neurovascular foramen located in the premaxillo-maxillary suture on the lateral surface of the rostrum (char. 125: 0 -> 1 [independently shared with *Protosuchus* and *Iberosuchus*]); presence of large and aligned neurovascular foramina on lateral maxillary surface near occlusal margin (char. 128: 0 -> 1 [reversal in *Stratiotosuchus* and independently shared with *Dibothrosuchus*, *Protosuchus* and *Sichuanosuchus*]); some maxillary teeth are implanted in a dental groove (char. 154: 0 -> 1 [reversal in some sphagesaurids, and independently shared with *Zosuchus*]). Also, in some trees the follow synapomorphies are recovered: the rugose surface for the insertion of the *M*. *iliotibialis*, that forms the supracetabular crest lateromedially, broad and rugose, and highly deflected laterally to form a remarkably deep acetabulum (char. 106: 1 -> 2); supraoccipital dorsally visible and penetrating through skull roof (char. 161: 0 -> 2 [independently shared with some sebecids, mahajangasuchids and close related taxa]); perinarial fossa extensive, with a distinctly concave surface facing anteriorly (char. 213: 0 -> 1 [reversal in Sphagesauridae]); and ventral margin of the postacetabular process horizontally or slightly posteroventrally deflected (char. 307: 0 -> 1).

The second group within Ziphosuchia is Sebecia, which is supported in all trees by: pterygoids forming posterior, lateral and part of the anterior choanal margin (char. 40: 0 -> 1 [independently shared with the clade *Araripesuchus* plus *Anatosuchus*, *Mariliasuchus*, *Campinasuchus* and Pholidosauridae]); lateral surface of the anterior region of surangular and posterior region of dentary with a deep and well-defined longitudinal groove (char. 108: 0 -> 1 [independently shared with Crocodylia]); surangular forms approximately one-third of the glenoid fossa and quadratojugal contributes to the lateral articular condyle, “double articulation” according to Buffetaut [139] (char. 146: 0 -> 1 [independently shared with some uruguaysuchids and *Hyposaurus*]); dentary with lateral concavity for the reception of the enlarged maxillary tooth (char. 148: 0 -> 1 [independently shared with *Araripesuchus gomesii*]); sinusoidal, with two concave waves (char. 149: 0 -> 3 [independently shared with Goniopholididae, Eusuchia and related taxa]); splenial robust dorsally posterior to symphysis, being much broader than the lateral alveolar margin of the dentary at the same region (char. 151: 0 -> 1 [reversal in Mahajangasuchidae, and independently shared with Goniopholididae]); jugal portion of postorbital bar anteriorly continuous but posteriorly inset (char. 157: 0 -> 1 [independently shared with *Pakasuchus*]); lateral contour of snout in dorsal view sinusoidal (char. 167: 0 -> 1 [independently shared with *Araripesuchus* and *Anatosuchus* clade, and the clade formed by Goniopholididae, Eusuchia and related taxa]); anterior half of palatines between suborbital fenestrae flared anteriorly (char. 260: 0 -> 1 [reversal in *Kaprosuchus*, and independently shared with notosuchians as *Uruguaysuchus*, *Malawisuchus* and *Caipirasuchus*, and even neosuchians such as *Sarcosuchus*, Goniopholididae, Crocodylia and it sister clade formed by *Rugosuchus* and *Shamosuchus*]); first and second premaxillary teeth nearly confluent (char. 264: 0 -> 1 [reversal in Sebecidae taxa]); and premaxilla-maxilla lateral fossa excavating alveolus of last premaxillary tooth (char. 267: 0 -> 1 [reversal in *Mahajangasuchus*]).

The species *Roxochampsa paulistanus* is recovered in a polytomy with *Barreirosuchus*, *Pepesuchus*, *Itasuchus* and *Peirosaurus*. This clade is here referred to as Itasuchidae and is supported by the follow states in all trees: unsculptured region along alveolar margin on the lateral surface of maxilla (char. 98: 0 -> 1); absence of a notch in premaxilla on lateral edge of external nares (char. 113: 1 -> 0 [reversal from ziphosuchian condition, and independently shared with *Anatosuchus* and Mahajangasuchidae]); trapezoidal skull roof (char. 170: 0 -> 1); and, in lateral view the ventral edge of maxilla is sinusoidal (char. 172: 0 -> 1 [highly homoplastic, being independently shared with, *Araripesuchus*, *Anatosuchus*, Peirosauridae, Mahajangasuchidae, basal sebecids as *Sahitisuchus* and *Bretesuchus*, and one of the original characters proposed by Benton & Clark [71] for the clade Neosuchia]).

The clade ((*Barreirosuchus* + *Ayllusuchus*) Itasuchidae) has as its sister species *Stolokrosuchus*, supported in all trees by: the posterolateral process of squamosal is elongated, thin, and posteriorly directed, not ventrally deflected (char. 33: 2 -> 1 [independently shared with *Hamadasuchus*]); basioccipital and ventral part of otoccipital posteroventrally oriented (char. 102: 1 -> 0 [independently shared with Neosuchia]); dentary surface dorsoventrally compressed anterior to mandibular external fenestra (char. 150: 0 -> 1 [independently shared with *Zosuchus*, *Lorosuchus* and Neosuchia]); anterorbital fenestra low and elongated, slit-like (char. 268: 0 -> 1 [independently shared with Mahajangasuchidae]); presence of apicobasal ridges on the enamel surface of posterior teeth (char. 363: 0 -> 1 [independently shared with some neosuchians and advanced notosuchians]); and, presence of a sagittal torus on maxillary palatal shelves (char. 384: 0 -> 1 [independently shared with *Bretesuchus*, *Pabhwehshi* and *Hamadasuchus*]).

The clade (Peirosauridae (Mahajangasuchidae + Sebecidae)) is supported in all trees by following features: in ventral view, splenial forms 20% of the symphyseal length (char. 71: 2 -> 1 [independently shared with the clade (Baurusuchidae + advanced notosuchians) and reversed in *Lorosuchus* and *Sahitisuchus*]); in lateral view, the ventral edge of maxilla is sinusoidal (char. 172: 0 -> 1 [highly homoplastic, being independently shared with *Araripesuchus*, *Anatosuchus*, Itasuchidae, and one of the original characters proposed by Benton & Clark [71] for the clade Neosuchia]); and, internal carotid artery located dorsally, close to and within the same depression as the foramina for the cranial nerves IX-XI (char. 338: 0 -> 1 [independently shared with the clade (Baurusuchidae + advanced notosuchians) and *Rugosuchus*]).

Peirosauridae includes (*Hamadasuchus* ((*Lomasuchus*, *Montealtosuchus*) (*Bayomenasuchus* (*Gasparinisuchus* + *Uberabasuchus*))), is supported by: presence of a foramen in perinarial depression of premaxilla (char. 224: 0 -> 1); maxilla-palatine suture with palatine anterior end slightly invaginated (char. 230: 0 -> 2); anterior process of quadratojugal from long, less than half length of infratemporal bar, to moderate, one-third of infratemporal bar (char. 275: 0 -> 1, independently shared with Shagesauridae and *Stolokrosuchus*). For some trees, the pronounced unevenness between palatal surface and alveolar margin at level of sixth or seventh alveolus (char. 371: 0 -> 1), is found as a synapomorphy.

The clade (Mahajangasuchidae + Sebecidae) is supported in all trees by the follow features: slightly grooved ornamentation on external surface of dorsal cranial bones (char. 1: 2 -> 1 [independently shared with the clade (*Gobiosuchus* + *Zaraasuchus*) and the clade ((*Pakasuchus* + *Malawisuchus*) (*Simosuchus* (Baurusuchidae + advanced notosuchians))) ]); supraoccipital exposed dorsally on skull roof (char. 161: 0 -> 2 [independently shared with *Ayllusuchus*, *Diplocynodon*, *Caririsuchus* and in some trees with Notosuchia]); ectopterygoid medial process forked, with an accessory anteromedial branch reaching the palatine and forming part of the lateral margin of the choanal opening (char. 168: 0 -> 1); quadrate contacts basioccipital ventrally to the braincase surface (char. 336: 0 -> 1 [independently shared with (*Zosuchus* + *Shichuanosuchus*)]); and, suborbital region of jugal separated by a notch from infratemporal bar of jugal (char. 374: 0 -> 1).

The Mahajangasuchidae, which is composed of *Kaprosuchus* and *Mahajangasuchus*, is supported by: broad premaxilla anterior to nares (char. 5: 0 -> 1 [independently shared with *Simosuchus*, *Goniopholis*, the clade (Pholidosauridae (*Hyposaurus* + Thalattosuchia)) and in some trees with *Lorosuchus*]); external nares dorsally exposed and separated by anterior tip of rostrum by a premaxillary bar (char. 6: 0 -> 2 [independently shared with Neosuchia and in some trees with *Lorosuchus*]); nasal does not contact lacrimal (char. 10: 0 -> 1 [independently shared with *Orthosuchus*, *Pakasuchus*, *Sunosuchus*, *Shamosuchus*, *Hylaeochampsa*, the clade (*Anatosuchus* + *Araripesuchus wegeneri*) and the clade ((*Lomasuchus* + *Montealtosuchus*) (*Bayomenasuchus* (*Gasparinisuchus* +*Uberabasuchus*)))]); parietal narrow between the supratemporal fenestrae (char. 31: 0 -> 1 [highly homoplastic and independently shared with *Stolokrosuchus*, *Goniopholis*, *Terminonaris*, the clade (*Boverisuchus* + *Hylaeochampsa*), the clade (*Simosuchus* (Baurusuchidae + advanced notosuchians)), the clade (*Lomasuchus* + *Montealtosuchus*) and in some trees with *Dibothrosuchus* and the clade (*Hemiprotosuchus* + *Protosuchus*)]); posterolateral process of squamosal is posterodorsally deflected (char. 33: 2 -> 4); *M*. *pterygoideous posterior* extends onto lateral surface of angular (char. 70: 0 -> 1 [independently shared with baurusuchids, *Sunosuchus* and Crocodylia]); jaw joint below the level of toothrow (char. 96: 1 -> 2 [independently shared with *Orthosuchus*]); absence of a notch in premaxilla on lateral edge of external nares (char. 113: 1 -> 0 [independently shared with *Anatosuchus* and Itasuchidae]); premaxilla-maxilla suture in palatal view, medial to alveolar region, posteromedially directed (char. 116: 0 -> 2 [independently shared with *Comahuesuchus* and *Simosuchus*]); the postorbital process of jugal posteriorly positioned (char. 133: 1 -> 2 [independently shared with the clade composed by *Araripesuchus* species and *Anatosuchus*]); the lateral edges of dentary symphysis, in ventral view, longitudinally oriented, convex anterolateral corner, and extensive transversely oriented anterior edge (char. 144: 0 -> 2 [independently shared with *Simosuchus*]); thin splenial posterior to the symphysis (char. 151: 1 -> 0); T-shaped bar expanded ventrally on choana (char. 180: 0 -> 1 [independently shared with *Uruguaysuchus* and *Araripesuchus gomesii*]); nasal bones partially or completely fused (char. 244: 0 -> 1 [independently shared with *Hyposaurus*, *Rugosuchus*, *Caririsuchus* and the clade (*Pissarrachampsa* (*Stratiotosuchus* + *Baurusuchus salgadoensis* + *Baurusuchus pachecoi* + *Baurusuchus albertoi*))]); antorbital fenestra low and elongated, slit-like (char. 268: 0 -> 1 [independently shared with the *Stolokrosuchus* and *Pepesuchus*]); the jugal dorsal margin of anterior and posterior processes at a sharp angle to one another, both processes slope ventrally to form a strongly arched jugal (char. 274: 0 -> 1); jugal-ectopterygoid contact confluent with lateral jugal margin (char. 373: 0 -> 1); and, anterior tip of mandible, in lateral view, abrupt convex (char. 388: 0 -> 1 [independently shared with Bretesuchus, the clade (*Zosuchus* + *Shichuanosuchus*), the clade (*Terminonaris* + *Sarcosuchus*), the clade (*Steneosaurus* + *Pelagosaurus*), the clade ((*Rugosuchus* + *Shamosuchus*) Crocodylia), the clade (*Anatosuchus* + *Araripesuchus wegeneri*), and the clade ((*Lomasuchus* + *Montealtosuchus*) (*Bayomenasuchus* (*Gasparinisuchus* +*Uberabasuchus*))) and in some trees with *Libycosuchus*]).

The clade Sebecidae, which includes (*Sahitisuchus* (*Bretesuchus* ((*Barinasuchus* (*Sebecus icaeorhinus* + *Sebecus huilensis*)) (*Lorosuchus* + *Sebecus querejazus* + *Iberosuchus* + *Bergisuchus* + *Pehuenchesuchus*)))), is supported by the features: external surface of maxilla with a single plane facing laterally (char. 129: 1 -> 0); base of postorbital process of jugal anterodorsally oriented (char. 132: 0 —> 1 [independently shared with *Notosuchus*]; foramen *intermandibularis oralis* is large and slit like, with their anteroposterior length being approximately or more than 50% of the depth of the splenial (char. 163: 0 -> 1); sharp ridge on the lateral surface of the angular (char. 206: 0 -> 1 [independently shared with *Sichuanosuchus*, *Simosuchus*, the clade (*Gobiosuchus* + *Zaraasuchus*), and basal eusuchians as *Shamosuchus* and *Rugosuchus*]); and, first and second premaxillary teeth not confluent (char. 264: 1 -> 0).

## Discussion

### Anatomical comments and comparisons

Following Busbey’s III proposition [44], we consider as a taxon to have the platyrostral morphotype if it exhibits a flat-snouted rostrum shape, in contrast to the oreinirostral condition (“hill-like snout”). In Busbey’s classification, platyrostral are subdivided into *tubular*: dorso-ventral and lateromedial diameters are subequal; *broad*: lateromedial diameter more than twice the dorsoventral one; and *narrow*: lateromedial diameter between 1.2 and 1.9 times the dorsoventral one [44]. Therefore, few known mesoeucrocodylian taxa recovered from Bauru Group are identified as platyrostral forms, and follow: *Itasuchus jesuinoi* (snout inferred to be narrow by mandible profile); *Pepesuchus deiseae* (narrow snout); *Barreirosuchus franciscoi* (snout inferred to be broad snout by preorbital region shape); and *Roxochampsa paulistanus* (inferred to be narrow by hemimandible shape). Most mesoeucrocodylian species in the Bauru Group are nonplatyrostral and considered as a more terrestrialized form, such as peirosauromorphs, advanced notosuchians (*sensu* [31]) and baurusuchids (e.g. [140, 141]).

The mandible of all itasuchids (here considered as *Caririsuchus*, *Itasuchus*, *Pepesuchus* and *Roxochampsa*) have a long mandibular symphysis that is as broader as high. The alveolar count of *Roxochampsa* differs from other itasuchids, which has nineteen alveolar teeth with last two inserted in an alveolar groove (an autapomorphic feature within itasuchids), while *Pepesuchus* and *Itasuchus* have eighteen alveoli without the alveolar groove. *Caririsuchus* has at least twenty two alveoli, but it was drawned with more than twenty mandibulary teeth both in Kellner [77] as in Buffetaut [47], and in the absence of available material, it is dubiously accepted. There are in *Roxochampsa* and *Itasuchus* two alveoli couplets in mid-anterior portion of the mandible (d6/d7 and d8/d9) separated by a small diastema, which is shared with the left side of *Pepesuchus* (MN 7005-V) a case of asymmetry.

**Fig 12:**
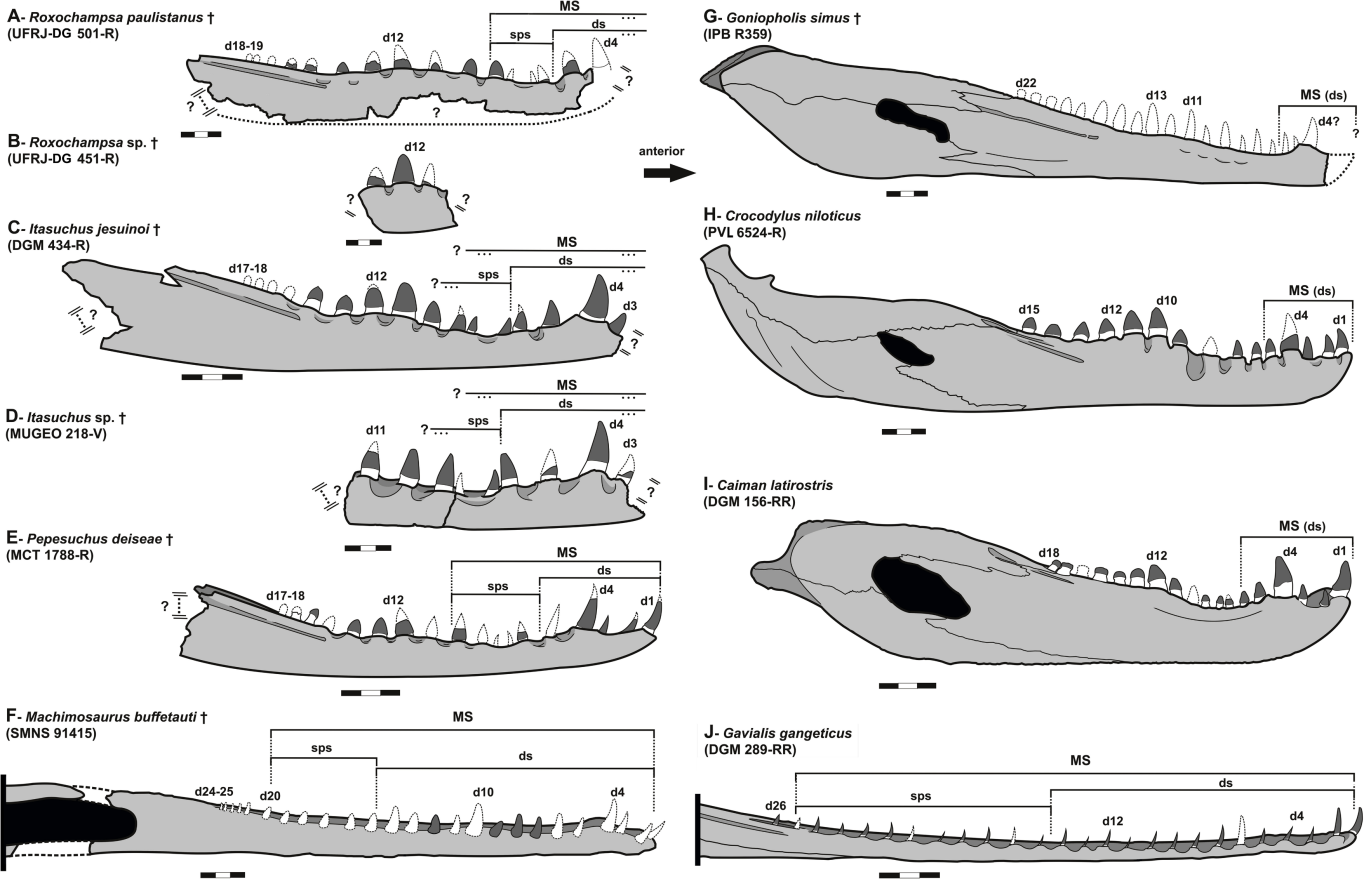
Hemimandible comparisons between some mesoeucrocodylian taxa. A- *Roxochampsa paulistanus* comb. nov. (UFRJ-DG 451-R), right hemimandible fragment; B- *Roxochampsa paulistanus* comb. nov. (UFRJ-DG 501-R), right hemimandible, lacking the anterior-most portion; C- *Itasuchus jesuinoi* (DGM 434-R), left hemimandible fragment mirrored; D- *Itasuchus* sp. (MUGEO 218-V), right hemimandible fragment; E- *Pepesuchus deiseae* (MCT 1788-R), left hemimandible fragment mirrored; F- *Machimosaurus buffetauti* (SMNS 91415) right hemimandible; G- *Goniopholis simus* (IPB R359), right hemimandible; H- *Crocodylus niloticus* (PV. 6524-R), right hemimandible; I- *Caiman latirostris* (DGM 156-RR), left hemimandible mirrored, J- *Gavialis gangeticus* (DGM 289-RR) right hemimandible. Each scale bar = 10 mm. Legend in text.

There are two sinusoidal waves of enlarged teeth (character it also supports Neosuchia according Benton & Clark [71]), with the mandible occlusal margin of *Roxochampsa* exhibiting two slight undulations, the first between d4–d5, and the second and more smoothy between d8–d14, a state shared with *Caririsuchus*, *Itasuchus jesuinoi* (DGM 434-R), *Itasuchus sp*. (MUGEO 218-V) and *Pepesuchus*, being the second wave less developed in *Pepesuchus*. The posterior dentary inclination, posterior to the second wave, is less accentuated in *Roxochampsa* than to in *Itasuchus* and *Pepesuchus* (Fig 12).

The upper and lower jaw teeth interlock in an arrangement known as "crocodyloid occlusion", a common feature for narrow platyrostral forms. Within Itasuchidae it is found in *Roxochampsa*, *Itasuchus* and *Pepesuchus*. Concerning to *Caririsuchus*, the available ilustrations shows a platyrostral itasuchid with a high tooth count per hemimandible (up to twenty), the teeth are closely spaced and lack clear oclusal scars, leading us to consider this taxon as having an “overbite occlusion”, more frequently found in more broaded platyrostral morphotypes.

Despite the left splenial not being preserved in UFRJ-DG 501-R, based on its scars and the dentary medial morphology the splenials of *Roxochampsa paulistanus*significantly contributes to a moderate length of the mandibular symphysis (totally comprising d1-d9), reaching the d6 at mesial carena tooth level, ending at d9 distal carena tooth level.The splenial anterior limit in the mandibular symphysis is not the same between *Roxochampsa paulistanus* and *I*. *jesuinoi* and *P*. *deiseae*. In *Itasuchus jesuinoi* (both DGM 434-R and MUGEO 218-V) the splenial anteriorly extends until d6 at distal carena tooth level, and in *P*. *deiseae* (MCT 1788-R) the bone reaches most anteriorly, at d5 distal carena tooth level (Fig 12).

A characteristic shared by *Itasuchus*, *Pepesuchus*, and *Roxochampsa* (not certain for *Caririsuchus*), and which can be consider a synapomorphy for Itasuchidae, is the progressive decrease in teeth size of three posterior teeth subsequent to the hypertrophied d4 (being d7 and d8 very small mainly in *Pepesuchus* and *Roxochampsa*), with the formation of a small diastema between d7–d8 to receives an upper maxillary caniniform tooth (Fig 12).

One of the differences between *Roxochampsa* and *Itasuchus* is the second largest tooth in the mandible (being d4 the hypertrophied in the row) and its placement in the second wave convexity of occlusal edge undulation. In *Roxochampsa* this enlarged tooth is the d12 (a condition shared with *Pepesuchus*) while in *Itasuchus* is the d11 (Fig 12).

**Fig 13:**
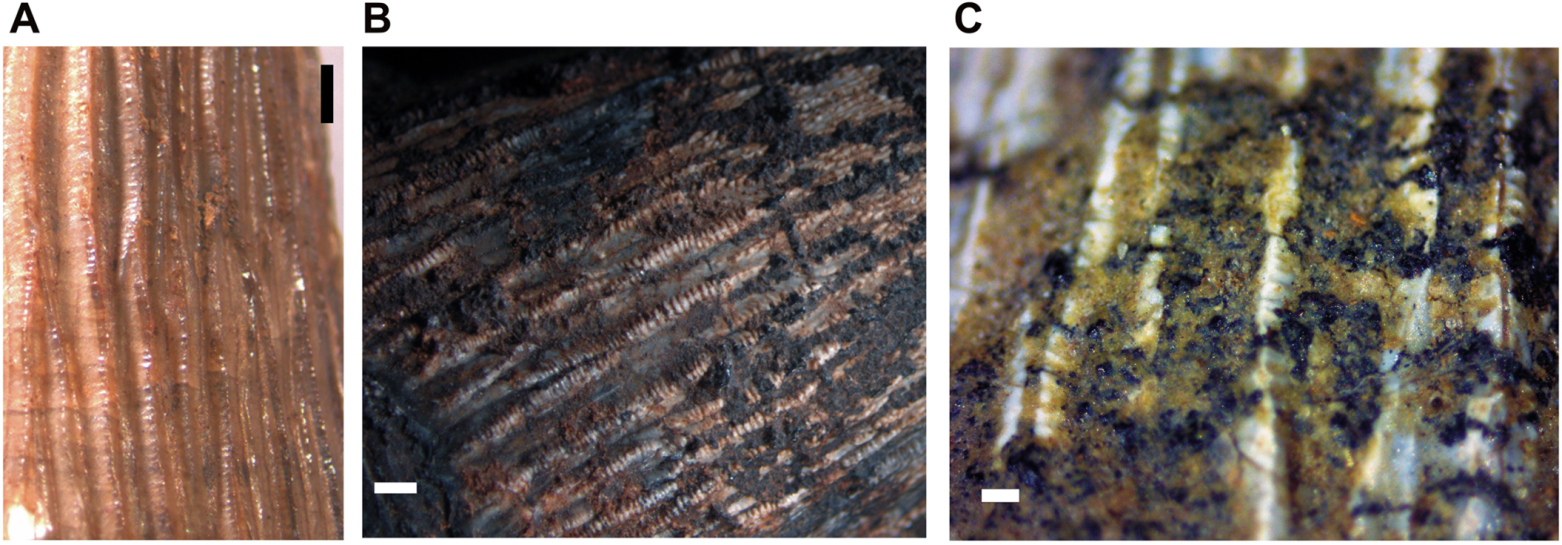
Details of the lingual crown surfaces, showing multicrenulate longitudinal ridges, from *Roxochampsa paulistanus* comb. nov. specimens. A- DGM 259-R; B- d12 of UFRJ-DG 451-R; C- d12 of UFRJ-DG 501-R. Each scale bar = 1 mm.

The tooth morphology of *Roxochampsa paulistanus* is more similar to *Itasuchus* rather than *Pepesuchus* in number and dimension of high relief apicobasal ridges, which are less numerous in *Pepesuchus*, besides more marked and more regular in *Pepesuchus*.The mesial and distal carinae of *Pepesuchus* teeth are smooth but crenulated by false denticles in *Roxochampsa* and *Itasuchus*. Furthermore, the *Roxochampsa* specimens (DGM 259-R, UFRJ-DG 451 and 501-R) have high relief apicobasal longitudinal ridges even crenulated by pseudo-denticles [132] (Fig 13), an anatomical feature shared and previously found only for the marine semiaquatic thalattosuchian *Machimosaurus hugii* von Meyer 1837 (*vide* [51, 132]). However, striking differences in the mandible anatomy between Early jurassic–Late cretacic *Machimosaurus* and Early cretacic *Roxochampsa paulistanus* (e.g. dentary height [higher in *R*. *paulistanus*], occlusal margin profile [more wavy in *R*. *paulistanus*], tooth count [about twenty four to twenty five teeth for *M*. *buffetauti* rather than nineteen teeth for *R*. *paulistanus*], mandibular symphysis extension [reaching the d20 level in *M*. *buffetauti*, while the d9 level for *R*. *paulistanus*]), being reinforced by our cladistic results, allow us to consider a case of parallelism between these two non closely-related and semiaquatic taxa (Fig 12).

We propose the term multicrenulated tooth for a tooth with such crown morphology (i.e. circular to subcircular in section, with main carinae crenulated and formed by false denticles, together with multiple high relief apicobasal ridges crenulated by pseudo-denticles [131], and “scaly-form” apex), fully verified only for *Roxochampsa paulistanus* at moment. *Machimosaurus hugii and M*. *rex* exhibits a very similar tooth pattern to *R*. *paulistanus*, considered here to also have multicrenulated teeth. However, some differences concerning the morphology of the tooth apex in the *Machimosaurus* are notable, such the scaly form were the protuberances are less individualized, compounding an anastomosed pattern due the confluence of the enamal ridges in this area [52, 142].

A remarkable fact refers to the absence of crenulations for the apicobasal ridges of the tooth of DGM 258-R (together with DGM 259-R, were originally referred to “*Goniopholis*” *paulistanus* [7]), with two explanatory hypotheses: (*i*) the teeth DGM 258-R and 259-R does not belong to the same species; (*ii*) multicrenulated teeth are restricted to some portions of the tooth row. Concerning the first hypothesis, Roxo [7] states these two teeth came from different sites, and if they do not belong to the same species, DGM 258-R cannot be distinguished from *Itasuchus* teeth. For the second hypothesis, multicrenulated teeth were identified in rostral and middle region of mandible tooth row of UFRJ-DG 501-R, but many of those teeth are lost, broken or covered by a post depositional manganese crust, which does not allow us to identify crenulated apicobasal ridges for caudal teeth of the series (being DGM 258-R a blunt and comparatively lower and wider than the multicrenulated DGM 259-R, suggesting a mid-caudal tooth). To reinforce the second hypothesis, Price [10] noted that middle and caudal teeth, beyond d11, of *Itasuchus jesuinoi* DGM 434-R also lacks high reliefed apicobasal ridges.

Another dentition difference between itasuchid taxa (not available for *Caririsuchus*) is about “scaly-forms” rugosities found on the tooth apices of UFRJ-DG 451, 501-R and DGM 259-R, which are weakly developed in DGM 258-R but absent for the teeth of *Itasuchus* and *Pepesuchus* materials. These features are common for some neosuchian taxa as goniopholids (e.g., *Anteophthalmosuchus hooleyi* [143]) and teleosaurids [131, 132].

Despite the tooth general similarities among itasuchids (mainly *Roxochampsa paulistanus* and *Itsuchus*) with goniopholidids (e.g. *Goniopholis*, *Sunosuchus*, *Anteophthalmosuchus hooleyi*) and teleosaurids as *Machimosaurus*, anatomical differences in mandible construction, the lack of further compatible elements between them, the improbable meridional distribution for those last, and supported by our cladistic results, we points a mosaic parallelism phenomenon for many distinct tooth characteristics (e.g. different states for carinae, apicobasal ridges, apex) between those not close-related aquatic/semiaquatic forms.

Due to the similarities between the teeth of UFRJ-DG 451-R and 501-R with those originally designated as “*Goniopholis*” *paulistanus* by Roxo [7], mainly DGM 259-R, we consider all these specimens (UFRJ-DG 451, 501-R and DGM 259-R) as belonging to same species, in the new genus *Roxochampsa*.

Regarding DGM 258-R, for now, we identified it as *Roxochampsa paulistanus* The proximal fragment of an isolated right tibia: DGM 225-R, associated with referred teeth by Roxo [7], was collected on a different site, approximately 40 km from the teeth locality (*vide* [33]), and here systematically treated as indeterminate by its scarcity and general morphology, without intraspecific relevant characters.

Concerning the epiphyseal fragment of the right tibia DGM 225-R (Fig 1A), its proximal portion is well expanded with an elliptical articular surface; seemingly there is a smooth elevation as a low and robust crest that extends until diaphysis shaft beginning. This anatomical feature resembles the tibia of *Mahajangasuchus* but differs from some taxa as *Araripesuchus*, *Baurusuchus*, *Simosuchus*, *Yacarerani*, and *Terminonaris*. In addition, DGM 225-R was not found associated with nor not even in same collection site as the teeth DGM 258 and 259-R. Unfortunately, this specimen is currently lost in the CPRM/MCT collection. Therefore, here we cannot consider the referred tibia fragment as belonging to *Roxochampsa paulistanus* or any other known Bauru’s mesoeucrocodylians.

The specimens (UFRJ-DG 451 and 501-R) are fragmentary, but share the same autapomorphies as the lectotype of *Roxochampsa* allowing us to refer them to this genus. Based on the lectotype and the new referred specimens, *Roxochampsa* has at least three characters differing from *Itasuchus*, two of which autapomorphic (see in diagnosis).

### Phylogenetic discussion

Herein, one of the main objects was to assess the phylogenetic relations of *Roxochampsa paulistanus*, a taxon that historically has been neglected in cladistic analyses due to the scarcity of material. With the discovery of new specimens, its inclusion in a phylogenetic analysis can be done.

Overall, the results are not so disparate from which other works suggest, with the main major groups as Crocodylomorpha, Crocodyliformes, Mesoeucrocodylia and Eusuchia in agreement with most of them. However, many less inclusive clades and some taxa are here disposed of in a new phylogenetic framework.

Before the introduction of cladisticsr, *Baurusuchus* and *Sebecus* were grouped in the obsolete Linnean suborder Sebecosuchia [144, 145]. After popularization of cladistic analysis with Benton and Clark [71] and Clark [146], similar oreinirostral and ziphodont taxa (e.g. *Bretesuchus*, *Bergisuchus* and *Pabweshi*) were recovered within Sebecosuchia by some works (e.g. [82, 146–149]). Nevertheless, a new phylogenetic relation for these taxa was proposed by Larsson and Sues [35], that showed *Pabweshi* as a basal taxon close to the group formed by ((*Sebecus* + *Bretesuchus*) (Peirosauridae)), which was called Sebecia; with *Baurusuchus* not sharing a close common ancestor with *Sebecus*. In this hypothesis, Sebecia is supported by three character states related to palatal morphology, being closed related as a sister group of the clade Neosuchia, sharing about ten unambiguous character states for this node. Here, Sebecia is recovered rather than Sebecosuchia, which is composed of: (*Stolokrosuchus* (((*Barreirosuchus* + *Ayllusuchus*) (Itasuchidae)) ((Peirosauridae) (*Mahajangasuchus* + Sebecidae)))). Contrary to Larsson and Sues [35], Sebecia is here found to be the sister group of Notosuchia, a lineage formed by more terrestrialized morphotypes, instead of mostly semiaquatic-aquatic neosuchians.

The clade Neosuchia was originally proposed by Benton and Clark [71], to include the follow clades: Atoposauridae, Goniopholididae, Pholidosauridae, Dyrosauridae, *Bernissartia*, *Shamosuchus*, and eusuchians. The present work recovered similar results regarding the original proposition, but refutes Metasuchia original hypothesis, by the same authors, in which excludes Thalattosuchia from Mesoeucrocodylia (here, Thalattosuchia appears as a sister clade of Dyrosauridae and Pholidosauridae, *contra* Benton and Clark [71] and Andrade *et al*. [36]). The clade Goniopholididae is more related with Eusuchia, as previous analysis (e.g. [150, 151], *contra* [36]).

Recent phylogenetic analysis include some wild card taxa, as Peirosauridae (e.g. [152]), Mahajangasuchidae (e.g. [153]) and *Araripesuchus* (e.g. “alternate” proposal by Benton and Clark [71]) in a sister clade relationships with Neosuchia. The present work did not recover those clades/taxa as being closelyas related to Neosuchia, which could explain the homoplasies between those clades and Neosuchia. One of the main features attributed to support the Neosuchia according to Benton and Clark [71] is the sinusoidal maxilla in lateral view (character 172 from present analysis).However, this character seems homoplastic and recovered as support to the other groups, as the clade Itasuchidae (discussed below).

The taxon Notosuchia was erected by Gasparini in 1971 [154] to group the crocodyliforms with short rostrum (brevirostrines) and reduced tooth count, but also with a hypertrophied tooth thato occluses with the upper jaw where the premaxillae and maxillae contact. Other features for notosuchians includes the presence of an antorbital fenestrae, external nares opening anteriorly, presence or absence of maxilo-palatal fenestrae and orbits markedly lateralized. The clade was formalized in a phylogenetic context by Sereno *et al*. [155] being defined as: “all crocodyliforms more closely related to *Notosuchus terrestris* than to *Crocodylus niloticus*”.

Nowadays Notosuchia is a recurrent taxon within Mesoeucrocodylia with only some divergences regarding its species content (e.g. [31, 36, 155, 156]). This work is congruent with the morphological definition made by Gasparini [154]), and recovered Notosuchia to includes: Uruguaysuchidae, Baurusuchidae, Sphagesauridae and its closely related species (Fig 11); but excludes other traditional clades such as Peirosauridae (e.g. [152], *contra* [120, 156]) and Sebecosuchia and/or Sebecidae (e.g. *contra* [27, 31, 120, 147, 155, 157]). An exception in the clade, considering Gasparini’s original concept, is Baurusuchidae within Notosuchia, once the rostrum gets slightly elongated for them. However, the increase in length is not restricted to skull but also observed along all the body as observed in the most derived species (*e*.*g*. *Stratiotosuchus* and *Baurusuchus*), with basal baurusuchids (*i*.*e*. *Campinasuchus* and *Pissarrachampsa*) being more short snouted and with a general skull morphology more similar tosmall notosuchians. Therefore, despite the general morphological divergence of *Baurusuchus* and *Stratiotosuchus* with the remaining notosuchians, the phylogenetic hypothesis recovered here supports an independent evolution of the Baurusuchidae clade to a hypercarnivorous lifestyle, while remaining notosuchians evolved adaptations for omnivorous or maybe, in some cases, strict herbivory (e.g. [18, 27, 148, 158–161]).

Another clade traditionally recovered within Notosuchia, excluding more basal forms as Uruguaysuchidae and Peirosauridae, is Ziphosuchia, which includes the advanced notosuchians, Sphagesauridae and Sebecosuchia [120, 156]. Carvalho et al. [15] applied a similar use for Ziphosuchia, but with the exclusion of the species here treated as “advanced notosuchians” [31]. However, Ziphosuchia, in the original proposition [147] is a more inclusive taxon that could be interpreted as synonymous with Notosuchia (as pointed by Pol [148]). Inasmuch, the results here presented are in accordance with a more inclusive usage of Ziphosuchia, similar to the proposition made by Andrade et al. [36], including Notosuchia and Sebecia (here redefined), and being sister taxa of Neosuchia.

Finally, the phylogenetic results support the rehabilitation of the clade Itasuchidae [15]. This clade was originally proposed to include just two species: *Malawisuchus* and *Itasuchus*, being sister taxa of Peirosauridae. The present data recovered the heterodont and brevirostrine *Malawisuchus* as a notosuchian (as the majority of phylogenetic studies do) with close affinities with *Pakasuchus*.Here *Itasuchus* is recovered in a polytomy with *Caririsuchus*, *Roxochampsa*, and *Pepesuchus*. Reinforcing the morphologycal discrepancy among *Malawisuchus* and *Itasuchus*, the fact that Itasuchidae is a name referent to “*Itasuchus* like group” [15], and even the fact that such taxon was the first described within the clade, we agree with the maintenance of the name Itasuchidae for this node. The close affinities between *Pepesuchus* and *Itasuchus* were expected due to its morphological similarities regarding the mandible [24].

In a stem based concept, we redefine Itasuchidae as “all species closer to *Itasuchus jesuinoi* than to *Barreirosuchus franciscoi*, *Montealtosuchus arrudacamposi*, *Mahajangasuchus insignis* and *Sebecus icaeorhinus*”. Based on synapomorphies, Itasuchidae here include the species with the follow features: unsculptured region along alveolar margin on the lateral surface of maxilla (char. 98: 0 -> 1); absence of a notch in premaxilla on lateral edge of external nares (char. 113: 1 -> 0); trapezoidal skull roof (char. 170: 0 -> 1); and, in lateral view the ventral edge of maxilla is sinusoidal (char. 172: 0 -> 1).

The present topology brings Itasuchidae as a sister clade of: (*Barreirosuchus* + *Ayllusuchus*), that in turn is the sister of *Stolokrosuchus lapparenti*. There are no rare platyrostral forms evolving within some terrestrial crocodyliforms lineages which were mainly oreinirostral and brevi-mesorostrine forms. *Stolokrosuchus* is a platyrostral tubular and longirostrine taxon from the Early Cretaceous of Niger, South-West Africa, although phylogenetically debated and not having at least seven of the eleven Benton and Clark’s features correlated with longirostry (*i*.*e*. *i-* nasals do not reach the external nares; *ii*- nares are confluent; *iii-* nasals do not reach the premaxillae; *iv*- supratemporal fenestra are larger than orbits; *v*- basioccipital tubera large; *vi*- lateral edge of maxilla straight; *vii*- premaxilla/maxilla contact without indentation [71]) no longer is considered for some works as a basal notosuchian (e.g. [31]). Larsson and Gado [162] originally placed *S*. *lapparenti* in a polytomic Peirosauridae Family (*Peirosaurus* + *Lomasuchus* + *Stolokrosuchus*), but Peirosauridade within Neosuchia. Another unusual species is the platyrostral narrow and non longirostrine *Lorosuchus nodosus*, a basal sebecid form from mid-late Paleocene of North-Western Argentina [157]. Based on it, all referred species of this clade are longirostrine, with the exception of the more platyrostral *Barreirosuchus* that could be a reversion (type specimen with the rostrum is lost). Therefore, Itasuchidae is here proposed as a clade of longirostrine crocodyliforms, probably associated with semi-aquatic habits. The clade and its related species are sister clade of Peirosauridae plus Mahajangasuchidae and Sebecidae.

As genera are a phylogenetic hypothesis, and as *Roxochampsa* was recovered within a polytomy (not consensual, but a polytomy present in all the minimum-length trees) there are two possible ways for designating a genus for the species: 1) refers all species within the polytomy, here treated as Itasuchidae, as members of the genus *Itasuchu*s, which would result in the reallocation of two other species (*Caririsuchus* and *Pepesuchus*) to the genus *Itasuchus*; and, 2) propose a new monoespecific genus to the species avoiding modifications on the generic status of the other species. Therefore, the more reliable based on the present evidence seems to be the second way, designating a new monospecific genus.

In a broad analysis of our cladistic results, a low ensemble Consistency Index (0.304) implies a high number of homoplastic characters states [163]. However, the relatively elevated ensemble Retention Index (0.692), generally indicates that homoplasies behave in a not autapomorphic manner [164], and so are informative to sustain many of less inclusive nodes.

### Diversity of Bauru’s mesoeucrocodylian dentition

Among Bauru Group mesoeucrocodylians, some different kinds of tooth morphology have been described. The first and isolated "*Machimosaurus*-*Goniopholis*"—type teeth (according to Price [33]) were reported from these rocks in mid-forties of the last decade.

In the description of *Baurusuchus pachecoi* by Price [8], a new tooth morphology was described, standing out as it had numerous general similarities with the teeth of non-avian theropod dinosaurs, referred to as “theropodomorph dentition” [165]. Langston [166] considers as “ziphodont crocodiles” those taxa that bear a tooth with the follow morphology: (*i*) acute crown apex; (*ii*) buco-lingually compression; (*iii*) slight distal recurvation; (*iv*) both carinae (mesial and distal) true serrated by isolated and festoon-like denticles, as formed by enamel as dentine. According to Langston [166] the term “ziphodont” (derived from the specific name of “*Crocodylus ziphodon*”, considered as *nomem nudum*) is a state shared by *Baurushuchus* and *Sebecus* (Eocene of Argentina), both taxa considered by some authors as close related in the monophyletic group known as Sebecosuchia (e.g. [31, 80, 120, 147]), and the Paleogene laurasians eusuchians Planocraniidae (including *Boverisuchus* and *Planocrania*, according [166, 167]). The ziphodont dentition is a condition frequently associated with hypercarnivory, in a set of characters adapted to a more terrestrialized than semi-aquatic life habits as presented by extant crocodylians (e.g. [140, 145, 165, 166, 168–170]). Direct evidence of predator-prey interactions among mesoeucrocodylian paleofauna from the Adamantina Formation was recovered through a baurusuchid specimen of *Aplestosuchus sordidus*, which has a sphagesaurid as abdominal content (*vide* [30]).

The dentition of baurusuchids and sebecids are notable with theirt reduced tooth count, with *Pissarrachampsa sera* only having four teeth per maxilla (with sebecids and baurusuchids [except *Stratiotosuchus maxhechti*: *pm*(3)+*m*(5)/*d*(9)], having four premaxillary teeth), and also by an anisodont tooth row, formed by all ziphodont teeth. The general similarities between teeth of ziphodont crocodyliforms and carnivorous dinosaurs led to some mistakes in the interpretations of South America fossil record, with some paleontologists hypothesising the presence of derived mammals during the Upper Cretaceous of Argentina, and the possible presence of dinosaurs during the Eocene of Argentina and Miocene of Venezuela and Peru (*vide* [171], for a better discussion and review on this topic). Despite external morphology, Riff and Kellner [165] discussed some characters that distinguished between baurusuchid and theropod teeth: (*i*) inner pulp cavity is shorter in baurusuchids, while extends at least until mid crown length in theropods, (*ii*) crown surface is ornated by a mesh of transversal and longitudinal lines in baurusuchids, but absent or very faint for theropod teeth. Since *Baurusuchus pachecoi* was named, other baurusuchids taxa from Bauru Group have been described: *B*. *salgadoensis*; *B*. *albertoi*; *Stratiotosuchus maxhechti*; *Pissarrachampsa sera*; *Campinasuchus dinizi*; *Gondwanasuchus scabrosus* and *Aplestosuchus sordidus*. Special consideration is made here concerning to the smallest baurusuchid *Gondwanasuchus scabrosus*, found in Adamantina Formation sediments (the only specimen: UFRJ DG 408-R, has total skull length estimate of 150mm and is consider a subadult [29] that, despite serrated carinae, the dentition does not conform to a general ziphodont pattern. The serrated teeth have a subcircular cross-section but the crown exhibit vertical grooves on it is an outer surface, with the teeth bear five or six deep and wide apicobasal sulci, which converge apically and are interspersed with ridges, conferring a slightly ribbed shape for the crown surface.

Langston [171] also noted some differences between sebecid (reminding that Langston [172] consider *Sebecus* and *Boverisuchus* [*Pristichampsus* as *nomen dubium* [167]] as belong to the same family) and dinosaur carnosaurs teeth, when its discussion about the possibility of the sebecosuchian cosmopolitism. Due its broad distribution among archosaurian lineages, both as in Crurotarsi (Phytosauria, Rauisuchia, and Crocodylomorpha) and Avemetatarsalia (non-avian theropod dinosaurs), and within Crocodylomorpha (e.g. *Hesperosuchus agilis*, *Hsisosuchus*, some peirosaurids, Baurusuchidae, Sebecidae and Planocraniidae), “ziphodont-theropodomorph pattern” dentition is highly homoplastic, that disappears and subsequently reappears along the different subclades of Archosauria generating a number of reversions and parallelism; being so considered as having low phylogenetic signal (e.g. [82, 117, 118, 172–175]).

After the 1980s, new and abundant mesoeucrocodyliform data was recovered from Bauru Group and other deposits of Cretaceous of South America. Some taxa, not immediately closed related to baurusuchids and sebecids, were found to have teeth with true serrated carinae, such as the uruguaysuchid *Araripesuchus wegeneri* (uruguaysuchid from K Inf. of Elrhaz Formation, Gadoufaoua, Niger); and the peirosaurids, discussed below.

Chronologically, the second and intriguing tooth morphotype from Bauru Group was described by Price in 1950, when the description of *Sphagesaurus huenei* [9]. This taxon was based on two isolated teeth first reported by Huene [6] and collected in 1917 from different sites along the railway cut between Presidente Prudente and Santo Anastásio cities [9]. These striking, unusual teeth have both compressed roots and crowns, with the latter unequally labio-lingually compressed, which confers a “tear-shaped” when in section. The crown is conical and covered by heavy enamel coat on the surface of which is coarsely pebbled. Few, prominent irregularly spaced carinae displaced longitudinally on the crown surface. Anterior face of crown bears no keel. Posterior face of crown developed into a very prominent keel bearing a series of small tubercles along its crest [9]. These isolated teeth led Price [9] to consider *Shagesaurus huenei* as a specialized carnivore, which could have had an occlusal framing similar to mammalian carnivores, with carnassial teeth suited to tearing and cutting flesh. Fortunately, abundant cranial and postcranial materials related to *Shagesaurus huenei* were recovered from Bauru Group deposits (e.g. [14, 18, 20, 22, 27, 31, 148]), and a distinct and radical vision fell on these heterodont forms. The oblique and interlocking occlusion like a series of reversed triangles arrangement and the palinal and lateromedial mandible movements, are good indications of a sophisticated oral food processing for these endemic South American sphagesaurids (i.e. *Sphagesaurus*, *Armadillosuchus*, *Caryonosuchus* and *Caipirasuchus*), being more related to omnivorous forms than strictly carnivores (e.g. [18, 50, 117, 148]).

One of the most abundant and best known heterodont notosuchians from Bauru Group is *Mariliasuchus amaral* [99, 176] a taxon that in some works is phylogenetically founded close to *Notosuchus terrestris* (Upper Cretaceous of Neuquén Group, Argentina) the sphagesaurids (e.g. [31, 170, 177, 178] and our results). The dentition of *Mariliasuchus* (tooth formula: *pm*(4)+*m*(5)/*d*(9)), is generally constituted by two incisiviform most anterior teeth which are followed by a big caniniform, being the rest of the upper jaw formed by more blunt and low teeth. The dentary has hypertrophied or caniniform teeth, with the six posterior teeth as blunt as the maxillary teeth. The incisiviform and caniniform exhibits an enamel ornated by some regular apicobasal ridges, conferring a multifaceted aspect for them. However, the blunt teeth are quite different from any other species. These blunt teeth have a peaked apex, with the thick enamel showing much lower and anastomosed apicobasal ridges, which have near the base, one to six longitudinally aligned tubercles, and with the distal serrated margin of the second and third maxillary teeth, predominantely directed posteromedially rather than posteriorly (*vide* [100]). For this general tooth morphology, with tubercles and serrations formed by relative few, but big denticles in comparison to Baurusuchidae and Sebecidae, Andrade and Bertini [118] coined the term Ziphomorph, which according to them can also apply to the non incisiviform-caniniform teeth of *Sphagesaurus*, *Adamantinasuchus*, and *Notosuchus*. According to Andrade and Bertini [118] and Ösi [50] the ziphomorph teeth of *Mariliasuchus*, allied to apical wear facet as indicative of proal jaw movements, it allowed to deal with many and distinct kinds of fibrous alimentary items (e.g. coarse leaves, seeds, pinecones, arthropods and small vertebrates); an omnivorous notosuchian.

Despite Nobre and Carvalho [14] suggesting a carnivorous and/or necrophagous diet for *Adamantinasuchus navae* based on a presumably precise occlusion of the giroversal implanted ziphomorph teeth (slightly labio-lingualy oriented) and the absence of wear facet, Ösi [50] hypothesised a high degree of dietary specialisation for this taxon, without specifying them. The Ösi’s argumentation is based on a (*i*) more complex ziphomorph tooth morphology when compared with *Mariliasuchus*, (*ii*) some wear facets identified by him for the mid region tooth row; (*iii*) a relative and small posteriorly positioned oral cavity [50].

Montefeltro et al. [179] described six isolated teeth from a single locality near Ibirá (Adamantina Formation of Northwestern São Paulo State). These teeth have a complex morphology, they are low, circular in cross-section and show the main cusp and smaller accessory cusps arranged in more than one row, plus a cingulum. In comparisons with another multicuspid notosuchians the authors suggested strong affinities with *Candidodon itapecuruensis* Carvalho and Campos, 1988, an small heterodont notosuchid from Albian, Lower Cretaceous, of Itapecuru Formation (NE Brazil). This species has similar postcaniniform teeth morphology and which had led their proponents to originally classify it as a “triconodont” mammal (*vide* [158]) considering such teeth as belonging to family Candidodontidae [15]. Due to the controversies and systematic problems concerning Candidodontidae, as pointed by Montefeltro et al. [179], they suggest a new stem-based phylogenetic definition for Candidontidae: “…all taxa closer to *Candidodon itapecuruensis* than to *Notosuchus terrestris*, *Uruguaysuchus aznarezi*, *Comahuesuchus brachybuccalis*, *Sphagesaurus huenei*, *Baurusuchus pachecoi*, and C*rocodylus niloticus*”. Ösi [50], pointed out that the diversity of the tooth morphology in the tooth row of *Candidodon* prevents the elucidation of the finer dietary habits of the animal.

Peirosaurids were a well-distributed group of basal mesoeucrocodylians during Cretaceous of the Occidental Gowdwana landmass, founded in deposits of Brazil (*Peirosaurus tormini*, *Uberabasuchus terrificus*, and *Montealtosuchus arrudacamposi*, from Bauru Group); Argentina (*Gasparinisuchus peirosauroides*; *Lomasuchus palpebrosus*; *Bayomenasuchus hemandesi* from Upper K of Neuquen Group, and *Barcinosuchus gradilis* from Lower K of Chubut Group); Morocco (*Hamadasuchus rebouli*, from Lower K of Kem Kem Beds), and with doubts in Niger (*Stolokrosuchus lapparenti* from Upper K of Tegama Group). All Brazilians peirosaurids bear teeth with both carinae serrated by fine true denticles, but different from baurusuchids, the dentary and maxillary teeth (from the middle to the back at maxillary tooth row) are not ziphodont in external morphology. In a broad way, the dentition of *Uberabasuchus* and *Montealtosuchus* (*Peirosaurus* is represented only by a left premaxillary with teeth, from the skull) are mildly heterodont, with the tooth row formed by three basic morphotypes (in according to Prasad and Broin [117]): *(i*) the premaxillary, the first three maxillary and first four dentary teeth (not serrated for *Uberabasuchus* CPPLIP 630) are similar to a ziphodont condition but only slightly recurved and less labio-lingually compressed crown; (*ii*) the middle teeth from the rows are serrated and lanceolate concerning crown shape; (*iii*) with the last teeth serrated, acute but low and blunt. *Stolokrosuchus*, a long slender-snouted crocodyliform is interpreted to prey small, agile, aquatic prey, as fishes (e.g. [44, 180]). Carvalho et al. [15]) was the first to infer the diet for a basal terrestrialized taxon (*i*.*e*., *Uberabasuchus*) from these group, that along with the undulated rostrum occlusal margin, propose a carnivore diet by means of predatorial habit on mid-large prey.

Despite our phylogenetic results about *Pepesuchus deisea* (Itasuchidae) it is was cladistically placed in Sebecia clade (*sensu* [35]) and even pointed as a semi-aquatic peirosauridae by their proponents [24]. The dentition of *Pepesuchus* differs of other peirosaurids, and also *Itasuchus* and *Roxochampsa* in having teeth with acute apcies, bicarinate teeth with smooth unserrated carinae, with striated external surfaces formed by well-marked and some regular spaced longitudinal ridges. The crown morphology of *Pepesuchus* is very similar to some neosuchians, such as the slender-snouted and continental semi-aquatic pholidosaurid *Pholidosaurus purbeckensis*, which was expected to feed almost exclusively on fish [180].

The tooth morphology of moderate heterodont itasuchids, as *Itasuchus* and *Roxochampsa*, were classified as "*Machimosaurus*-*Goniopholis*" complex or type [7, 10, 33]. The teeth of *Itasuchus* and *Roxochampsa* are convergently very similar to neosuchians such as the teleosaurids "*Steneosaurus*" *obtusidens* [181, 182], *Machimosaurus*, (e.g. [51, 52, 132]), pholidosaurid *Pholidosaurus* (e.g. [183]) and goniopholidids *Sunosuchus*, *Goniopholis* and *Anteophthalmosuchus* (e.g. [36, 142, 184]), with main similarities: (*i*) acute non-sharp monocuspid teeth; (*ii*) subcircular in cross-section; (*iii*) crown surface ornamented with low and subparallel apicobasal ridges; (*iv*) and mesial and posterior carinae smooth or crenulated in a false-ziphodont condition [117, 118, 131, 132]. However, the teeth of *Roxochampsa* have some features not found in *Itasuchus* and those mentioned neosuchians. Here, we propose multicrenulated tooth pattern for crown morphology that involves some characteristics, as subparallel and anastomosed apicobasal ridges coarsely crenulated by pseudo-denticles both in buccal and lingual surfaces aside the even crenulated main carinae (character shared with *Machimosaurus hugii* and *M*. *rex* [51, 52, 131]), imbricated hooked-like structures near the top, and a rugous apex.

### Paleobiology and paleoecology inferences

*Roxochampsa paulistanus* (UFRJ-DG 501-R) has a preserved dentary length of 20.7 cm, which at least 13 cm corresponds to the total symphyseal length, 5cm is splenial symphysis and 8 cm is dentary symphysis. Usually, definitions regarding the rostrum length of crocodyliforms are based on the proportion between the rostrum and the basal skull length (from orbits to the posterior margin of skull table [44]). However, either with the absence of the skull to make this comparison, we can propose based on the symphyseal mandibular length, that *Roxochampsa paulistanus* is a meso-longirostrine form. Based on the assumption that for a longirostrine species the mandibular symphysis corresponds to approximately 60% of mandible total length, the mandible of UFRJ-DG 501-R would have be approximately a total mandibular length of 25 cm (estimations based on *Pepesuchus deiseae* due its morphological similarities and completeness [24]), however, the preserved fragment UFRJ-DG 501-R possess about 20 cm (Figs 5 and 11) with all the posterior portion of the mandible is missing, including surangular, angular and articular, and so the total mandibular length for this taxon is probably some bigger than 25 cm. For the body size estimation, researchers usually made linear regressions based on total skull length (e.g. [155]) or the braincase length (e.g. [185]), which is defended as the more conservative method [186].We are not presenting any statistical linear estimations for *Roxochampsa paulistanus* due to the scarcity of available material for a more precise calculus, and for the fact that those estimations are based on extant semiaquatic species, in which the extrapolation of its body skull and size proportions for non-neosuchian fossils are questionable. In this way, based on the total mandibular estimation done here, and based on the body size estimation of *Caririsuchus camposi* [47, 77] we regarded the UFRJ-DG 501-R is a small to medium sized individual with approximately 1–2 m body length (estimations also based on the approximate total length of *Sarcosuchus imperator* proposed by Sereno et al. [154]). The specimen UFRJ-DG 451-R surely comes from a larger animal than UFRJ-DG 501-R, but because of its incompleteness, comparisons for a reliable estimative it is not possible; but inferred by us as a specimen 10–15% larger than UFRJ-DG 501-R.

The teeth are slightly more acute in *Pepesuchus* material and the specimen *Itasuchus sp*. (MUGEO 218-V), than in specimens of *Itasuchus jesuinoi* (DGM 434-R) and in *Roxochampsa* (DGM259-R, UFRJ-DG 451, 501-R). It may be added that the crown shape is known to vary ontogenetically in living species [187]. Lucas and Luke [188] (but also see [189]), presented an ontogenetic development pattern for crocodilian teeth, where the younger specimens present a more acute apex, for both teeth morphotypes, with the posterior ones being lanceolate-like in young individuals, while in the older specimens both teeth morphotypes had more rounded apex. Based on this assumption (also being supported by the tomographic analysis were a wave pattern teeth substitution with the substitution teeth being more rounded at the apex) we can propose a mature age for the specimens known for *Roxochampsa paulistanus*, as well as *Itasuchus jesuinoi* DGM 434-R; being the referred *Itasuchus* specimen MUGEO 218-V inferred to having a more young age compared to DGM 434-R based on its acute apex observed among their preserved rostral-middle teeth. However, to *Pepesuchus*, the similarities of the acute teeth crown shape between holotype MN 7005-V and paratype MCT 1788-R could be more related to a specific characteristic instead of a juvenile age.

*Roxochampsa paulistanus* has peculiar dentition: a highly specialized tooth morphotype (multicrenulated), together with three general morphotypes of teeth (a conical with more acute apex that correspond with the anterior rostral ones, some acute and lanceolate crown teeth for middle row; and the small and more blunt ones that correspond to the teeth from caudal series portion, a variation in the mandible tooth row morphology also observed in *Itasuchus*, *Pepesuchus*, and peirosaurids). Additionally, despite its narrow snout, the presence of blunt teeth at the caudal mandible row, where the mandibular force and resistance are higher, enables the adult specimens to feed on harder food items such as shelled mollusks, crabs, maybe small sized turtles, and/or crush bones of dead animals. Dentition variation in upper and lower jaws, with the presence of caudal tribodont teeth (term used by Buffetaut and Ford [190] for a blunt and rounded tooth) is well observed in extinct and extant caimanins and alligatorines, and also corresponds to a durophagous diet (e.g. [191–195]).

Probably as a semiaquatic species of small to medium size, *Roxochampsa paulistanus* differs from piscivorous species such as the longirostrine *Gavialis* by having a more robust, high and festooned mandible morphology, which could imply on a more generalized food intake than a piscivorous one (see [194]). Langston [196] pointed out that the alternating occlusion of mesorostral eusuchians crocodiles is a specialization for stabbing and crushing the prey, whereas in longirostrine and narrow-snouted forms (e.g. *Gavialis*, *Tomistoma*) the similar occlusion serves as an added function of striking and impaling, especially fishes (primary function would exert by acute and homodont dentition). *Roxochampsa paulistanus* and other itasuchids are narrow-snouted taxa within Sebecia, mesorostral in snout length, have alternate oclusion and moderately heterodont dentition formed by robust teeth; characteristics related to a more generalist feeding habit.

According to some authors, e.g. [52, 130, 131], *Machimosaurus* was traditionally treated as large nearshore durophagous predatorwith teeth adapted for cruching hard prey.But the discovery of denticulated teeth and the apicobasal fully ornamented by pseudo-denticles by *M*. *hugii* and the giant *M*. *rex* indicates that these forms may have had a more varied feeding strategy that also included slicing flesh. The pseudo-denticles increase the surface area of the apical region of the tooth crowns, and could be a way of maximizing friction, and therefore facilitating grip, on wet prey items (such as marine turtles, some of which have embedded *M*. *hugii* teeth and/or bite marks consistent with *M*. *hugii* [51]). These structures could have also have strengthened the enamel [130].

Sedimentological analyzes, anatomy of osteoderms and tooth morphology of the largest thalattosuchian, *Machimosaurus rex*, provide evidences to interpreted it as an ambush predator of shallow and nearshore waters that preyed on both aquatic and terrestrial vertebrates, analogue with modern semi-aquatic crocodilians [52]. In a similar way for *Machimosaurus* [51, 52, 131, 132], the multicrenulated teeth of *Roxochampsa paulistanus*, could refers to a semi-aquatic carnivorous able to handle carcasses, hard shelly preys as turtles, small to medium terrestrial and soft-bodies vertebrates, as well as catching fish.

## Conclusions

The enigmatic taxon “*Goniopholis*” *paulistanus* was reanalyzed in the light of new material collected from the uppermost sequence of the Presidente Prudente Formation in Alfredo Marcondes municipality, Early Maastrichtian, correlated level in the Adamantina Formation first record of Valparaíso and Mirandópolis regions, allowing us to validate the species in a new taxonomic combination *Roxochampsa paulistanus*. Based on the dubious nature of *Goniopholis* occurrences in South America, and the disparity in mandibular morphology between Goniopholididae and this species, the new genus *Roxochampsa* is here proposed.

The new material reveals another tooth morphology present in the Bauru Group, just only verified for *Roxochampsa paulistanus* and the thalattosuchians teleosaurids: *Machimosaurus hugii* and *M*. *rex*, in which the tooth crown exhibits the main carinae and additionally secondary ones (high relief apicobasal ridges) crenulated by pseudo-denticles. However, the morphological tooth similarities among itasuchids with some neosuchians as goniopholids and some teleosaurids, it reveals a pattern of dental convergence within Mesoeucrocodylia, that lacks a phylogenetic signal.

In our cladistic analysis *Roxochampsa paulistanus* was recovered in the node Itasuchidae, being closed related to *Caririsuchus*, *Itasuchus*, and *Pepesuchus*. This clade is nested within Sebecia, which is here founded to be the major sister group to Notosuchia, both composing a more inclusive clade Ziphosuchia. Thus, the neocretacic mesoeucrocodylian fauna for South America was formed by terrestrialized and continental semi-aquatic forms from two distinct clades: notosuchians, well-adapted for terrestrial niches (e.g. Uruguaysuchidae, Sphagesauridae, Baurusuchidae), and sebecians as more generic forms that included numerous reversions to an semiaquatic lifestyle (e.g. itasuchids, *Stolokrosuchus*, *Mahajangasuchus* and *Lorosuchus*). In accordance with previous works, we found many homoplastic characters for Crocodyliformes, which could imply a mosaic evolutionary pattern, heavily influenced by the ancestral crocodyliform *bauplan*.

## Supporting information

S1 File Supporting information. This files contains: Character List, Data Matrix and Autapomorphies and Synapomorphies common to 225 MLT’s.
(DOCX)
