# Supplementary material for: Reassessment of the enigmatic crocodyliform "Goniopholis" paulistanus Roxo, 1936: Historical approach, systematic, and description by new materials
Source: PLoS One. 2018 Aug 1;13(8):e0199984. doi: 10.1371/journal.pone.0199984 (PMC6070184; doi:10.1371/journal.pone.0199984)
Supplement: S1 File — This files contains: Character List, Data Matrix and Autapomorphies and Synapomorphies common to 225 MLT’s. (DOCX) [file pone.0199984.s001.docx]

**SUPPLEMENTAR MATERIAL**

**Reassessment of the enigmatic crocodyliform “*Goniopholis*” *paulistanus* Roxo, 1936: Historical approach, Systematic, and Description by new materials**

André E. Piacentini Pinheiro^1^*, Paulo Victor L. G. C. Pereira^2^, Rafael G. de Souza^3^, Arthur S. Brum^3^, Ricardo T. Lopes^4^, Alessandra Silveira Machado^4^, Lílian P. Bergvist^2^ & Felipe M. Simbras^1*^

^1^ Faculdade de Formação de Professores (FFP), Universidade do Estado do Rio de Janeiro (UERJ), *Campus* São Gonçalo, Rua Dr. Francisco Portela, 1470, Bairro do Patronato, São Gonçalo, Rio de Janeiro, Brazil

^2^ Laboratório de Macrofósseis, Departamento de Geologia (DEGEO), Universidade Federal do Rio de Janeiro (UFRJ), Ilha do Fundão, Avenida Athos da Silveira Ramos s.nº 430, Rio de Janeiro, Brazil

^3^ Laboratório de Sistemática e Tafonomia de Vertebrados Fósseis, Setor de Paleovertebrados, Departamento de Geologia e Paleontologia, Museu Nacional, Universidade Federal do Rio de Janeiro (UFRJ), Quinta da Boa Vista s/n, 20940-040, São Cristóvão, Rio de Janeiro, RJ, Brazil

^4^ Laboratório de Instrumentação Nuclear - COPPE/UFRJ, Ilha do Fundão, Avenida Athos da Silveira Ramos s.nº, Rio de Janeiro, Brazil

***Corresponding authors

E-mail: [paleolones@yahoo.com.br](mailto:paleolones@yahoo.com.br) (FFP / UERJ); [felipe.simbras@gmail.com](mailto:felipe.simbras@gmail.com)

Character list

The character list for the phylogenetic analysis used herein is given below. The character list is a modified version of that from Pol et al. (2014), from this list the follow 25 characters were removed: **8**, **12**, **35**, **44**, **50**, **52**, **81**, **87**, **89**, **110**, **172**, **196**, **202**, **263**, **265**, **274**, **276**, **277**, **281**, **289**, **333**, **364**, **372**, **388** and **390**. Below, follow the individual justifications for such deletions:

**Char. 08** (Clark, 1994: char. 8): state “0” is autapomorphic for *Dibothrosuchus* after excluding 24 taxa; [criterion 1]

**Char. 12** (Clark, 1994: char. 12): this character is ambiguous as the states are not clearly distinguishable, once the lacrimal becomes an irregular bone, the anterior edge seems fallacious (i.e. if the anterior and medial edges become indistinguishable); [criterion 3]

**Char. 35** (Clark, 1994: char. 35): the character mixes two independent variables, form and ornamentation; [criterion 2]

**Char. 44** (Clark, 1994: char. 44): the character 44 is biological dependent with character 43; [criterion 2]

**Char. 50** (Clark, 1994: char. 50): state “1” is autapomorphic for *Protosuchus* after excluding 24 taxa; [criterion 1]

**Char. 52** (Clark, 1994: char. 52): state “0” is autapomorphic for *Dibothrosuchus* after excluding 24 taxa; [criterion 1]

**Char. 81** (Clark, 1994: char. 81): problem with the homology statement, the dentary teeth posterior to tooth opposite premaxilla-maxilla contact cannot be assured to be the same for different species; [criterion 2]

**Char. 87** (Clark, 1994: char. 87): state “1” for all taxa, without variation [criterion 1]

**Char. 89** (Clark, 1994: char. 89): state “1” is autapomorphic for *Uruguaysuchus* after excluding 24 taxa; [criterion 1]

**Char. 110** (Wu and Sues, 1996: char. 40): state “0” is autapomorphic for *Cricosaurus* after excluding 24 taxa; [criterion 1]

**Char. 172** (Ortega *et al*., 2000: char. 70): state “0” is autapomorphic for *Gracilisuchus* after excluding 24 taxa; [criterion 1]

**Char. 196** (Wu *et al*., 1997: char. 103): the character 196 is biological dependent with character 36; [criterion 2]

**Char. 202** (Wu *et al*., 1997: char. 112): the character 202 is biological dependent with character 280; [criterion 2]

**Char. 263** (Brochu, 1997: char. 84): character states recognition is ambiguous due the preservational artefacts in fossil specimens [criterion 3]

**Char. 265** (Brochu, 1997: char. 92): the character 265 is biological dependent with character 254; [criterion 2]

**Char. 274** (Pol *et al*., 2009: char. 274): state “0” is autapomorphic for *Shamosuchus* after excluding 24 taxa; [criterion 1]

**Char. 276** (Pol *et al*., 2009: char. 276): state “0” for all taxa, without variation [criterion 1]

**Char. 277** (Pol *et al*., 2009: char. 277): state “1” is autapomorphic for *Shamosuchus* after excluding 24 taxa; [criterion 1]

**Char. 281** (Pol *et al*., 2009: char. 281): state “1” is autapomorphic for *Shamosuchus* after excluding 24 taxa; [criterion 1]

**Char. 289** (Pol and Powell, 2011: char. 289): state “1” is autapomorphic for *Sebecus querejazus* after excluding 24 taxa; [criterion 1]

**Char. 333** (Pol *et al*., 2012: char. 333): state “1” is autapomorphic for *Sebecus icaeorhinus* after excluding 24 taxa; [criterion 1]

**Char. 364** (Pol *et al*., 2014: char. 264): problem with the homology statement, the parallel dentary symphysis could be absent in some species and there is no guarantee that the comparable regions are homologous; [criterion 2]

**Char. 372** (Pol *et al*., 2014: char. 372): the character is ambiguous because the states are not clearly distinguishable (e.g., poorly X well developed), also it mixes other non-biological dependent feature (i.e., tuberosity X sulcus); [criterion 3]

**Char. 388** (Pol *et al*., 2014: char. 388): problem with the homology statement, the character does not specify the nature of the serration and consequently if they possess true or false denticles (true denticles are formed by enamel and dentine while false ziphodonty is formed just by enamel crenulations); [criterion 2]

**Char. 390** (modified from Andrade and Bertini, 2008a: char. 149 and O´Connor *et al*., 2010: char. 233): incomplete character writing. [criterion 3]

Below, the list of active characters. Modified characters are indicate by asterisk (*).

**Character 1** (Pol *et. al*., 2014 char. 1; modified from Clark, 1994: char. 1): + External surface of dorsal cranial bones: smooth (0), slightly grooved (1) and heavily ornamented with deep pits and grooves (2).

**Character 2** (Pol *et. al*., 2014 char. 2; modified from Clark, 1994: char. 2): Skull expansion at orbits: gradual (0), or abrupt (1).

**Character 3** (Pol *et. al*., 2014 char. 3; modified from Clark, 1994: char. 3): + Rostrum proportions: narrow oreinirostral (0), broad oreinirostral (1), nearly tubular (2), or platyrostral (3).

**Character 4** (Pol *et. al*., 2014 char. 4, Clark, 1994: char. 4): Premaxilla participation in internarial bar: forming at least the ventral half (0), or with little participation (1).

**Character 5** (Pol *et. al*., 2014 char. 5; Clark, 1994: char. 5) Premaxilla anterior to nares: narrow (0), or broad (1).

**Character 6** (Pol *et. al*., 2014 char. 6; modified from Clark, 1994: char. 6): + External nares facing anterolaterally or anteriorly (0), dorsally not separated by premaxillary bar from anterior edge of rostrum (1), or dorsally separated by premaxillary bar (2).

**Character 7** (Pol *et. al*., 2014 char. 7; Clark, 1994: char. 7): Palatal parts of premaxillae: do not meet posterior to incisive foramen (0), or meet posteriorly along contact with maxillae (1).

**Character 8** (Pol *et. al*., 2014 char. 9; modified from Clark, 1994: char. 9): Ventrally opened notch on ventral edge of rostrum at premaxilla-maxilla contact: absent (0), present as a notch (1), or present as a large notch (2), or present as a notch that is closed ventrally (or largely constrained at its ventral edge) (3).

**Character 09** (Pol *et. al*., 2014 char. 10; modified from Clark, 1994: char. 10): + Posterior palatal branches of maxillae anterior to palatines: do not meet (0), or meet extensively but posterior-most parts fail to meet (1), or meet entirely (2).

**Character 10** (Pol *et. al*., 2014 char. 11; Clark, 1994: char. 11): Nasal contacts lacrimal (0), or does not contact (1).

**Character 11** (Pol *et. al*., 2014 char. 13; Clark, 1994: char. 13): Nasal contribution to narial border: yes (0), or no (1).

**Character 12** (Pol *et. al*., 2014 char. 14; Clark, 1994: char. 14): Nasal-premaxilla contact: present (0), or absent (1).

**Character 13** (Pol *et. al*., 2014 char. 15; modified from Clark, 1994: char. 15): Descending process of prefrontal: does not contact palate (0), or contacts palate (1).

**Character 14** (Pol *et. al*., 2014 char. 16; Clark, 1994: char. 16): Postorbital-jugal contact: postorbital anterior to jugal (0), or postorbital medial to jugal (1), or postorbital lateral to jugal (2).

***Character 15** (**mod.** Pol *et. al*., 2014 char. 17; and Clark, 1994: char. 17): Anterior part of the jugal with respect to posterior part: as broad (0), or twice as broad (1), less broad (2).

**Character 16** (Pol *et. al*., 2014 char. 18; Clark, 1994: char. 18): Jugal bar beneath infratemporal fenestra: flattened (0), or rod-shaped (1).

**Character 17** (Pol *et. al*., 2014 char. 19; Clark, 1994: char. 19): Quadratojugal dorsal process: narrow, contacting only a small part of postorbital (0), or broad, extensively contacting the postorbital (1).

**Character 18** (Pol *et. al*., 2014 char. 20; Clark, 1994: char. 20): Frontal width between orbits: narrow, as broad as nasals (0), or broad, twice as broad as nasals (1).

**Character 19** (Pol *et. al*., 2014 char. 21; Clark, 1994: char. 21): Frontals: paired (0), unpaired (1).

**Character 20** (Pol *et. al*., 2014 char. 22; Clark, 1994: char. 22): Dorsal surface of frontal and parietal: flat (0), or with midline ridge (1).

**Character 21** (Pol *et. al*., 2014 char. 23; modified from Clark, 1994: char. 23 by Buckley and Brochu, 1999: char. 81):+ Parieto-postorbital suture: absent from dorsal surface of skull roof and supratemporal fossa (0), absent from dorsal surface of skull roof but broadly present within supratemporal fossa (1), or present within supratemporal fossa and on dorsal surface of skull roof (2).

**Character 22** (Pol *et. al*., 2014 char. 24; Clark, 1994: char. 24): Supratemporal roof dorsal surface: complex (0), or dorsally flat “skull table” developed, with postorbital and squamosal with flat shelves extending laterally beyond quadrate contact (1).

**Character 23** (Pol *et. al*., 2014 char. 25; modified from Clark, 1994: char. 25) Postorbital bar: sculpted (if skull sculpted) (0), or unsculpted (1).

**Character 24** (Pol *et. al*., 2014 char. 26; modified from Clark, 1994: char. 26): Postorbital bar: transversely flattened (0), or cylindrical (1).

**Character 25** (Pol *et. al*., 2014 char. 27; Clark, 1994: char. 27): Vascular opening in dorsal surface of postorbital bar: absent (0), or present (1).

**Character 26** (Pol *et. al*., 2014 char. 28; modified from Clark, 1994: char. 28): Postorbital anterolateral process: absent or poorly developed (0), or well developed, long, and acute (1).

**Character 27** (Pol *et. al*., 2014 char. 29; Clark, 1994: char. 29): Dorsal part of the postorbital: with anterior and lateral edges only (0), or with anterolaterally facing edge (1).

**Character 28** (Pol *et. al*., 2014 char. 30; Clark, 1994: char. 30): Dorsal end of the postorbital bar broadens dorsally, continuous with dorsal part of postorbital (0), or dorsal part of the postorbital bar constricted, distinct from the dorsal part of the postorbital (1).

**Character 29** (Pol *et. al*., 2014 char. 31; Clark, 1994: char. 31): Bar between orbit and supratemporal fossa broad and solid, with broadly sculpted dorsal surface if sculpture present (0), or bar narrow, sculpting restricted to anterior surface (1).

**Character 30** (Pol *et. al*., 2014 char. 32; modified from Clark, 1994: char. 32): Parietal: with broad occipital portion (0), or without broad occipital portion (1).

***Character 31** (**mod.** Pol *et. al*., 2014 char. 33; and Clark, 1994: char. 33) Parietal: with broad sculpted region separating fossae (0), or narrow, often with a sagittal crest between supratemporal fossae (1).

**Character 32** (Pol *et. al*., 2014 char. 34; Clark, 1994: char. 34): Postparietal (dermosupraoccipital): a distinct element (0), or not distinct (fused with parietal?) (1).

**Character 33** (Pol *et. al*., 2014 char. 36; modified from Clark, 1994: char. 36 and Riff, 2007: char. 36): Posterolateral process of squamosal: poorly developed and projected horizontally at the same level of the skull (0), elongated, thin, and posteriorly directed, not ventrally deflected (1), or elongated, posterolaterally directed, and ventrally deflected (2), or elongated and ventrally directed forming an angle of approximately 90 degrees with the skull roof (3), or posterodorsally deflected (4).

**Character 34** (Pol *et. al*., 2014 char. 37; Clark, 1994: char. 37): + Palatines: do not meet on palate below the narial passage (0), form palatal shelves that do not meet (1), or meet ventrally to the narial passage, forming part of secondary palate (2).

**Character 35** (Pol *et. al*., 2014 char. 38; Clark, 1994: char. 38): Pterygoid: restricted to palate and suspensorium, joints with quadrate and basisphenoid overlapping (0), or pterygoid extends dorsally to contact laterosphenoid and form ventrolateral edge of the trigeminal foramen, strongly sutured to quadrate and laterosphenoid (1).

**Character 36** (Pol *et. al*., 2014 char. 39; modified from Clark, 1994: char. 39): Choanal opening: continuous with pterygoid ventral surface except for anterior and anterolateral borders (0), or opens into palate through a deep midline depression (choanal groove) (1).

**Character 37** (Pol *et. al*., 2014 char. 40; Clark, 1994: char. 40): Palatal surface of pterygoids: smooth (0), or sculpted (1).

**Character 38** (Pol *et. al*., 2014 char. 41; Clark, 1994: char. 41): Pterygoids posterior to choanae: separated (0), or fused (1).

**Character 39** (Pol *et. al*., 2014 char. 42; modified from Clark, 1994: char. 42 and from Ortega et al., 2000: char. 139): Choanal opening size: moderately broad or narrow, equal or less than 30% the width between the lateral margins of the pterygoid flanges (0), or extremely broad approximately 50% the width between the lateral margins of the pterygoid flanges (1).

**Character 40** (Pol *et. al*., 2014 char. 43; modified from Clark, 1994: char. 43): + Primary pterygoidean palate: forms posterior half of the choanal opening (0), or forms posterior, lateral and part of the anterior margin of the choana (1), or completely enclose choana (2).

**Character 41** (Pol *et. al*., 2014 char. 45; Clark, 1994: char. 45): + Quadrate: without fenestrae (0), with single fenestrae (1), or with three or more fenestrae on dorsal and posteromedial surfaces (2).

**Character 42** (Pol *et. al*., 2014 char. 46; Clark, 1994: char. 46): Posterior edge of quadrate: broad medial to tympanum, gently concave (0), or posterior edge narrow dorsal to otoccipital contact, strongly concave (1).

**Character 43** (Pol *et. al*., 2014 char. 47; Clark, 1994: char. 47): Dorsal, primary head of quadrate articulates with: squamosal, otoccipital, and prootic (0), or with prootic and laterosphenoid (1).

**Character 44** (Pol *et. al*., 2014 char. 48; Clark, 1994: char. 48): Ventrolateral contact of otoccipital with quadrate: very narrow (0), or broad (1).

**Character 45** (Pol *et. al*., 2014 char. 49; Modified from Clark, 1994: char. 49): + Quadrate, squamosal, and otoccipital: do not meet to enclose cranioquadrate passage (0), enclose passage near lateral edge of skull (1), or meet lateral to the passage with otoccipital contacting the quadrate lateral to the posterior opening of the passage (2).

**Character 46** (Pol *et. al*., 2014 char. 51; Clark, 1994: char. 51): Ventromedial part of quadrate: does not contact otoccipital (0), or contacts otoccipital to enclose carotid artery and form passage for cranial nerves IX--XI (1).

**Character 47** (Pol *et. al*., 2014 char. 53; Clark, 1994: char. 53): Basisphenoid rostrum (cultriform process): slender (0), or dorsoventrally expanded (1).

**Character 48** (Pol *et. al*., 2014 char. 54; Clark, 1994: char. 54): Basipterygoid process: prominent, forming movable joint with pterygoid (0), or basipterygoid process small or absent, with basisphenoid joint suturally closed (1).

**Character 49** (Pol *et. al*., 2014 char. 55; modified from Clark, 1994: char. 55 by Ortega et al., 2000: char. 68): Basisphenoid ventral surface: shorter than the basioccipital (0), or wide and similar to, or longer in length than basioccipital (1).

**Character 50** (Pol *et. al*., 2014 char. 56; modified from Clark, 1994: char. 56): Basisphenoid: exposed on ventral surface of braincase (0), or hidden from ventral surface by pterygoid flanges that extend posteriorly up to the level of the basioccipital-basisphenoid suture (1).

**Character 51** (Pol *et. al*., 2014 char. 57; Clark, 1994: char. 57): Basioccipital: without well-developed bilateral tuberosities (0), or with large pendulous tubera (1).

**Character 52** (Pol *et. al*., 2014 char. 58; Clark, 1994: char. 58): Otoccipital: without laterally concave descending flange ventral to subcapsular process (0), or with flange (1).

**Character 53** (Pol *et. al*., 2014 char. 59; Clark, 1994: char. 59): Cranial nerves IX--XI: pass through common large foramen vagi in otoccipital (0), or cranial nerve IX passes medial to nerves X and XI in separate passage (1).

**Character 54** (Pol *et. al*., 2014 char. 60; Clark, 1994: char. 60): Otoccipital: without large ventrolateral part ventral to paroccipital process (0), or with large ventrolateral part (1).

**Character 55** (Pol *et. al*., 2014 char. 61; Clark, 1994: char. 61): Crista interfenestralis between fenestrae pseudorotunda and ovalis nearly vertical (0), or horizontal (1).

**Character 56** (Pol *et. al*., 2014 char. 62; Clark, 1994: char. 62): Supraoccipital: forms dorsal edge of the foramen magnum (0), or otoccipitals broadly meet dorsal to the foramen magnum, separating supraoccipital from foramen (1).

**Character 57** (Pol *et. al*., 2014 char. 63; Clark, 1994: char. 63): Mastoid antrum: does not extend into supraoccipital (0), or extends through transverse canal in supraoccipital to connect middle ear regions (1).

**Character 58** (Pol *et. al*., 2014 char. 64; Clark, 1994: char. 64): Posterior surface of supraoccipital: nearly flat (0), or with bilateral posterior prominences (1).

**Character 59** (Pol *et. al*., 2014 char. 65; modified from Clark, 1994: char. 65): + Palpebrals: absent (0), or one small palpebral present in orbit (1), or one large palpebral (2), or two large palpebrals (3).

**Character 60** (Pol *et. al*., 2014 char. 66; Clark, 1994: char. 66): External nares: divided by a septum (0), or confluent (1).

**Character 61** (Pol *et. al*., 2014 char. 67; Modified from Clark, 1994: char. 67): + Antorbital fenestra as large as orbit (0) or less than half the diameter of the orbit (1) or absent (2).

**Character 62** (Pol *et. al*., 2014 char. 68; modified from Clark, 1994: char. 68 by Ortega et al., 2000: char. 41): Supratemporal fenestrae extension: relatively large, covering most of surface of skull roof (0), or relatively short, fenestrae surrounded by a flat and extended skull roof (1).

**Character 63** (Pol *et. al*., 2014 char. 69; modified from Clark, 1994: char. 69): + Choanal groove: undivided (0), partially septated, with parts of the septum located dorsal to the lateral choanal margins (1), or completely septated, with septum leveled with the lateral margins of the choana along its entire length (2).

**Character 64** (Pol *et. al*., 2014 char. 70; Clark, 1994: char. 70): Dentary: extends posteriorly beneath mandibular fenestra (0), or does not extend beneath fenestra (1).

**Character 65** (Pol *et. al*., 2014 char. 71; modified from Clark, 1994: char. 71): + Lateral flange of retroarticular process: straight and directed ventrally forming an angle of approximately 90 degrees with the longitudinal axis of the mandibular ramus (0), directed posteroventrally or posteriorly, with posterior end slightly upturned (1), directed posteroventrally or posteriorly, with posterior end markedly recurved dorsally (2).

**Character 66** (Pol *et. al*., 2014 char. 72; Clark, 1994: char. 72): Prearticular: present (0), or absent (1).

**Character 67** (Pol *et. al*., 2014 char. 73; modified from Clark, 1994: char. 73): + Articular without medial process (0), with short process not contacting braincase (1), or with process articulating with otoccipital and basisphenoid (2).

**Character 68** (Pol *et. al*., 2014 char. 74; Clark, 1994: char. 74): Dorsal edge of surangular: flat (0), or arched dorsally (1).

**Character 69** (Pol *et. al*., 2014 char. 75; Clark, 1994: char. 75): Mandibular fenestra: present (0), or absent (1).

**Character 70** (Pol *et. al*., 2014 char. 76; Clark, 1994: char. 76): Insertion area for *M. pterygoideous posterior*: does not extend onto lateral surface of angular (0), or extends onto lateral surface of angular (1).

**Character 71** (Pol *et. al*., 2014 char. 77; modified from Clark, 1994: char. 77 and Brochu, 1999: char. 43): + Splenial involvement in symphysis in ventral view: not involved (0), involved slightly in symphysis forming up to 20% symphyseal length (1), or forming close to 30% of the symphyseal length (2), or extensively involved forming up to 50% of the symphyseal length and occupying more than the length of five alveoli (3).

***Character 72** (**mod.** Pol *et. al*., 2014 char. 78; Clark, 1994: char. 78): Posterior premaxillary teeth: similar in size to anterior teeth (0), or hypertrophied (1); or smaller than anterior teeth (2).

**Character 73** (Pol *et. al*., 2014 char. 79; modified from Clark, 1994: char. 79): + Enlarged conical maxillary teeth: absent, no tooth size variation (0), one enlarged tooth (or enlarged wave of teeth) (1), or enlarged maxillary teeth curved in two waves (festooned) (2).

**Character 74** (Pol *et. al*., 2014 char. 80; Clark, 1994: char. 80): Anterior dentary teeth opposite premaxilla-maxilla contact: no more than twice the length of other dentary teeth (0), or more than twice the length (1).

***Character 75** (**mod**. Pol *et. al*., 2014 char. 82; modified from Clark, 1994: char. 82 by Ortega et al., 2000: char. 120): Anterior and posterior scapular edges: symmetrical in lateral view (0), anterior edge more strongly concave than posterior edge (1).

**Character 76** (Pol *et. al*., 2014 char. 83; modified from Clark, 1994: char. 83 by Ortega et al., 2000: char. 121): Coracoid length: up to two-thirds of the scapular length (0), or subequal in length to scapula (1).

**Character 77** (Pol *et. al*., 2014 char. 84; Clark, 1994: char. 84): Anterior process of ilium: similar in length to posterior process (0), or one-quarter or less of the length of the posterior process (1).

***Character 78** (**mod**. Pol *et. al*., 2014 char. 85; and Clark, 1994: char. 85): Pubis: rodlike without expanded distal end (0), slightly expanded distal end (1), or well expanded or "fan-like" distal end (2).

**Character 79** (Pol *et. al*., 2014 char. 86; Clark, 1994: char. 86): + Pubis: forms anterior half of ventral edge of acetabulum (0), or pubis contacting the ilium but partially excluded from the acetabulum by the anterior process of the ischium (1), or pubis completely excluded from the acetabulum by the anterior process of the ischium (2).

**Character 80** (Pol *et. al*., 2014 char. 88; Clark, 1994: char. 88): Fifth pedal digit: with phalanges (0), or without phalanges (1).

**Character 81** (Pol *et. al*., 2014 char. 90; modified from Clark, 1994: char. 90): + Cervical neural spines: all anteroposteriorly large (0), only posterior ones rodlike (1), or all spines rodlike (2).

**Character 82** (Pol *et. al*., 2014 char. 91; modified from Clark, 1994: char. 91 by Buscalioni and Sanz, 1988: char. 37 and by Brochu, 1997a: char. 7): + Hypapophyses in cervicodorsal vertebrae: absent (0),present only in cervical vertebrae (1), present in cervical and the first two dorsal vertebrae (2), present at least up to the third dorsal vertebra (3), or up to the fourth dorsal vertebrae (4).

**Character 83** (Pol *et. al*., 2014 char. 92; Clark, 1994: char. 92): Cervical vertebrae: amphicoelous or amphyplatian (0), or procoelous (1).

**Character 84** (Pol *et. al*., 2014 char. 93; Clark, 1994: char. 93): Trunk vertebrae: amphicoelous or amphyplatian (0), or procoelous (1).

**Character** **85** (Pol *et. al*., 2014 char. 94; Modified from Clark, 1994: char. 94): First caudal vertebrae: amphicoelous or amphyplatian (0), biconvex (1), or opisthocoelous (2), or procoelous (3).

**Character** **86** (Pol *et. al*., 2014 char. 95; Clark, 1994: char. 95): Dorsal osteoderms: rounded or ovate (0), or rectangular, broader than long (1), or square (2), or rectangular, longer than broad (3).

**Character** **87** (Pol *et. al*., 2014 char. 96; modified from Clark, 1994: char. 96, and Brochu, 1997a: char. 40): + Dorsal osteoderms: without articular anterior process (0), with a discrete convexity on anterior margin (1), or with a well-developed process located anterolaterally in dorsal parasagittal osteoderms (2).

**Character** **88** (Pol *et. al*., 2014 char. 97; modified from Clark, 1994: char. 97 by Ortega et al., 2000: chars. 107 and 108): + Rows of dorsal osteoderms: two parallel rows (0), more than two (1), or more than four with accessory ranges of osteoderms (*sensu* Frey, 1988) (2).

**Character** **89** (Pol *et. al*., 2014 char. 98; Modified from Clark, 1994: char. 98): Osteoderms: some or all imbricated (0), or sutured to one another (1), or not in contact (2).

**Character** **90** (Pol *et. al*., 2014 char. 99; Pol *et. al*., 2014 char. 99; Clark, 1994: char. 99): Tail osteoderms: dorsal only (0), or completely surrounded by osteoderms (1).

**Character** **91** (Pol *et. al*., 2014 char. 100; Clark, 1994: char. 100): Trunk osteoderms: absent from ventral part of the trunk (0), or present (1).

**Character** **92** (Pol *et. al*., 2014 char. 101; Clark, 1994: char. 101): Osteoderms: with longitudinal keels on dorsal surfaces (0), or without longitudinal keels (1).

**Character** **93** (Pol *et. al*., 2014 char. 102; Wu and Sues, 1996: char. 14): Jugal: participating in margin of antorbital fossa (0), or separated from it (1).

***Character** **94** (**mod**. from Pol *et. al*., 2014 char. 103; and Wu and Sues, 1996: char. 17): Mandibular symphysis in lateral view: deep or high (0), shallow or low (1).

**Character** **95** (Pol *et. al*., 2014 char. 104; modified from Wu and Sues, 1996: char. 23): Articular facet for quadrate condyle: wider than broad (0), or elongated, equal to or more than twice the length of the quadrate condyles (1).

**Character** **96** (Pol *et. al*., 2014 char. 105; modified from Wu and Sues, 1996: char. 24 and Wu *et al*., 1997: char. 124): + Jaw joint: placed at level with basioccipital condyle (0), below basioccipital condyle about above level of lower toothrow (1), or below level of toothrow (2).

**Character** **97** (Pol *et. al*., 2014 char. 106; modified from Wu and Sues, 1996: char. 27 and Ortega *et al*., 2000: char. 133): Premaxillary teeth: five or more (0), four (1), three (2), or two (3).

**Character** **98** (Pol *et. al*., 2014 char. 107; modified from Wu and Sues, 1996: char. 29): Unsculptured region along alveolar margin on lateral surface of maxilla: absent (0), or present (1).

**Character** **99** (Pol *et. al*., 2014 char. 108; Wu and Sues, 1996: char. 30): Maxilla: with eight or more teeth (0), seven (1), six (2), five (3), or four teeth (4).

**Character** **100** (Pol *et. al*., 2014 char. 109; Wu and Sues, 1996: char. 33): Coracoid: without posteromedial or ventromedial process (0), with elongate posteromedial process (1), or distally expanded ventromedial process (2).

***Character** **101** (**mod**. from Pol *et. al*., 2014 char. 111; and Gomani, 1997: char. 4): Prefrontals anterior to orbits: elongated, oriented parallel to anteroposterior axis of the skull (0), or short and broad, oriented posteromedially-anterolaterally (1), or short and broad, oriented posterolaterally-anteromedially (2).

**Character 102** (Pol *et. al*., 2014 char. 112; modified from Gomani, 1997: char. 32): Basioccipital and ventral part of otoccipital: facing posteriorly (0), or posteroventrally (1).

**Character 103** (Pol *et. al*., 2014 char. 113; Buscalioni and Sanz, 1988: char. 35): Vertebral centra: cylindrical (0), or spool shaped (1).

**Character 104** (Pol *et. al*., 2014 char. 114; modified from Buscalioni and Sanz, 1988: char. 39): Transverse process of posterior dorsal vertebrae dorsoventrally low and laminar (0), or dorsoventrally high (1).

**Character 105** (Pol *et. al*., 2014 char. 115; Buscalioni and Sanz, 1988: char. 44): Number of sacral vertebrae: two (0), or more than two (1).

**Character 106** (Pol *et. al*., 2014 char. 116; modified from Buscalioni and Sanz, 1988: char. 49): + Development and orientation of the rugose surface for the insertion of the M. iliotibialis that forms the supracetabular crest: lateromedially narrow and facing dorsally or slightly laterodorsally (0), lateromedially broad, forming a wide and markedly rugose attachment surface facing laterodorsally (1), or lateromedially broad and rugose that is highly deflected laterally forming a remarkably deep acetabulum (2).

**Character 107** (Pol *et. al*., 2014 char. 117; Buscalioni and Sanz, 1988: char. 54): Proximal end of radiale expanded symmetrically, similarly to the distal end (0), or more expanded proximolaterallythan proximomedially(1).

**Character 108** (Pol *et. al*., 2014 char. 118; modified from Pol and Gasparini, 2009: char. 118): Lateral surface of the anterior region of surangular and posterior region of dentary: without a longitudinal depression (0), or with a deep, well-defined longitudinal groove (1).

**Character 109** (Pol *et. al*., 2014 char. 119; modified from Ortega *et al*., 1996: char. 9): Ventral exposure of splenials along mandibular rami, posterior to the symphysis: absent (0), or present (1).

**Character 110** (Pol *et. al*., 2014 char. 120; modified from Ortega *et al*., 1996: char. 11, Ortega et al., 2000: char. 100, Andrade and Bertini 2008a: char. 132, and Turner and Sertich, 2010: char. 120): Tooth margins in posterior region of the toothrow: with denticulate carinae formed by homogeneous and symmetrical denticles with a sharp cutting edge (0), or without carinae or with smooth or crenulated carinae (1), or with tubercular, rounded denticles (anisomorph sensu Andrade and Bertini, 2008b) (2).

**Character 111** (Pol *et. al*., 2014 char. 121; modified from Pol, 1999a: char. 133 and Ortega *et al*., 2000: char. 145): Lateral surface of anterior process of jugal: flat or convex (0), or bearing a longitudinal ridge or shelf running along its lateral surface and triangular depression underneath it (1).

**Character** **112** (Pol *et. al*., 2014 char. 122; Pol, 1999a: char. 134): Jugal: does not exceed the anterior margin of orbit (0), or exceeds margin (1).

**Character 113** (Pol *et. al*., 2014 char. 123; Pol, 1999a: char. 135): Notch in premaxilla on lateral edge of external nares: absent (0), or present on the dorsal half of the external nares lateral margin (1).

**Character 114** (Pol *et. al*., 2014 char. 124; Pol, 1999a: char. 136): Dorsal border of external nares: formed mostly by the nasals (0), or by both the nasals and premaxilla (1).

**Character 115** (Pol *et. al*., 2014 char. 125; Pol, 1999a: char. 138): Posterodorsal process of premaxilla: absent (0), or present extending posteriorly wedging between maxilla and nasals (1).

**Character** **116** (Pol *et. al*., 2014 char. 126; Pol, 1999a: char. 139 and Ortega et al., 2000: char. 9): + Premaxilla-maxilla suture in palatal view, medial to alveolar region: anteromedially directed (0), sinusoidal, posteromedially directed on its lateral half and anteromedially directed along its medial region (1), or posteromedially directed (2).

**Character 117** (Pol *et. al*., 2014 char. 127; modified from Pol, 1999a: char. 140): Nasal-premaxilla suture: laterally concave (0), or straight (1).

**Character 118** (Pol *et. al*., 2014 char. 128; modified from Pol, 1999a: char. 141): Nasal lateral edges along the suture with the maxilla: nearly parallel (0), oblique to each other converging anteriorly (1), or oblique to each other diverging anteriorly (2).

**Character 119** (Pol *et. al*., 2014 char. 129; Pol, 1999a: char. 143): Palatine anteromedial margin: exceeding the anterior margin of the palatal fenestrae extending anteriorly between the maxillae (0), or not exceeding the anterior margin of palatal fenestrae (1).

**Character 120** (Pol *et. al*., 2014 char. 130; Pol, 1999a: char. 144): Dorsoventral height of jugal antorbital region respect to infraorbital region: equal or lower (0), or antorbital region more expanded than infraorbital region of jugal (1).

**Character 121** (Pol *et. al*., 2014 char. 131; Pol, 1999a: char. 145): Maxilla-lacrimal contact: partially included in antorbital fossa (0), or completely included (1).

**Character 122** (Pol *et. al*., 2014 char. 132; Pol, 1999a: char. 146): Lateral eustachian tube openings: located posteriorly to the medial opening (0), or aligned anteroposteriorly and dorsoventrally (1).

**Character 123** (Pol *et. al*., 2014 char. 133; Pol, 1999a: char. 147): Anterior process of ectopterygoid: developed (0), or reduced-absent (1).

**Character 124** (Pol *et. al*., 2014 char. 134; Pol, 1999a: char. 148): Posterior process of ectopterygoid: developed (0), or reduced-absent (1).

**Character 125** (Pol *et. al*., 2014 char. 135; Pol, 1999a: char. 149 and Ortega et al., 2000: char. 13): Small neurovascular foramen located in the premaxillo-maxillary suture on the lateral surface of the rostrum (not for large mandibular teeth): absent (0), or present (1).

**Character 126** (Pol *et. al*., 2014 char. 136; Modified from Pol, 1999a: char. 150): Jugal suture with quadratojugal directed: obliquely posteroventrally (0), or vertically as a blunt suture (1).

**Character 127** (Pol *et. al*., 2014 char. 137; modified from Pol, 1999a: char. 151): Orientation of distal carina on upper posterior teeth and mesial carina on lower posterior teeth: oriented parallel to the longitudinal axis of skull (0), or obliquely oriented, at an angle of approximately 45 degrees with the longitudinal axis of the skull (1).

**Character 128** (Pol *et. al*., 2014 char. 138; Pol, 1999a: char. 152): Large and aligned neurovascular foramina on lateral maxillary surface: absent (0), or present (1).

**Character 129** (Pol *et. al*., 2014 char. 139; modified from Pol, 1999a: char. 153): External surface of maxilla: with a single plane facing laterally (0), or with ventral region facing laterally and dorsal region facing dorsolaterally (1).

**Character 130** (Pol *et. al*., 2014 char. 140; modified from Pol, 1999a: char. 154 and Ortega et al., 2000: char. 104): + Mid to posterior elements of the toothrows: crowns not compressed laterally, subcircular in cross section (0), or crowns slightly compressed laterally (1), or roots and crowns highly compressed laterally (2).

**Character 131** (Pol *et. al*., 2014 char. 141; Pol, 1999a: char. 155): Posteroventral corner of quadratojugal: reaching the quadrate condyles (0), or not reaching the quadrate condyles (1).

**Character 132** (Pol *et. al*., 2014 char. 142; modified from Pol, 1999a: char. 156): + Base of postorbital process of jugal: directed posterodorsally (0), or dorsally (1), or anterodorsally (2).

**Character 133** (Pol *et. al*., 2014 char. 143; Pol, 1999a: char. 157): + Postorbital process of jugal: anteriorly placed (0), in the middle (1), or posteriorly positioned (2).

**Character 134** (Pol *et. al*., 2014 char. 144; Pol, 1999a: char. 158 and Ortega et al., 2000: char. 36): Postorbital-ectopterygoid contact: present (0), or absent (1).

**Character 135** (Pol *et. al*., 2014 char. 145; Pol, 1999a: char. 161): Quadratojugal: not ornamented (0), or ornamented in the base (1).

**Character 136** (Pol *et. al*., 2014 char. 146; Pol, 1999a: char. 162): Prefrontal-maxillary contact in the inner anteromedial region of orbit: absent (0), or present (1).

**Character 137** (Pol *et. al*., 2014 char. 147; Pol, 1999a: char. 163): Basisphenoid: without lateral exposure (0), or with lateral exposure on the braincase (1).

**Character 138** (Pol *et. al*., 2014 char. 148; modified from Pol, 1999a: char. 165): Quadrate process of pterygoids: well developed (0), or extremely short and poorly developed, failing to extend along the lateral margin of the basisphenoid and ending far away from the level of the lateral eustachian openings (1).

**Character 139** (Pol *et. al*., 2014 char. 149; modified from Pol, 1999a: char. 166 and Ortega et al., 2000: char. 44): + Quadrate major axis directed: posteroventrally (0), ventrally (1), or anteroventrally (2).

**Character 140** (Pol *et. al*., 2014 char. 150; Pol, 1999a: char. 167): Quadrate distal end: with only one plane facing posteriorly (0), or with two distinct faces in posterior view divided by a ridge, a posterior one and a medial one bearing the foramen aerum (1).

**Character 141** (Pol *et. al*., 2014 char. 151; Pol, 1999a: char. 168): Anteroposterior development of neural spine in axis: well developed covering all the neural arch length (0), or poorly developed, located over the posterior half of the neural arch (1).

**Character 142** (Pol *et. al*., 2014 char. 152; Pol, 1999a: char. 169): Prezygapophyses of axis: not exceeding anterior edge of neural arch (0), or exceeding the anterior margin of neural arch (1).

**Character 143** (Pol *et. al*., 2014 char. 153; Pol, 1999a: char. 170): Postzygapophyses of axis: well developed, curved laterally (0), or poorly developed (1).

**Character 144** (Pol *et. al*., 2014 char. 154; modified from Pol, 1999b: char. 212): Shape of dentary symphysis in ventral view: tapering anteriorly forming an angle (0), U-shaped, smoothly curving anteriorly (1), or lateral edges longitudinally oriented, convex anterolateral corner, and extensive transversely oriented anterior edge (2).

**Character 145** (Pol *et. al*., 2014 char. 155; Pol, 1999b: char. 213): Unsculpted region in the dentary below the tooth row: absent (0), or present (1).

**Character 146** (Pol *et. al*., 2014 char. 156; Buckley and Brochu, 1999: char. 102): Surangular forms only the lateral wall of glenoid fossa and quadratojugal lacks an articular condyle (0) or surangular forms approximately one-third of the glenoid fossa and quadratojugal bears an articular condyle (1).

**Character 147** (Pol *et. al*., 2014 char. 157; modified from Buckley and Brochu, 1999: char. 102): Anterior margin of femur at the area of insertion of *M. puboischiofemoralis internus* 1 (PIFI1) and *M.* *caudofemoralis longus* (CFL): anterior margin of femur linear (0), or bearing a distinct flange (that projects anteriorly the insertion areas for these muscles) and a marked concavity above this region (1).

**Character 148** (Pol *et. al*., 2014 char. 158; modified from Buckley and Brochu, 1999: char. 105): Dentary smooth lateral to seventh alveolus (0), or with lateral concavity for the reception of the enlarged maxillary tooth (1).

**Character 149** (Pol *et. al*., 2014 char. 159; modified from Ortega *et al.*, 1995: char. 1 and Buckley and Brochu, 1999: char. 107): Dorsal edge of dentary slightly concave or straight and subparallel to the longitudinal axis of skull (0), straight with an abrupt dorsal expansion, being straight posteriorly (1), with a single dorsal expansion and concave posterior to this (2), or sinusoidal, with two concave waves (3).

**Character 150** (Pol *et. al*., 2014 char. 160; modified from Ortega *et al*., 1995: char. 2 and Buckley and Brochu, 1999: char. 108): Dentary compression and lateroventral surface anterior to mandibular fenestra: compressed and vertical (0), or not compressed and convex (1).

**Character 151** (Pol *et. al*., 2014 char. 161; modified from Ortega *et al*., 1995: char. 7 and Buckley and Brochu, 1999: char. 110): Splenial: thin posterior to symphysis (0), or splenial robust dorsally posterior to symphysis, being much broader than the lateral alveolar margin of the dentary at the same region (1).

**Character 152** (Pol *et. al*., 2014 char. 162; Ortega *et al*., 1996: char. 13 and Buckley et al., 2000: char. 117): Cheek teeth: not constricted at base of crown (0), or constricted (1).

**Character 153** (Pol *et. al*., 2014 char. 163; Ortega *et al*., 2000: char. 10): Ventral edge of premaxilla located: at the same height that ventral edge of maxilla (0), or located deeper, with the dorsal contour of anterior part of dentary strongly concave (1).

**Character 154** (Pol *et. al*., 2014 char. 164; modified from Ortega *et al*., 2000: char. 19): Maxillary dental implantation: teeth in isolated alveoli (0), or located on a dental groove (1).

**Character 155** (Pol *et. al*., 2014 char. 165; Ortega *et al*., 2000: char. 24): Caudal tip of nasals: converge at sagittal plane forming a transversely straight or a shallow posteriorly concave arch along their posterior margins (0), or caudally separated by an anterior acute sagittal projection of frontals (1).

**Character 156** (Pol *et. al*., 2014 char. 166; Ortega *et al*., 2000: char. 33): Relative length between squamosal and postorbital: squamosal is longer (0), or postorbital is longer (1).

**Character 157** (Pol *et. al*., 2014 char. 167; modified from Ortega *et al*., 2000: character 34): + Jugal portion of postorbital bar: flushes with lateral surface of jugal (0), anteriorly continuous but posteriorly inset (1), or medially displaced and a ridge separates postorbital bar from lateral surface of jugal (2).

**Character 158** (Pol *et. al*., 2014 char. 168; modified from Ortega et al., 2000: char. 42): Outer surface of squamosal along the site of attachment of ear valve groove: laterodorsally oriented and extensive (0), or reduced and vertically oriented (1).

**Character 159** (Pol *et. al*., 2014 char. 169; Ortega *et al*., 2000: char. 47): Quadratojugal spine at caudal margin of infratemporal fenestra: absent (0), or present (1).

**Character 160** (Pol *et. al*., 2014 char. 170; modified from Ortega *et al*., 2000: char. 53): Quadrate condyles with poorly developed intercondylar groove (0), or medial condyle expands ventrally, being separated from the lateral condyle by a deep intercondylar groove (1).

***Character 161** (**mod**. Pol *et. al*., 2014 char. 171; Ortega et al., 2000: char. 62): Exposure of supraoccipital in skull roof: absent (0), present as posterior lamina, without penetrates dorsal skull roof (1); or present and penetrating through skull roof (2);

**Character 162** (Pol *et. al*., 2014 char. 173; Ortega *et al*., 2000: char. 75): Anterior opening of temporo-orbital in dorsal view exposed (0), or hidden in dorsal view and overlapped by squamosal rim of supratemporal fossa (1).

**Character 163** (Pol *et. al*., 2014 char. 174; Pol *et. al*., 2014 char. 174; modified from Ortega *et al*., 2000: char. 90): Foramen intermandibularis oralis: small or absent (0), or big and slot like, with their anteroposterior length being approximately or more than 50% of the depth of the splenial (1).

**Character 164** (Pol *et. al*., 2014 char. 175; modified from Ortega *et al*. 2000: char 98): Coronoid size: short and located below the dorsal edge of the mandibular ramus (0), or anteriorly extended with posterior region elevated at the dorsal margin of the mandibular ramus (1).

**Character 165** (Pol *et. al*., 2014 char. 176; Ortega *et al*., 2000: char. 101): Width of root of teeth respect to crown: much narrower (0), or subequal or wider (1).

**Character 166** (Pol *et. al*., 2014 char. 177; Ortega *et al*., 2000: char. 109): Gap in cervico-thoracic dorsal armor: absent (0) or present (1).

**Character 167** (Pol *et. al*., 2014 char. 178; Ortega *et al*., 2000: char. 130): Lateral contour of snout in dorsal view: straight (0) or sinusoidal (1).

**Character 168** (Pol *et. al*., 2014 char. 179; modified from Ortega *et al*., 2000: char. 138): Pterygoid flanges: laminar and with anteroposteriorly broad lateral end (0) or lateromedially elongated with anteroposteriorly short lateral end (1), or lateromedially short and with narrow lateral end (2).

**Character 169** (Pol *et. al*., 2014 char. 180; modified from Ortega *et al*., 2000: char. 146): Ectopterygoid medial process: single, projected posteriorly on the ventral or lateral surface of the pterygoid flanges (0) or forked, with an accessory anteromedial branch reaching the palatine and forming part of the lateral margin of the choanal opening (1).

**Character 170** (Pol *et. al*., 2014 char. 181; modified from Ortega *et al*., 2000: char. 157): Skull roof: rectangular shaped in dorsal view (0), or trapezoidal shape (1).

**Character 171** (Pol *et. al*., 2014 char. 182; Ortega *et al*., 2000: char. 30): + Prefrontal pillars when integrated in palate: pillars transversely expanded (0), transversely expanded in their dorsal part and columnar (or slightly anteroposteriorly elongated) in the ventral end (1), or longitudinally expanded in their dorsal part and columnar ventrally (2).

**Character 172** (Pol *et. al*., 2014 char. 183; Ortega *et al*., 2000: char. 21): Ventral edge of maxilla in lateral view: straight or convex (0), or sinusoidal (1).

**Character 173** (Pol *et. al*., 2014 char. 184; modified from Ortega *et al*., 2000: char. 156): Position of first enlarged maxillary teeth: second or third alveoli (0), or fourth or fifth (1).

**Character 174** (Pol *et. al*., 2014 char. 185; Pol and Apesteguía, 2005: char. 180): Splenial-dentary suture at symphysis on ventral surface: v-shaped (0), or transversal (1).

**Character 175** (Pol *et. al*., 2014 char. 186; Pol and Apesteguía, 2005: char. 181): Posterior peg at the posterior edge of the mandibular symphysis: absent (0), or present (1).

**Character 176** (Pol *et. al*., 2014 char. 187; Pol and Apesteguía, 2005: char. 182): Posterior ridge on glenoid fossa of articular: present (0), or absent (1).

**Character 177** (Pol *et. al*., 2014 char. 188; modified from Gomani, 1997: char. 46 and Buckley et al., 2000: char. 113): Cusps of posterior teeth: unique apical cusp (0), at least three cusps, a major central cusp with smaller cusps arranged along the mesial and distal margins of the crown (1).

**Character 178** (Pol *et. al*., 2014 char. 189; Pol and Apesteguía, 2005: char. 184): Dorsal surface of mandibular symphysis: flat or slightly concave (0), or strongly concave and narrow, trough shaped (1).

**Character 179** (Pol *et. al*., 2014 char. 190; Pol and Apesteguía, 2005: char. 185): Medial surface of splenials posterior to symphysis: flat or slightly convex (0), or markedly concave (1).

**Character 180** (Pol *et. al*., 2014 char. 191; modified from Pol and Apesteguía, 2005: char. 186): Choanal septum shape: narrow vertical bony sheet (0), or T-shaped bar expanded ventrally (1).

**Character 181** (Pol *et. al*., 2014 char. 192; Pol and Norell, 2004a: char. 164): Cross section of distal end of quadrate: mediolaterally wide and anteroposteriorly thin, being approximately three times as wide as long (0), or subquadrangular or up to twice as broad as anteroposteriorly long (1).

**Character 182** (Pol *et. al*., 2014 char. 193; modified from Pol and Apesteguía, 2005: char. 188): + Lateral surface of dentaries below alveolar margin, at mid to posterior region of tooth row: vertically oriented, continuous with rest of lateral surface of the dentaries (0), or flat surface facing laterally or laterodorsally but divided by a ridge from rest of the lateral surface of the dentaries (1), or posterior region of alveolar facing dorsally, forming a broad alveolar shelf that is strongly inset medially from the lateral surface of the dentaries (2).

**Character 183** (Pol *et. al*., 2014 char. 194; Pol and Norell, 2004a: char. 165): Palatine-pterygoid contact on anterior region of palate: palatines overlie pterygoids (0), or palatines firmly sutured to pterygoids (1).

**Character 184** (Pol *et. al*., 2014 char. 195; Pol *et al*., 2004: char. 164): Ectopterygoid main axis oriented: laterally or slightly anterolaterally (0), or anteriorly, subparallel to the skull longitudinal axis (1).

**Character 185** (Pol *et. al*., 2014 char. 197; modified from Wu *et al*., 1997: char. 105): + Development of distal quadrate body ventral to otoccipital-quadrate contact: distinct (0), incipiently distinct (1), or indistinct (2).

**Character 186** (Pol *et. al*., 2014 char. 198; modified from Wu *et al*., 1997: char. 106): Posterior margin of pterygoid flanges: thin and laminar (0), or dorsoventrally thick, with pneumatic spaces (1).

**Character 187** (Pol *et. al*., 2014 char. 199; Wu *et al*., 1997: char. 108): Postorbital participation in infratemporal fenestra: almost or entirely excluded (0), or bordering infratemporal fenestra (1).

**Character 188** (Pol *et. al*., 2014 char. 200; Wu *et al*., 1997: char. 109): Palatines: form margin of suborbital fenestra (0), or excluded from margin of suborbital fenestra (1).

**Character 189** (Pol *et. al*., 2014 char. 201; Wu *et al*., 1997: char. 110): Angular posterior to mandibular fenestra: widely exposed on lateral surface of mandible (0), or shifted to the ventral surface of mandible (1).

**Character 190** (Pol *et. al*., 2014 char. 203; modified from Wu *et al*., 1997: char. 119): Quadrate process of pterygoid in ventral view: narrow (0), or broad (1).

**Character 191** (Pol *et. al*., 2014 char. 204; Wu *et al*., 1997: char. 121): Pterygoids: not in contact anterior to basisphenoid on palate (0), or pterygoids in contact (1).

**Character 192** (Pol *et. al*., 2014 char. 205; modified from Wu *et al*., 1997: char. 122): Olecranon: well developed (0), or reduced or absent (1).

**Character 193** (Pol *et. al*., 2014 char. 206; Wu *et al*., 1997: char. 123): Cranial table width respect to ventral portion of skull: as wide as ventral portion (0), or narrower than ventral portion of skull (1).

**Character 194** (Pol *et. al*., 2014 char. 207; Wu *et al*., 1997: char. 127): Depression on posterolateral surface of maxilla: absent (0), or present (1).

**Character 195** (Pol *et. al*., 2014 char. 208; modified from Wu *et al*., 1997: char. 128): Anterior palatal fenestra: absent (0), or present (1).

**Character 196** (Pol *et. al*., 2014 char. 209; Pol and Norell, 2004a: char. 179): Paired ridges located medially on ventral surface of basisphenoid: absent (0), or present (1).

**Character 197** (Pol *et. al*., 2014 char. 210; Pol *et al*., 2004a: char. 179): Ventral margin of infratemporal bar of jugal: straight (0), or dorsally arched (1).

**Character 198** (Pol *et. al*., 2014 char. 211; Pol and Norell, 2004a: char. 180): Posterolateral end of quadratojugal: acute or rounded, tightly overlapping the quadrate (0), or with sinusoidal ventral edge and wide and rounded posterior edge slightly overhanging the lateral surface of the quadrate (1).

**Character 199** (Pol *et. al*., 2014 char. 212; Pol and Norell, 2004a : char. 181): Orientation of quadrate body distal to otoccipital-quadrate contact in posterior view: ventrally (0), or ventrolaterally (1).

**Character 200** (Pol *et. al*., 2014 char. 213; Gasparini *et al*., 1993: char. 3): Wedge-like process of the maxilla in lateral surface of premaxilla-maxilla suture: absent (0), or present (1).

**Character 201** (Pol *et. al*., 2014 char. 214; Pol and Norell, 2004b: char. 181): Palpebrals: separated from the lateral edge of the frontals (0), or extensively sutured to each other and to the lateral margin of the frontals (1).

**Character 202** (Pol *et. al*., 2014 char. 215; Pol and Norell, 2004b: char. 182): External surface of ascending process of jugal: exposed laterally (0), or exposed posterolaterally (1).

**Character 203** (Pol *et. al*., 2014: char. 216; Pol and Norell, 2004b: char. 183): Longitudinal ridge on lateral surface of jugal below infratemporal fenestra: absent (0), or present (1).

**Character** **204** (Pol *et. al*., 2014: char. 217; Pol and Norell, 2004b: char. 184): Oblique ridges on the dorsal surface of posterolateral region of squamosal: without ridges (0), or with three curved ridges oriented longitudinally (1).

**Character 205** (Pol *et. al*., 2014: char. 218; Pol and Norell, 2004b: char. 185): Ridge along dorsal section of quadrate-quadratojugal contact: absent (0), or present (1).

**Character 206** (Pol *et. al*., 2014: char. 219; modified from Pol and Norell, 2004b: char. 186): Sharp ridge on the surface of the angular: absent (0), or present on the ventral-most margin (1), or present along the lateral surface (2).

**Character 207** (Pol *et. al*., 2014: char. 220; Pol and Norell, 2004b: char. 187): Longitudinal ridge along the dorsolateral surface of surangular: absent (0), or present (1).

**Character 208** (Pol *et. al*., 2014: char. 221; Pol and Norell, 2004b: char. 188): Dorsal surface of osteoderms ornamented with anterolaterally and anteromedially directed ridges (fleur de lys pattern of Osmólska *et al*., 1997): absent (0), or present (1).

**Character 209** (Pol *et. al*., 2014: char. 222; Pol and Norell, 2004b: char. 189): Cervical region surrounded by lateral and ventral osteoderms sutured to the dorsal elements: absent (0), or present (1).

**Character 210** (Pol *et. al*., 2014: char. 223; Pol and Norell, 2004b: char. 190): Appendicular osteoderms: absent (0), or present (1).

**Character 211** (Pol *et. al*., 2014: char. 224; Ortega *et al*., 2000: character 72): Supratemporal fenestra: present (0), or absent (1).

**Character 212** (Pol *et. al*., 2014: char. 225; modified from Pol and Apesteguía, 2005: char. 220): Flat ventral surface of internal nares septum: parallel sided (0), or tapering anteriorly (1), or tapering posteriorly (2).

**Character 213** (Pol *et. al*., 2014: char. 226; Pol and Apesteguía, 2005: char. 221): + Perinarial fossa: restricted extension (0), extensive, with a distinctly concave surface facing anteriorly (1), or large concave surface facing anteriorly, projecting anteroventrally from the external nares opening toward the alveolar margin (2).

**Character 214** (Pol *et. al*., 2014: char. 227; Sereno *et al*., 2001: char. 67): Premaxillary palate circular paramedian depressions: absent (0), or present located anteriorly on the premaxilla (1).

**Character 215** (Pol *et. al*., 2014: char. 228; modified from Pol and Apesteguía, 2005: char. 223): + Posterolateral region of nasals: flat surface facing dorsally and well separated from the anterodorsal corner of the orbit (0), or expanded posterolaterally reaching the anterior tip of the palpebral facet but limited to the dorsal surface of the skull (1), or well developed posterolateral process that deflects ventrally, forming part of the lateral surface of the snout (2).

**Character 216** (Pol *et. al*., 2014: char. 229; Zaher *et al*., 2006: char. 193): Ventral half of the lacrimal: extending ventroposteriorly widely contacting the jugal (0), or tapering ventroposteriorly, does not contact or contacts the jugal only slightly (1).

**Character 217** (Pol *et. al*., 2014: char. 230; Zaher *et al*., 2006: char. 194): Large foramen on the lateral surface of jugal, near its anterior margin: absent (0), or present (1).

**Character 218** (Pol *et. al*., 2014: char. 231; modified from Zaher *et al*., 2006: char. 195): Procumbent premaxillary alveoli absent (0) or present (1).

**Character 219** (Pol *et. al*., 2014: char. 232; modified from Martinelli, 2003: char. 36, Zaher *et al*., 2006: char. 196, and Turner, 2004: char. 119): Posterolateral end of palatines, completely sutured to the pterygoids (0) or project posterolaterally as rodlike palatine bars (1).

**Character 220** (Pol *et. al*., 2014: char. 233; modified from Zaher *et al*., 2006: char. 197): Participation of ectopterygoid in the lateral margin of the choanal opening: absent or reduced, less than one third of this margin (0), or extensive forming half or more of this margin (1).

**Character 221** (Pol *et. al*., 2014: char. 234; Pol and Norell, 2004a: char. 183): Choanal opening: opened posteriorly and continuous with pterygoid surface (0), or closed posteriorly by an elevated wall formed by the pterygoids (1).

**Character 222** (Pol *et. al*., 2014: char. 235; modified from Zaher *et al*., 2006: char. 198): Ectopterygoid width at its contact with the ventral surface of pterygoid flanges: lateromedially thin process (0), or lateromedially expanded with respect to the shaft of the ectopterygoid, covering approximately the lateral half of the ventral surface of the pterygoid flanges (1).

**Character 223** (Pol *et. al*., 2014: char. 236; Pol and Gasparini, 2009: char. 236): Evaginated maxillary alveolar edges: absent (0), or present as a continuous sheet (1), or present as discrete evaginations at each alveoli (2).

**Character 224** (Pol *et. al*., 2014: char. 237;Pol and Gasparini, 2009: char. 237): Foramen in perinarial depression of premaxilla: absent (0), or present (1).

**Character 225** (Pol *et. al*., 2014: char. 238; Sereno *et al*., 2001: char. 27): Frontal anterior ramus with respect to tip of prefrontal: ending posteriorly (0), or ending anteriorly (1).

**Character 226** (Pol *et. al*., 2014: char. 239; modified from Sereno et al., 2001: char. 68): Premaxillary anterior alveolar margin orientation: vertical (0), or inturned (1).

**Character 227** (Pol *et. al*., 2014: char. 240; Sereno *et al*., 2001: char. 69): Premaxillary tooth row orientation: arched posteriorly from midline (0), or angled posterolaterally, at 120 degree angle (1).

**Character 228** (Pol *et. al*., 2014: char. 241; Sereno *et al*., 2001: char. 70): Last premaxillary tooth position relative to tooth row: anterior (0), or anterolateral (1).

**Character 229**: (Pol *et. al*., 2014: char. 242;) Sutural contact between premaxilla and maxilla on dorsal surface of rostrum posterior to external nares: Premaxillae posterior tip V-shaped, wedging between maxillae (0), or posterior end of premaxillae W-shaped with the anterior tip of maxillae wedging between premaxillae (1).

**Character 230** (Pol *et. al*., 2014: char. 243; modified from Brochu, 1999: char. 108 and from Pol and Gasparini, 2009: char. 243): Maxilla-palatine suture: palatine anteriorly rounded (0), or palatine anteriorly pointed (1), or palatine anterior end slightly invaginated (2), or palatine anterior end divided by a narrow and pointed process of the palatal branches of maxilla (3).

**Character 231** (Pol *et. al*., 2014: char. 244; Pol and Gasparini, 2009: char. 244): Lateral surface of postorbital bar: formed by postorbital and jugal (0), or only by postorbital (1).

**Character 232** (Pol *et. al*., 2014: char. 245; Pol and Gasparini, 2009: char. 245): Enlarged foramen at anterior end of surangular groove: absent (0), or present (1).

**Character 233** (Pol *et. al*., 2014: char. 246; Pol and Gasparini, 2009: char. 246): Shape of antorbital fossa: subcircular or subtriangular (0), or elongated, low, and oriented obliquely (1).

**Character 234** (Pol *et. al*., 2014: char. 247; Pol and Gasparini, 2009: char. 247): Prefrontal lateral development: reduced (0), or enlarged, extending laterally over the orbit (1).

**Character 235** (Pol *et. al*., 2014: char. 248; Pol and Gasparini, 2009: char. 248): Foramen for the internal carotid artery: reduced, similar in size to the openings for cranial nerves IX-XI (0), or extremely enlarged (1).

**Character 236** (Pol *et. al*., 2014: char. 249; Pol and Gasparini, 2009: char. 249): Squamosal posterolateral region, lateral to paroccipital process: narrow (0), or bearing a subcircular flat surface (1).

**Character 237** (Pol *et. al*., 2014: char. 250; Pol and Gasparini, 2009: char. 250): Posteromedial branch of squamosal oriented: transversely (0), or posterolaterally (1).

**Character 238** (Pol *et. al*., 2014: char. 251; Pol and Gasparini, 2009: char. 251): Dorsal margin of squamosal occipital flange: straight (0), or dorsally concave (1).

**Character 239** (Pol *et. al*., 2014: char. 252; Pol and Gasparini, 2009: char. 252): Sculpture in external surface of rostrum: absent (0), or present (1).

**Character 240** (Pol *et. al*., 2014: char. 253; Pol and Gasparini, 2009: char. 253): Longitudinal depressions on palatal surface of maxillae: absent (0), or present (1).

**Character 241** (Pol *et. al*., 2014: char. 254; Pol and Gasparini, 2009: char. 254): Angle between medial and anterior margins of supratemporal fossa: approximately 90 degrees (0), or approximately 45 degrees (1).

**Character 242** (Pol *et. al*., 2014: char. 255; Pol and Gasparini, 2009: char. 255): Transverse process of sacral vertebrae directed: laterally (0), or markedly deflected ventrally (1).

**Character 243** (Pol *et. al*., 2014: char. 256; Pol and Gasparini, 2009: char. 256): Prefrontal and lacrimal around orbits: forming flat rims (0), or evaginated, forming elevated rims (1).

**Character 244** (Pol *et. al*., 2014: char. 257; Pol and Gasparini, 2009: char. 257): Nasal bones: paired (0), or partially or completely fused (1).

**Character 245** (Pol *et. al*., 2014: char. 258; Brochu, 1997: char. 3): Posterior half of axis neural spine wide (0) or narrow (1).

**Character 246** (Pol *et. al*., 2014: char. 259; Brochu, 1997: char. 19): Axial hypapophysis without (0) or with deep fork (1).

**Character 247** (Pol *et. al*., 2014: char. 260; Brochu, 1997: char. 27): Olecranon process of ulna narrow and subangular (0) or wide and rounded (1).

**Character 248** (Pol *et. al*., 2014: char. 261; Brochu, 1997: char. 29): M. teres major and M. dorsalis scapulae insert separately on humerus; scars can be distinguished dorsal to deltopectoral crest (0) or insert with common tendon; single insertion scar (1).

**Character 249** (Pol *et. al*., 2014: char. 262; modified from Brochu, 1997: char. 53): Anterior dentary alveoli project anterodorsally or weakly procumbent (0) or strongly procumbent (1).

***Character 250** (**mod**. Pol *et. al*., 2014: char. 264; Brochu, 1997: char. 91): Ectopterygoid abuts maxillary toothrrow (0), ectopterygoid near maxillary toothrow about an alveolus lenght; or maxilla broadly separates ectopterygoid from maxillary toothrow (1) ectopterygoid broadly separated from upper tooth row by maxillary posterior shelf (2).

**Character 251** (Pol *et. al*., 2014: char. 266; modified from Brochu, 1997: char. 103): Lateral margins of frontal: flush with skull surface (0), or elevated, forming ridged orbital margins (1).

**Character 252** (Pol *et. al*., 2014: char. 267; Brochu, 1997: char. 130): Capitate process of laterosphenoid oriented laterally (0) or anteroposteriorly (1) toward midline.

**Character 253** (Pol *et. al*., 2014: char. 268; modified from Brochu, 1997: char. 141): Paroccipital process development lateral to cranioquadrate opening: short (0) or long (1).

**Character 254** (Pol *et. al*., 2014: char. 269; modified from Norell, 1988: char. 32 by Brochu, 1997: char. 149): Ectopterygoid extends (0) or does not extend (1) to posterior tip of lateral pterygoid flange at maturity.

**Character 255** (Pol *et. al*., 2014: char. 270; Brochu, 1997: char. 153): Incisive foramen completely situated far from premaxillary toothrow, at the level of the second or third alveolus (0) or abuts premaxillary toothrow (1).

**Character 256** (Pol *et. al*., 2014: char. 271; modified from Turner, 2004: character 126): Ventral surface of choanal septum smooth to slightly depressed (0) or marked by an acute groove (1).

**Character 257** (Pol *et. al*., 2014: char. 272; modified from Turner, 2006: char. 128): Proximal-most portion of fibular head straight sided to weakly developed posteriorly (0) or very sharply projecting posteriorly, forming distinct extension (1).

**Character 258** (Pol *et. al*., 2014: char. 273; Turner, 2006: char. 129): Posterior process of cervical rib shaft lacks (0) or possesses (1) a posterodorsally projecting spine at the junction with the tubercular process.

**Character 259** (Pol *et. al*., 2014: char. 275; Pol *et al*., 2009: char. 275): Jugal below the anteroventral corner of the orbit: lacks (0) or possesses an emarginated orbital margin and an associated depression located on the dorsal region of the jugal (1).

**Character 260** (Pol *et. al*., 2014: char. 278; Pol *et al*., 2009: char. 278): Anterior half of palatines between suborbital fenestrae: lateral margins are parallel to subparallel (0) or flared anteriorly (1).

**Character 261** (Pol *et. al*., 2014: char. 279; modified from Pol *et al*., 2009: char. 279 and Montefeltro *et al*., 2011: char. 41): + Posterior half of palatines between suborbital fenestrae: lateral margins are parallel to subparallel (0) or slightly constricted and flared posteriorly (1), or markedly constricted lateromedially at its posterior portion and flaring posteriorly (2).

**Character 262** (Pol *et. al*., 2014: char. 280; Pol *et al*., 2009: char. 280): Posteroventral margin of the angular straight or gently arched dorsally (0) or strongly arched dorsally (1).

**Character 263** (Pol *et. al*., 2014: char. 282; Pol *et al*., 2009: char. 282):Fibular shaft distal to iliofibularis trochanter straight (0) or bowed posteriorly (1).

**Character 264** (Pol *et. al*., 2014: char. 283; Larsson and Sues, 2007: char. 55): Premaxillary teeth 1 and 2, position: separated like adjacent teeth (0), or nearly confluent (1).

**Character 265** (Pol *et. al*., 2014: char. 284; Larsson and Sues, 2007: char. 60): Large nutrient foramen on palatal surface of premaxilla-maxilla contact: small or absent (0), or present (1).

**Character 266** (Pol *et. al*., 2014: char. 285; Larsson and Sues, 2007: char. 62): Incisive foramen size: present and large (length equal or more than half the greatest width of premaxillae) (0), or present or small (1), or absent (2).

**Character 267** (Pol *et. al*., 2014: char. 286; Larsson and Sues, 2007: char. 66): Premaxilla-maxilla lateral fossa excavating alveolous of last premaxillary tooth: no (0), or yes (1).

**Character 268** (Pol *et. al*., 2014: char. 287; Pol and Powell, 2011: char. 287): Shape of antorbital fenestra: rounded or dorsoventrally high (0), or low and elongated, slit-like (1).

**Character 269** (Pol *et. al*., 2014: char. 288; Pol and Powell, 2011: char. 288): Nasal exposure on lateral surface of rostrum: deflecting gradually from the dorsal surface (0), or deflecting abruptly, forming an almost 90 degree angle between the dorsal and lateral surfaces (1).

**Character 270** (Pol *et. al*., 2014: char. 290; Pol and Powell, 2011: char. 290): Dorsal surface of frontal: flat or slightly concave (0), with a broad basin-like depressed area bordered posteriorly by a transversal ridge (1).

**Character 271** (Pol *et. al*., 2014: char. 291; Pol and Powell, 2011: char. 291): Rugose surface on palatal surface of maxilla posterior to last tooth: absent (0), or present (1).

**Character 272** (Pol *et. al*., 2014: char. 292; Pol and Powell, 2011: char. 292): Ectopterygoid-palatine contact posterior to the suborbital fenestra: not contacting (0), or contacting (1).

**Character 273** (Pol *et. al*., 2014: char. 293; modified from Andrade and Bertini 2008a: char. 103 by Pol and Powell, 2011: char. 293): Pterygoid ventral surface at the origin of the pterygoid flanges: flat or slightly concave (0), or bearing a pterygoid parachoanal fossa located laterally or posterolaterally to choanal opening; a distinctly depressed area that perforates the pterygoid flanges in some taxa (1).

**Character 274** (Pol *et. al*., 2014: char. 294; modified from Turner and Buckley, 2008: char. 286): Jugal, anterior and posterior processes: inline dorsoventrally (0) or dorsal margin of anterior and posterior processes at a sharp angle to one another, both processes slope ventrally to form a strongly arched jugal (1).

**Character 275** (Pol *et. al*., 2014: char. 295; Larsson and Sues, 2007: char. 31): Length of anterior process of quadratojugal: either short or absent (0), or from long (less than half length of lower temporal bar) to moderate (one third of lower temporal bar) (1), or long (greater than half of lower temporal bar) (2).

**Character 276** (Pol *et. al*., 2014: char. 296; Pol *et al*., 2012: char. 296). Prezygapophyseal process of anterior cervical vertebrae: anterodorsally projected and straight or slightly recurved (0), or dorsally projected and strongly recurved (1).

**Character 277** (Pol *et. al*., 2014: char. 297; Pol *et al*., 2012: char. 297). Prezygapophyseal process of anterior to mid cervical vertebrae in lateral view: anterior margin straight or evenly convex (0), or anterior margin bearing a distinct bulge at the midpoint of the prezygapophyseal process (1).

**Character 278** (Pol *et. al*., 2014: char. 298; Pol *et al*., 2012: char. 298). Shape of the articular surface of the parapophysis in posterior cervical and anterior dorsals: subcircular or ovoid with the major axis oriented anteroposteriorly (0), or subtriangular or ovoid with major axis oriented dorsoventrally (1).

**Character 279** (Pol *et. al*., 2014: char. 299; Pol *et al*., 2012: char. 299). Dorsal migration of parapophysis on the neural arch on mid dorsals: dorsal vertebrae 4 to 9 showing a gradual dorsal migration of parapophysis, with at least two vertebrae bearing the parapophysis on the neural arch pedicles, well below the diapophysis (0), or abrupt change in position of parapophysis, with dorsal 4 bearing the parapophysis at the neurocentral suture and dorsal 5 with parapophysis leveled with diapophysis forming a transverse process (1).

**Character 280** (Pol *et. al*., 2014: char. 300; Pol *et al*., 2012: char. 300). Medial surface of prezygapophyseal process of anterior to mid cervical vertebrae: with an ovoid or triangular depression close to the neural canal (0), or flat or slightly convex (1).

**Character 281** (Pol *et. al*., 2014: char. 301; Pol *et al*., 2012: char. 301). Spinopostzygapophyseal lamina in dorsal vertebrae: absent (0), or present as a high and sharp lamina (1).

**Character 282** (Pol *et. al*., 2014: char. 302; Pol *et al*., 2012: char. 302). Distinct rounded depression on the dorsal surface of neural arches of the anterior to mid dorsal vertebrae, located between the base of the neural spine and the postzygapophyseal process: absent (0), or present (1).

**Character 283** (Pol *et. al*., 2014: char. 303; Pol *et al*., 2012: char. 303). Relative position of the transverse process andthe postzygapophysis in mid dorsal vertebrae: postzygapophysis located dorsally to the transverse process (0), or postzygapophysis leveled with the transverse process (1).

**Character 284** (Pol *et. al*., 2014: char. 304; Pol *et al*., 2012: char. 304). Dorsolateral end of first sacral rib: located at the level of the neural canal (0), or dorsoventrally expanded, projecting dorsally above the level of the neural canal (1).

**Character 285** (Pol *et. al*., 2014: char. 305; Buckley and Brochu, 1999; char 106).Scapular blade no more than twice the length of the scapulocoracoid articulation (0), or scapular blade very broad and greater than twice the length of the scapulocoracoid articulation (1).

**Character 286** (Pol *et. al*., 2014: char. 306; Pol *et al*., 2012: char. 306). Insertion mark dorsal to the glenoid facet of the scapula for the attachment of the *M. triceps*: present as a well-developed ridge or tubercle (0), or absent (1).

**Character 287** (Pol *et. al*., 2014: char. 307; Pol *et al*., 2012: char. 307). Recess ventral to the glenoid facet of the coracoid: shallow and smoothly concave surface (0), or deep recess strongly concave in lateral view, overhung by a large ventral projection of the glenoid facet (1).

**Character 288** (Pol *et. al*., 2014: char. 308; Pol *et al*., 2012: char. 308). Ventral expansion of the coracoid: larger or equal to the proximal expansion (0), or less expanded than the proximal region (1).

**Character 289** (Pol *et. al*., 2014: char. 309; Pol *et al*., 2012: char. 309). Orientation of the area of instertion of *M. subscapularis* above the internal tuberosity of the humerus: obliquely oriented in anterior view, with the area of insertion facing proximomedially (0), or vertically oriented in anterior view, with the area of insertion facing medially (1).

**Character 290** (Pol *et. al*., 2014: char. 310; Pol *et al*., 2012: char. 310). Anterior projection and profile of deltopectoral crest in humerus: Well-developed crest bearing a pointed tubercle for the insertion of the supracoracoideus complex (*sensu* Meers, 2003) (0), or low and anteriorly convex in lateral view, lacking a well-developed tubercle (1).

**Character 291** (Pol *et. al*., 2014: char. 311; Pol *et al*., 2012: char. 311). Proximal third of the deltopectoral crest: originating at the proximolateral corner of the humerus and running distally along the proximal region of the lateral margin of the humerus (0), or proximal origin medially displaced from the proximolateral corner of the humerus and running distally, leaving an anteriorly facing concave surface between the crest and the lateral margin of the anterior surface of the humerus (which probably corresponds to the insertion area of the *M. coracobrachialis brevis dorsalis*) (1).

**Character 292** (Pol *et. al*., 2014: char. 312; Pol *et al*., 2012: char. 312). Orientation and extension of the distal half of the deltopectoral crest: running along the lateral edge of the humerus or slightly deflected medially reaching, at the most, the lateromedial midpoint of the humeral shaft (0), or strongly deflected medially, surpassing the lateromedial midpoint of the anterior surface of the humeral shaft (1).

**Character 293** (Pol *et. al*., 2014: char. 313; Pol *et al*., 2012: char. 313). Anterior surface of the distal half of the deltopectoral crest: lateromedially narrow, forming a sharp ridge, in some cases with a slightly bulged apex (0), or lateromedially broad forming an expanded anterior surface (1).

**Character 294** (Pol *et. al*., 2014: char. 314; Pol *et al*., 2012: char. 314). Circular depression on the posterior surface of the proximal end of the humerus, related to the insertion of the *M. scapulohumeralis caudalis*: absent (0), or present (1).

**Character 295** (Pol *et. al*., 2014: char. 315; Pol *et al*., 2012: char. 315). Posterior surface of the humerus with a distinct, sharply-delimited, pit at the proximodistal level of the apex of the deltopectoral crest, usually related to the insertion of the M. teres major and *M. latissimus dorsi* (*sensu* Meers, 2003): absent (0), or present (1).

**Character 296** (Pol *et. al*., 2014: char. 288; Pol *et al*., 2012: char. 316). Anteroproximal end of the distal articular surface of the humerus: continuous with the anterior surface of the humeral shaft or incipiently projected anteriorly (0), or separated from the humeral shaft by a distinct step, formed by a concave and proximally facing shelf surface that extends lateromediallyacross the entire width of the distal humerus and is bound by two well developed supracondylar ridges (1).

**Character 297** (Pol *et. al*., 2014: char. 317; Pol *et al*., 2012: char. 317). Lateral and medial surface of distal end of humerus: flat and anteroposteriorly broad, similar in anteroposterior length to the lateromedial width of the distal end of humerus (0), or convex and reduced in comparison with the lateromedial width of the distal humerus (1).

**Character 298** (Pol *et. al*., 2014: char. 318; Pol *et al*., 2012: char. 318). Articular surface for the ulna on the radiale: facing posterolaterally (0), or facing posteriorly, not visible in lateral view (1).

**Character 299** (Pol *et. al*., 2014: char. 319; Pol *et al*., 2012: char. 319). Proximodistal development of articular surface for the ulna on the radiale: short and wide, being up to than 30% of the total length of the radiale (0), or proximodistally elongated, being more than 40% of the total length of the radiale (1).

**Character 300** (Pol *et. al*., 2014: char. 320; Pol *et al*., 2012: char. 320). Distal region of articular surface for the ulnare in the radiale: merging gradually with the posterolateral surface of the ulnar shaft (0), or usually triangular shaped, and separated from the ulnar shaft by a distinct step (1).

**Character 301** (Pol *et. al*., 2014: char. 321; Pol *et al*., 2012: char. 321). Proximal region of articular surface for the ulnare in the radiale: divided from the articular surface for the ulna by a crest, creating a distinct articular surface for the ulnare (0), or continuous with the articular surface for the ulna (1).

**Character 302** (Pol *et. al*., 2014: char. 322; Pol *et al*., 2012: char. 322). Anterior surface of radiale: smoothly convex (0), or bearing a proximodistal crest that extends along the shaft dividing the anterior surface of the radiale (1).

**Character 303** (Pol *et. al*., 2014: char. 323; Pol *et al*., 2012: char. 323). Distolateral expansion of the ulnare: absent, as (or less) expanded as the distomedial corner of the ulnare (0), or distinctly expanded and projecting more distally than the distomedial corner of the ulnare, forming a distinct process (“ulnar anterior projection” *sensu* Nascimento and Zaher, 2010) (1).

**Character 304** (Pol *et. al*., 2014: char. 324; Pol *et al*., 2012: char. 324). Lateromedial width of shaft of metacarpal I: as broad as the shaft of other metacarpals (0), or broader than other metacarpals, being the digit I the most robust element of the metacarpus (1).

**Character 305** (Pol *et. al*., 2014: char. 325; Pol *et al*., 2012: char. 325). Development of the postacetabular process of the ilium: well developed as a distinct process that extends anteroposteriorly at least 60% of the acetabular length (0), or extremely reduced or absent, extending anteroposteriorly not more than 50% of the acetabular length (1).

**Character 306** (Pol *et. al*., 2014: char. 326; Pol *et al*., 2012: char. 326). Posterior end of the postacetabular process: tapering posteriorly and ending in an acute tip (0), or subrectangular shaped with the posterior end vertically oriented, with its dorsoventral height being at least 60% of the height at the origin of the postacetabular process (1).

**Character 307** (Pol *et. al*., 2014: char. 327; Pol *et al*., 2012: char. 327). Orientation of the ventral margin of the postacetabular process: posterodorsally directed (0), or horizontally or slightly posteroventrally deflected (1).

**Character 308** (Pol *et. al*., 2014: char. 328; Pol *et al*., 2012: char. 328). Dorsoventral position of the ventral margin of the postacetabular process (along its posterior third): located at the same height or dorsally than the acetabular roof (0), or located at or ventrally than the dorsoventral midpoint of the acetabular height (1).

**Character 309** (Pol *et. al*., 2014: char. 329; Pol *et al*., 2012: char. 329). Relative position of supraacetabular crest and iliac blade at the anterior region of the acetabulum of the ilium: well separated from each other by a shallow concave surface (0), merged together forming a single rugose surface for the insertion of the *M. iliotibialis* 1 and 2 (*sensu* Romer, 1923) (1).

**Character 310** (Pol *et. al*., 2014: char. 330; Pol *et al*., 2012: char. 330). Anterior peduncle of ilium: shallow concavity separating the anterior and posterior articular surface of the anterior iliac peduncle (0), or deep notch incising two well developed articular surfaces, which project anteroventrally forming an acute angle between them (1).

**Character 311** (Pol *et. al*., 2014: char. 331; Pol *et al*., 2012: char. 331). Development of greater trochanter on proximal femur: prominent, ridge-like lateral border that separates lateral surface of proximal femur from a flat posterior surface of proximal femur reaching down to the level of the fourth trochanter (0), or proximodistally short trochanteric surface lacking a distinct ridge that separates the lateral and posterior surfaces of the proximal femur and ending well above the fourth trochanter (1).

**Character 312** (Pol *et. al*., 2014: char. 332; Pol *et al*., 2012: char. 332). Medial edge of the greater trochanter: low ridge or convex surface (0), or forms a prominent sharp long crest offset from the medial surface of the femur (1).

**Character 313** (Pol *et. al*., 2014: char. 334; Pol *et al*., 2012: char. 334). Lateral supracondylar ridge on anterior surface of distal femur: prominent and broad lateral suprancondylar ridge separating the anterior concave surface of femur from the lateral surface (0), or absence of well developed lateral suprancondylar ridge, anterior surface of femur flat or slightly concave and continuous with the lateral surface of the distal femur (1).

**Character 314** (Pol *et. al*., 2014: char. 335; Pol *et al*., 2012: char. 335). Distal half of tibial shaft in lateral view: straight (0), or posteriorly bowed (1).

**Character 315** (Pol *et. al*., 2014: char. 336; Pol *et al*., 2012: char. 336). Tibial shaft in anterior or posterior view: straight or only slightly bowed (0), or markedly bowed laterally (1).

**Character 316** (Pol *et. al*., 2014: char. 337; Pol *et al*., 2012: char. 337). Distal projection of tibial articular surfaces: medial region of distal articular surface of distal tibia extends further distally than the lateral region, forming a strongly oblique distal margin of the tibia (0), or medial and lateral regions subequally extended, with distal margin subhorizontally oriented (1).

**Character 317** (Pol *et. al*., 2014: char. 338; Pol *et al*., 2012: char. 338). Anterior margin of the tibial facet on the astragalus: forming a well-defined ridge that reaches medially the ball-shaped region for the articulation of metatarsal I-II and closes the proximomedial corner of the anterior hollow of the astragalus (0), or forming a low ridge that is medially separated by a notch from the ball-shaped region for the articulation of the metatarsals I-II, failing to close the proximomedial corner of the anterior hollow (1).

**Character 318** (Pol *et. al*., 2014: char. 339; Pol *et al*., 2012: char. 339). Planar and proximal calcaneal surfaces on the astragalus: connected to each other forming a continuous articular surface that articulates with the calcaneal condyle, the margin of which forms the distolateral ridge-like margin of the anterior hollow of the astragalus (0), or separated from each other forming two distinct articular surfaces for the planar and proximal articular surfaces of the calcaneum (1).

**Character 319** (Pol *et. al*., 2014: char. 340; Pol *et al*., 2012: char. 340). Articular surface for the distal tarsal 3 on astragalus: proximodistally leveled with the distal end of the planar calcaneal facet and distal surface of the ball-like articulation for metatarsals I-II, in anterior view these structures form an elevated ridge that close the distal corner of the anterior hollow of the astagalus (0), or proximally inset creating a clear separation between the planar facet and the distal surface of the ball like articulation for metatarsals I-II, and leaving a distal notch along the margins of the anterior astragalar hollow (1).

**Character 320** (Pol *et. al*., 2014: char. 341; Pol *et al*., 2012: char. 341). Astragalar-tarsal ligament pit on astragalus (*sensu* Sertich and Groenke, 2010) at the distal end of the anterior hollow: not differentiated from the rest of the anterior hollow of the astragalus (0), or distinct depression separated from the anterior hollow by an obliquely oriented ridge running along the proximolateral margin of the astragalar-tarsal ligament pit (1).

**Character 321** (Pol *et. al*., 2014: char. 342; Pol *et al*., 2012: char. 342). Development of proximal astragalar depression, located posteriorly to the tibial facet of the astragalus: shallow concave depression (0), or deep depression with sharply delimited medial and anterior margins, forming a true astragalar fossa (1).

**Character 322** (Pol *et. al*., 2014: char. 343; Pol *et al*., 2012: char. 343): + Shape of the fibular facet on the astragalus: subtrapezoidal with the proximodistal height of anterior margin higher than the posterior margin (0), or subrectangular with subequal anterior and posterior margins (1), or trapezoidal with the proximodistal height of its anterior margin lower that the posterior margin (2).

**Character 323** (Pol *et. al*., 2014: char. 344; Pol *et al*., 2012: char. 344): Ridge along dorsolateral edge of calcaneal tuber and associated fossa medially to the ridge: present (0), or absent (1).

**Character 324** (Pol *et. al*., 2014: char. 345; Pol *et al*., 2012: char. 345): Calcaneal tuber with lateral tubercle and crest extending anteriorly from it: present (0), or absent (1).

**Character 325** (Pol *et. al*., 2014: char. 346; Pol *et al*., 2012: char. 346): Posterolateral region of the facet for distal tarsal 4 in calcaneum: subrectangular with a right-angled posterolateral corner (0), or subtriangular shaped with an oblique posterolateral margin (1).

**Character 326** (Pol *et. al*., 2014: char. 347; Pol *et al*., 2012: char. 347): Calcaneum with posterior astragalar facet: subtriangular with proximal and lateral margins forming a right angle and an oblique medioplantar edge (0), or proximal and plantar edges subparallel to each other connected through a broad and rounded medial margin (1).

**Character 327** (Pol *et. al*., 2014: char. 348; modified from Novas *et al*., 2009: char. 231): Anterior margin of the suborbital fenestra: maxilla precludes the ectopterygoid-palatine contact at the anterior margin of the suborbital fenestra (0), or ectopterygoid prjects anteromedially contacting (or almost reaching) the anterolateral end of the palatine, mostly or completely excluding the maxilla from the anterior margin of the suborbital fenestra (1).

**Character 328** (Pol *et. al*., 2014: char. 349; modified from Novas *et al*., 2009: char. 232): Posterior end of the glenoid facet of articular: located above the surangular-angular suture (0), or ventrally recessed, located at or below the dorsoventral midpoint of the posterior mandibular ramus (i.e., surangular forming a high lateral wall that covers the posterior end of the glenoid facet) (1).

**Character 329** (Pol *et. al*., 2014: char. 350; Novas *et al*., 2009: char. 233): Ventral margin of the lateral edge of squamosal, above otic recess: straight or slightly sinusoidal (0) or bearing a highly convex ventral outgrowth anteriorly to a small but highly concave concavity located at the level of the otic aperture (1).

**Character 330** (Pol *et. al*., 2014: char. 351) Jugal anteroventral process between maxilla and ectopterygoid: absent (0), present, jugal extending anteriorly a short triangular process that wedges between the ecotpterygoid and maxilla on the lateroventral surface of the skull at the level of the orbits (“sickle-like medial process present on the ventral surface of the anterior jugal ramus” sensu Andrade and Bertini, 2008a) (1).

**Character 331** (Pol *et. al*., 2014: char. 352): Posterior maxillary surface at the anteroventral region of the orbit: dorsoventrally thin and horizontal, forming the posterior end of the palatal branch (0), or forming an orbital lamina, a vertical wall that restricts the opening of the nasal cavity into the orbit (1).

**Character 332** (Pol *et. al*., 2014: char. 353): Frontal shape along its suture with the prefrontal: relatively broad and tapering gradually anteriorly (0), or broad tabular-shaped with lateral sutures with prefrontals parallel to each other (1).

**Character 333** (Pol *et. al*., 2014: char. 354;): Temporo-orbital foramen: enclosed between the parietal and squamosal (0), or completely enclosed within squamosal (1).

**Character 334** (Pol *et. al*., 2014: char. 355;): Ornamentation on dorsal surface of the posterolateral process of squamosal: present (0), or absent (1).

**Character 335** (Pol *et. al*., 2014: char. 356; modified from Sereno and Larsson, 2009: char. 69): + Anterior extension of the otic recess: restricted to the squamosal (0), or extends on the posterior region of the lateral surface of the postorbital (1), or extends along the entire length of the postorbital, which has an anterior transverse lamina that separates the otic recess from the orbit (2).

**Character 336** (Pol *et. al*., 2014: char. 357; modified from Andrade and Bertini 2008a: char. 70): + Quadrate contact with basioccipital: absent (0), or located on the ventral surface of the braincase (1), or well developed medial crest of quadrate meets the basioccipital on the occipital surface of the skull, excluding the exoccipital from the ventral margin of the occipital surface (2).

**Character 337** (Pol *et. al*., 2014: char. 358): Supraoccipital lateromedial width: extensive, occupying half of the lateromedial width of the occipital table (0), or narrow, occupying less than one third of the lateromedial width of the occipital table (1).

**Character 338** (Pol *et. al*., 2014: char. 359): Entrance of internal carotid artery into occipital surface of the skull: located close to the ventral end of the exoccipital, ventrally separated from the opening for the cranial nerves IX-XI (0), located dorsally, close to and within the same depression as the foramina for the cranial nerves IX-XI (1).

**Character 339** (Pol *et. al*., 2014: char. 360; Turner and Sertich, 2010: char 297): Sagittal ridge on the ventral half of the posterior surface of the basioccipital: absent or poorly developed (0), or present (1).

**Character 340** (Pol *et. al*., 2014: char. 361): Palatine width at the level of the anterior end of suborbital fenestra: broad, close to half the width of the maxillary palate (0), or narrow, approximately 25% the withd of the maxillary palate (1).

**Character 341** (Pol *et. al*., 2014: char. 362; modified from Montefeltro *et al*. 2011: char. 44): Longitudinal sulcus (and associated foramina) on the ventral surface of palatines between suborbital fenestra:absent (0), or present (1).

**Character 342** (Pol *et. al*., 2014: char. 363): Anterior region of dentary symphysis in ventral view: lacking a distinct anterior process, lateral margin of the dentaries diverge gradually (0), or having a distinct anterior process with parallel lateral margins (1).

**Character 343** (Pol *et. al*., 2014: char. 365): Size of neurovascular foramina on mid to posterior region of alveolar edge of the dentary: small (0), or extremely large, being approximately as anteroposteirorly long as an alveolus (1).

**Character 344** (Pol *et. al*., 2014: char. 366): Sutural contact between dentary and surangular above the external mandibular fenestra: dentary overlaps surangular (0), or surangular overlaps dentary (1), or interdigitated and vertically oriented suture (2).

**Character 345** (Pol *et. al*., 2014: char. 367; modified from Andrade and Bertini, 2008a: char 113; Turner and Buckley, 2008: char 289): Posterodorsal branch of dentaries (above external mandibular fenestra): single branch sutured to the ventral margin of the anterior process of the surangular (0), divided into a ventral and a dorsal process exposed on the lateral surface of the lower jaw, the dorsal process fits into the large notch between the medial and lateral rami of the bifurcated anterior end of the surangular (1).

**Character 346** (Pol *et. al*., 2014: char. 368; modified from Brochu, 1999: character 41) +: Location of the anterior opening for the mandibular nerve (V 3 ): located at or close to the rostral margin of the splenial (0), or enclosed in the splenial and located on the anterior region of splenial (*i.e*., anterior foramen intermandibularis oralis sensu Brochu, 1999) (1), or enclosed in the splenial but located at the anteroposterior midpoint of the splenial (2).

**Character 347** (Pol *et. al*., 2014: char. 369): Foramen intermandibularis caudalis: present and enclosed between the angular and splenial below the mandibular adductor fossa (0), or absent with imperforated splenial-angular suture (1)

**Character 348** (Pol *et. al*., 2014: char. 370): Location of the posterior peg in mandibular symphysis: located on the ventral surface of symphysis (0), or located above the ventral surface, on the posterior surface of the symphysis (1)

**Character 349** (Pol *et. al*., 2014: char. 371): Smooth elongated fossa extending along ventral margin of external mandibular fenestra on the angular: absent, lateral surface of the angular reaching the ventral edge of the fenestra (0), or present, separated from the lateral surface of the angular by a sharp ridge (1).

**Character 350** (Pol *et. al*., 2014: char. 373): Dorsal surfaces of the lateral glenoid facet and the lateral flange of the retroarticular process: glenoid facet separated from the retroarticular surface by a ridge or a step (0), or continuous (1).

**Character 351** (Pol *et. al*., 2014: char. 374): Length of the lateral flange of the retroarticular process relative to the lateromedial width of the glenoid facets of the articular: shorter (0), or approximately the same length or longer (1).

**Character 352** (Pol *et. al*., 2014: char. 375): Rounded bulge at the posterior end of the lateral flange of the retroarticular process: absent (0), or present (1).

**Character 353** (Pol *et. al*., 2014: char. 376): Orientation of the ridge on the dorsal surface of retroarticular process that divides the of the lateral and medial flanges of the retroarticular process: directed posteriorly, parallel to the longitudinal axis of the mandibular ramus (0), or directed posterolaterally, approximately at 45 degrees with the longitudinal axis of the mandibular ramus (1).

**Character 354** (Pol *et. al*., 2014: char. 377): Small bulge located proximally on the medial flange of the retroarticular process, posteriorly to the medial glenoid facet of the articular and associated with the foramen aerum in some taxa: absent (0), or present (1).

**Character 355** (Pol *et. al*., 2014: char. 378): Anteromedial end of medial flange of the retroarticular process: connected to the posteromedial corner of the medial glenoid facet of the articular through a dorsally directed crest (0), or extending anteriorly as a distinct anterior process up to the level of the anteroposterior midpoint of the medial glenoid of the articular (1), or projecting anteroventrally as deep pendant process (2).

**Character 356** (Pol *et. al*., 2014: char. 379): Orientation of medial flange of the retroarticular process: facing dorsally or slightly dorsomedially, having a similar orientation to the lateral flange to the medial flange of the retroarticular process (0), or facing medially, strongly deflected and forming an angle of approximately 90 degrees with the dorsal surface of the lateral flange (1).

**Character 357** (Pol *et. al*., 2014: char. 380): Medial edge of the medial flange of the retroarticular process: straight or slightly convex (0), or strongly convex forming a paddle-shaped medial flange; its margin forms an extensive arch of approximately half circumference when viewed in dorsal view (1).

**Character 358** (Pol *et. al*., 2014: char. 381; modified from Andrade and Bertini 2008a: char. 128 and Turner and Sertich, 2010: char. 296): Transitional tooth located at the contact between the premaxilla and maxilla, both of which contribute to the alveolar walls: absent (0), or present (1).

**Character 359** (Pol *et. al*., 2014: char. 382): Number of strongly procumbent teeth on the anterior region the mandibular symphysis: one tooth on each dentary (0), or two procumbent teeth on each dentary (1).

**Character 360** (Pol *et. al*., 2014: char. 383): Implantation of lower incisiviforms: in separate alveoli (0), or in a continuous alveolar groove (1).

**Character 361** (Pol *et. al*., 2014: char. 384; modified from Andrade et al., 2011: char. 399): Left and right toothrow along mandibular symphysis: well separated from each other by a broad dorsal surface of the symphysis (0), or closely located to each other (forming a symphyseal tooth battery in most taxa) (1).

**Character 362** (Pol *et. al*., 2014: char. 385): Apico-basal ridges on the enamel surface of incisiviforms and caniniform: absent (0), or well-developed (1).

**Character 363** (Pol *et. al*., 2014: char. 386; modified from Andrade and Bertini 2008a: char. 123): Apico-basal ridges on the enamel surface of posterior teeth: absent (0), or present (1).

**Character 364** (Pol *et. al*., 2014: char. 387): Separation of apico-basal ridges on the enamel surface of teeth: fine enamel ridges that are closely spaced to each other (flutting) (0), or ridges, usually with a broad base, well spaced from each other (1).

**Character 365** (Pol *et. al*., 2014: char. 389): Thin enamel ridge (loph) connecting adjacent denticles instead of presenting distinct interdenticular slits: absent (0), or present (1).

**Character 366** (Pol *et. al*., 2014: char. 391; modified from Riff and Kellner, 2011: char 264): Posterior teeth with accessory apicobasally oriented keels bearing cusps or tuberous denticles located lingually and buccally from the major central keel: absent (0), present (1).

**Character 367** (Pol *et. al*., 2014: char. 392; modified from Turner and Sertich, 2010: char. 294): Outer enamel surface (between carinae, apicobasal ridges, or flutting, if present): smooth (0), rugose (1).

**Character 368** (Pol *et. al*., 2014: char. 393; modified from Andrade *et al*. 2011: char. 374): Rugose texture on outer enamel surface: formed by anastomizing grooves and ridges (0), formed by small globular protuberances (“pebbled enamel” *sensu* Price, 1950) closely spaced to each other (1).

**Character 369** (Pol *et. al*., 2014: char. 394; modified from Andrade and Bertini 2008a: char. 138 and O´Connor *et al*. 2010: char 235): Tooth-tooth occlusion wear facets in posterior teeth: absent (0), present (1).

**Character 370** (Pol *et. al*., 2014: char. 395): Location and orientation of tooth-tooth occlusion wear facets in posterior teeth: oriented horizontally on the occlusal surface of the crown, parallel to the longitudinal plane of the skull (0), or located mesiolingually from the apex of the crown in upper teeth and buccodistally from the apex in lower teeth (Pol 2003: fig. 3; Lecuona and Pol 2008: fig. 1), oriented along a plane that is oblique to the longitudinal and sagittal planes of the skull (1).

**Character 371** (Pol *et. al*., 2014: char. 396; Turner and Buckley, 2008: char. 290): Prominent depression on palate near alveolar margin at level of sixth or seventh alveolus: absent (0), or present (1).

**Character 372** (Pol *et. al*., 2014: char. 397; modified from Turner and Sertich, 2010: char. 293): Gap on line of large neurovascular foramina on lateral surface of maxilla, along alveolar margin: absent,foramina form single continuous row (0), or present with a gap between anterior series and posterior series of foramina (1).

**Character 373** (Pol *et. al*., 2014: char. 398; modified from Sereno and Larsson, 2009: char. 46): Lateral surface of jugal-ectopterygoid contact: inset from lateral jugal margin (0), or confluent with lateral jugal margin (1).

**Character 374** (Pol *et. al*., 2014: char. 399; modified from Montefeltro *et al*., 2011: char. 45): Ventral margin of jugal at posterior end of ectopterygoid contact: continuous with the infratemporal bar of jugal (0), or suborbital region of jugal separated by a notch from infratemporal bar of jugal (1).

**Character 375** (Pol *et. al*., 2014: char. 400; Sereno and Larsson, 2009: char. 83): Single or paired large neurovascular foramina on lateral surface of premaxilla, at its posterolateral corner: absent (0), or present (1).

**Character 376** (Pol *et. al*., 2014: char. 401; modified from Montefeltro *et al*. 2011: char. 5): + Prefrontal-prefrontal medial contact: absent (with a broad contact between nasal and frontal) (0) anterior region of prefrontals project a medial pointed process that almost touch the other prefrontal (with a tiny contact between nasal and frontal) or touch each other as a punctual contact (1), contact present along mostly of the dorsal medial edge (2).

**Character 377** (Pol *et. al*., 2014: char. 402; Montefeltro *et al*. 2011: char. 23): Maxillary palatal sagittal contact: smooth (0), bearing a longitudinal series of foramina (1).

**Character 378** (Pol *et. al*., 2014: char. 403; modified from Montefeltro *et al*. 2011: char. 33): Quadrate lateral depression: absent (0), present and elongated, reaching close to or extending into the quadratojugal-quadrate suture (1).

**Character 379** (Pol *et. al*., 2014: char. 404; Montefeltro *et al*. 2011: char 34): Periotic quadrate fenestrae on lateral surface of quadrate: visible in lateral view (0), internalized in otic notch (1).

**Character 380** (Pol *et. al*., 2014: char. 405; modified from Montefeltro *et al* 2011: char. 42): Medial palatal contact between suborbital fenestra: (0), distinctly raised forming a ridged suture along its whole extension (1).

**Character 381** (Pol *et. al*., 2014: char. 406; Montefeltro *et al*., 2011: char. 64): Posteroventral symphyseal depressions: absent (0), present (1).

**Character 382** (Pol *et. al*., 2014: char. 407; modified from Montefeltro *et al*., 2011: char. 11): Extension of frontal sagittal ridge: extending along the entire frontal dorsal surface (0), failing to reach the anterior end of the frontal, extending up to 75% of its anteroposteriorlength (1).

**Character 383** (Pol *et. al*., 2014: char. 408; modified from Montefeltro *et al*., 2011: char. 37): Supraoccipital dorsal exposure on skull roof: subtriangular or crescentic shaped with the maximum anteroposterior length located along the sagittal plane and lateral regions anteroposteriorly shorter (0), forming a anteroposteriorly short but lateromedially broad surface sutured to the posteriormost portion of parietal and squamosal, with the lateral ends as anteroposteriorly long as the central region (1).

**Character 384** (Pol *et. al*., 2014: char. 409; Larsson and Sues, 2007: char. 71): Sagittal torus on maxillary palatal shelves: absent (0), or present (1).

**Character 385** (Pol *et. al*., 2014: char. 410): Groove located on premaxillary lateral surface, running anteroventrally from the dorsoventral midpoint of its posterior margin: absent (0), or present (1).

**Character 386** (Pol *et. al*., 2014: char. 411; modified from Nascimento and Zaher 2011: char. 258 and Montefeltro *et al*. 2011: char. 16): Suture between the postorbital and the squamosal in lateral view: straight or almost straight, vertical or oblique (0), or convex anteriorly (1).

**Character 387** (Pol *et. al*., 2014: char. 412): Anterolateral corner of supratemporal fossa: with a continuous rim formed by the postorbital dorsal surface (0), or with a transversely oriented groove on dorsal surface of postorbital interrupting the anterolateral rim of the supratemporal fossa (1).

**Character 388** (**new character**): Anterior tip of mandible in lateral view: tapering anteriorly (0), or abrupt convex (1).

**Data matrix**

The following list corresponds to the data matrix used in the phylogenetic analysis. Polymorphic or uncertain scorings are given in parentheses.

*Gracilisuchus stipanicicorum*

000000?0?00000000000?0?0000000000??0?0?0000?00??0000?0???0?000-100000?0000000?000000?0?0000001012?000??????01?01000??1?01???000001002?0???0000????000???00000?000????000???0????0???????000?0000?0?000000000?0?0000?00?0?0??0?0??000??0?00??1?0?0?001???020???1??00??0??????0?????0????????????????????????????-00000???????010?0000???00000?1000?0??0?10?0?0000???000?0000??00???0?0?00?0??0??00000

*Terrestrisuchus* *gracilis*

000??00?0?000000?000?0?00?000?01?00000?0000?00?0?000?000????00-??010??00000000000000?0?000001101??010??00?00?00100??10?00?110?0?0??[01]110???00000???000???00100?000?001?00???0????0??????0????0??0?0??0?0???????????0?????????0?????0?????????????0?00?????????1???????????0??0?????[01]0??????0???0?0?0000?00?????0?00??0????????1????????0?00?0?10?0?0??0?00?0?0000???000?00?????????0???00?0??0???0000

*Dibothrosuchus* *elaphros*

000?00?20?001???000000??????0011000000?0000?00?00000?0?0102000-000100?0211000???2000?00????010010?010?0?0?000001001?10?00?1?0?0101011100??000001??0000?00?100000000010000?000??00?0?001000100100?0?000000000?000000?0?0000??0??00000??0000?00?0?0?000?0?0201?11??00??0?0110000000000????00???011000000?00????00000000?????????????????0000?001000?0??0010???0000?0?000?0000??0????????00?0??0???0000

*Protosuchus*

21000001000001101000010000010011001010?02010011110010101103011-10021000?011000010[1234]00?11001101001110201010100[01]000000?01??01??10010[01]0101000100???011000000000000000?00100000?0???0000?01002000011110??001000?010?0000?0?0000??0?000000??00000000100000????0?0?????000??000???0000?000?0????00?0?01??0000100?????0000101?000???00010100?0?000?0?000000??00??[01]0?0????????0?0000?000???000000?00?0???0000

*Hemiprotosuchus* *leali*

?10?00???????10010?0??00?0010?11???01??020?001?1100101??1?3?11-?0?21????01??????0????1100?1?1001??0????????000?000?????00???00000??10?????00???????0?0??0??00?0?????1?0000?0????0???0???2???01??10?00?00?01??00???0???0?????0?????00???00???00????00????0?0???????0??0?????00????0??????????????00??????????????????????????????????????0?????????0??0??????????????????0?????????????????0????????0

*Orthosuchus* *stormbergi*

21100001010001001000[0 1]1000001000100?000?020110111100??1?1?03011-0?0?0?000011000110000?11001001102114201?10?10?100000001?0101000001?000?0???00001??0000???101000000?0?100000?0????0??000?02?00011110?0001000?0?000?00?00?0?0??0?000?0???0000?000100?00???1020?101?0?0??00?000?0???0?00??00?????0[01]0000000?00?0?000100001?01?000??????????0?00?000100?0??0010?0?0000???000??00???0??????0000?0??0????000

*Zaraasuchus* *shepardi*

10???????????1?01?01?1000001?1-?2?????????????????????????3??-??0??010???????????[1234]0??1010??0??????????0??????????????????????0?????1??1?????????10?????????0000??????0???0?????0?????1????1?0???0???0???11111111111???????????????????0?????00????????????0???????0??0?????????????????????????????????????????????????????????????????00??0?01????????10????00000000????????????????0???????0???0??

*Gobiosuchus* *kielanae*

101000?1000011001??[01]?1?00001?1-?201000?0201121111000?0????301--?0?10100001?0?1?????0?1010110[01]10120020100???0010[01]00001000000?00001001211?0100???11000000001?00000??0020000000???00?0?01?02100011?00?00?0011111111111?000000??00000000??0000?00010??00????020??1????0??0?01[12]??0000??0?????000????0??0??0????????????????????????????????0000???110??1??00??0???000?00000??000??00???0??000000??0??0??0

*Sichuanosuchus* *shuhanensis*

[12]01??0?10000?10010?1?110???1?00?21?1010020?1?1?1100???????3?11??0?1?0000111??????000????????11?11?0????0??100100??1??10?0????0011?[01]1210??10?????1??000??00100?00?0???000?0?00???0?00110?1101111100?101000010011???0???0?00??000?0?00????00??001???00????0?0?????0?0??0?????0???000????????????????0000?0???????1???????????????????????000?0??1100???001????0??0?????0??000??00????00000??0??0?????1

*Zosuchus* *davidsoni*

201??0?10000??001010[01]110?001110?221101002?1??1?11000?0?1?03?11?0????0?0?11??????????????????11?12?3??1?????00100011011?0001?0?0010112?[01]?0101???0?0?00100010000?[01]21????0??0?00???000?100011??111?101101000000100???0?000?000?0000000???00?0?000100?00????1?0????0??0??0????0?00000?????????????????????????????????????????????????????0?00???011??1??0??????0????????0??0????00???0?0000000???0????1

*Hsisosuchus*

201?0?????000010100001100011000?21?10100[12]??121?10000?0?1?0[23]?111?0?00?02?1?10?????000?1000???0101?102?1?????01001?????0?0000??000?1011?1??001????10???0??0??0010?00???000?0?000000?00110?[01]00?0011111100[01]?0000?0000?0???000?0?10??0??????000?0001?0?00????0?0??????00??0??????0????0?????????????????????????????????????????????????????000?0001?0?1????10???001101000?????0??0??????00?0??????????00

*Uruguaysuchus* *aznarezi*

201?00?02?00??10??1??111??1???0122?1010011???1?1010??0???0[23]?11110100012210??1?2?[12][1234]000?0?0??011?110020?00??001[02]0110101?00??001?0111?0[12]??0?011???01110000101?00?0??00?0?0000?000110101?110?0?0001?10010??000???00???01[01]??00?001?00?000?0??0?????10??00????0[12]0??1?11001100?1??00?00?0?10??1??1?????????????0?????????????????????????????00?00????????00000011000???10?00?0000?000?0?00000?00?00??0???0

*Candidodon* *itapecuruense*

201??0?02?0011100010[01]110??11000?22?10100?1??21?1000?10?1?0??111???????221???????????????????11?1?01?010????010011?1?100000?0100?10001???0011???0?0?00??101?0?101?00?1?0000?0001?00001[12]100010?01?100?000000000?????0?1?0000001000?0?0?00?00?000100?00??????0??1????0000?????00000000???????????????????????????????????????????????????0?00???02???1[01]000????????????????0000?000?0?00001000?00?00??00

*Libycosuchus* *brevirostris*

201000?02?00??10?010?1???011000??2?101???1112??10?0??0???0?0111?010001?100??????????????????1002010????????01?011???????????1?011?00??1???01?????0?000??01?00?01??0???0000?0???0000?01100????01?100??01??????00???0?[12]00?????1?[01]0?000?????0??00?00?0?????0?0??1????0??0??02??0??0?0??????[01]11????????????????????????????????????????????00?????????1??0??????000101011??00????????????????0?00??0??01

*Simosuchus* *clarki*

103010100000100010111110?011001121?1010011?121?1000010?1?03011110101002000???2??2100?2010?100012010?01??????1101101212000010100111002110002011?210?0000101000101200?0?0-00[01]0?111100111100010011?100010000000010000021000000?10[01][01]0010??00000000100?0010??020?0100??0??0?011?0000?00010?1???1?10100100011000110001??????0[01]??000101120?01[01]010011021000??0110[01]1[01]0101110110?1000??00?0?00000000?00000?101

*Malawisuchus* *mwakasyungutiensis*

101?00?12000?[01]10001[01][01]1100?1100012211010011??2??1000?10?1?03?111[01]0?010022101?????2100000?0??01112111?01?0???01100101?11000???100110101?0?0001???0?01000?101000001?0??1?00?0?00001[01]?0012110?100?11100000000000000???0?[01]?0?00001000???0????00??001?0?00????0?0??1?0??01000?????0??000?1????1?1??1????0?????0??????????????????????????????100?00???0??000000[01]??0?0??????????00??00?0??0000000?00???0?00

*Pakasuchus* *kapilimai*

101??0??21??1110001011?1?011000122110100???121?1000?10?1?03?2111??01002?1???????1?0??000???0?111?13?01?????01100??????000000?001111011?000011100101000?101001?01??0?1?0?00100001100?12110010001?100?000000000000000???000?00110???????0??0?0001?0?00??????0???????0000??????00?000010???1???????????????0???????0111???????????????????10????02???00000??1??0?0?1?011????00??00?10?000?????00??????0

*Notosuchus*

101?00?020001110001111110011001122110110211121?10000?0?1103111111?01001100111?212[01]0001000??01112012?1100121[01]1201[01]010010000001111111111?0001111001010000001100001100?1?001000?1110001121001100111101100000000000000021010[01]01011[01]10000?00000?0001000000?01020101?1?000100000?0000100010?10111?1101?110010?0111?11101111?001?100??????0??000000012101[01]0010102101101100111?00011000?11000000000001000000

*Comahuesuchus* *brachybuccalis*

103??0?02?00?????0111????????011?2????1?11?1???????????????121??????0?1010???????????????????1??[01]13?1??????0000?101201[01]01?????011??0?1????11???11??020??00102001[01]0[01]?1?0?00?1011?000?11100??00???100???00000??0????0?[12]01[01]1110?1[01][01]0000?00??0?000100?00????120101????001???1[01]??0??1?0????????????????????????????????????????????????????0???0001?????[01]000??2?1??????????1?000?000?0????00000?001000??0

*Mariliasuchus*

101?00?0200011100010[12]111000110112211?111211121?1100010?1?0312111010100120011?2??2?00?0002??0?112113211001??0020[01]1010010??[01]0010[01]11110110?00111??01010000101002001[12]00?1?0100[01]0?111000112110010011?10100000000000000002[12]011111011010000?000?0?000100000????100?[01]101?00010?001??0001100?????1?????????????????????????????????????????????1110111121011001001211110110111110011100101100000000000?000000

*Caryonosuchus* *pricei*

1?1??0?0??0????????????????????????????????????????????????????????????000??????????????????????31???????????2??1?1?????????0?1111??????????????1??????10???????????1?0????0????00?????????????????????0?????????????????0????0??000??????????1?????????0?????????????????0?0????0???????????????????????????????????????????????????????????????????1???????????????0??1111?01111????0????????????0

*Sphageosaurus* *huenei*

101?00002?00??100?????110????????21101?????1?1?1100??0??????2?1????????00???????????????????0??[12]312??1???????21111101111111110111110011101111?0?10??0??100??0?0?????1?0????0????00??1?1?0?10?11??0000000?00???????0?10?010??1?00?000?00?0?????10??000???01???10???001??0010?0?0?101???????????????????????????????????????????????????0??01????2??0[01]011??????????????0?01111?0111101010?00?0???00??0

*Armadillosuchus arrudai*

101??0002?00?11000101111001100112????????111?1?11000?0????3?21?????????201??????????0101[01]????1?231???1???????2?1?11?11?1?1??00??11100?1??111???????????10000010110??1?0??1?0????0???1???0?1??1?110?0010000000??00?0???10??????????00??0?00?0001?0?0?????[01]?00??0???0?????0?0?00???01??????????????1?00???0?1????0????????????????????????0??11022??0??1????????????????0?1111?01111????00?0????0?01[01]0

*Caipirasuchus* *stenognathus*

101?00?02000?11000111111001110??22110110211121?11000?0????3011111101001100??????????????????1112112?11?????002011010110111001?1111101?010111???010?0000101000001?00?1?0100[01]0?111000112110010011?10[01]100000000000???020011101011000000?00000?000100?00????[01]00?0101??0?10?00100000100????????????????????????????????????????????????????001111?121010?011112111101101111?1111110111101011000?001?00110

*Caipirasuchus* *montealtensis*

101????020?0?11000111111?011101??2110110211?21??100??0????301111????0?1200??????????????????01?2?12?11?????00?01??1?1101[01]?00101111?01?0??111???01??0000?01000?0??00???0100?0?11?000112110010?11?100?000000000?????010?101010110000?0?00000?000100?00????0[01]0?01?0??001?????0000?1101???????????????????????????????????????????????????0?11?01121?1?[0 1]01111211?????????1?11????0??11010100000001?00010

*Caipirasuchus* *paulistanus*

101?000020?0?11000111111?011?01?22110110???1?1?????0?0????30111111010?1200??????????????????01?2112?1??????002011[01]101101[01]?001?1111101?????11???01??0000?01000??100[01]???0100?0?11?000112110010011?100?0?00000??00???010010?01011000000?00000??00100?00????010??10???0110?00100000100????????????????????????????????????????????????????0?1110?121???[01]01111211???????111?11111?0??1101010000?000?000[0 1]0

*Yacarerani* *boliviensis*

101?00?02?00?110001011110001101122110110211121??1000?0?1?03121111?00001200???????????????????112112?11?????0021110100101-100101111?01???0111???010?0000101000001000?1?0-10?0?111000112110110011?10000?000000000???020010111011000000?00000?000100?00????100??100??0110?0010?0001001??????????????????????????????????????????1011200011110?11120011001111211110110111101100?11111101000000010??00100

*Adamantinasuchus* *navae*

101??0?0??00??10?0111111??111??????????????????????????????121?1????0??100???????????????????1?1112?1??????0?2[01]0101?01?0????1?1111?01??????????01??000?101??00??????1?0????0????0????2??????0????0??0??0001??0????0?0??111????00?000??00?0????1?0?00????1?0???????0??0????0?00???0???????????????????????????????????????????????????????????????????1111?0??????????10??00?11?????1000???0?????0?00

*Campinasuchus* *dinizi*

1000?0?12?00?1101?111111001100113211011121???1?1?00????????021110?00011211111[12]21??00?0???0???1021032?1?0????101110101111000?110111102?0?0?11???[01]10?1[02]0?000?0010110[01]???0111?001100?0011100110011?100?0000?000000???0?21[12]000111110?000?00?00?000100?00????020??1????0010?00[12]0001111001???0????10??1[01]???1?0????????001[01]?????1?0???????????01????121?1?1100111??0?0?10?110?0000?000?0??0?11??10?11??10?1

*Pissarrachampsa* *sera*

100??0?12?00111010111111?011001132110110211121?10000?0????3?211???????1?11??1[0 1]21???0?????????1?2204?01??????1011?01?1111?001?10?11102?[0 1]?0011???[01]?0?1[02]0?000000101101?1?01111000[01]?0?0011100110?11?1001000000000?????0?2?10001111110000?00?00?000100?01????020?11????001????????111100??????????????????????????????????????????1?10200??0?10?00?210?11100??1?1?????????0??000?000?0?0011101101111??101

*Baurusuchus* *albertoi*

1?????????????10?????111????????3???????21??21???????????????1??0??10?????1110212100?00?20?0??02???2??001211??11???????1??01?10???1021????11110??01??0????-?0101???????1???????0????10??0?1?0??1????000?00000000000?????0?????????????00???000???0??0??1????[01]1??100??00??????????0011110011?101??1111100?011011101111?00?01??1110?000??010???12?????????????0??1?0211???????????????11???11??????1??

*Baurusuchus* *pachecoi*

100??0012?00?1101????111?0110???32?1011?211121?1000?10??10??211101010112111??????????????????102103??1?????1101110101[01]11-0011101111021[01]?0011???[01]00?1200000-00101?01?1?011??000[01]00100101001?00?1?00010000000?000???0?2??000111110?000?0000??00010??0?????020??1-???0020??110?[01]111100???????????????????????????????????????????????????00?0???1?101?1000201?10?????2110?0000?000?0?0?101?1111???01101

*Baurusuchus* *salgadoensis*

100?00?12000?110101011110011001132110110211121?1000010?1?03021110?0001121111?0?12?0000002000?102103201?012?11011101?1111-001?101[01]?102?[01]?001?110[01]0011200000-00101101?1?0111?00010010?10100?10011?000?00000000000???0?2120001111100000?000?0?00010?001?0??020?[01]1???00020?0??0?01111001??????????????????????????????????????????????????0010?011210111000201110001102110?0000?000?0?0?1012?11111101101

*Stratiotosuchus* *maxhechti*

100?000120001?101011[12]111001100113211011021?12101000010?1?030211????001[12]?1?1?1021??00???????????2203201?0?211101110101111-00?1100111[01]211?0011?1??001??00000100101101?1?0111100?100?0010100110011?100100001000000???0?2020001111100000?000?0?00010??01????020?110?1?001000110?0111100?????????1?1011111100?0110111??????0010101101[01]200??0000?01120011110020??100????2110?0000?000?0?0?11021111111?100?

*Pehuenchesuchus* *enderi*

????????????????????????????????????????????????????????????????????0?1??1???????????????????0????????????????????????????????0??1?????????????[01]0??030?1????????????????????????00???0???????????????????????????????????????????????????????????????????????????????????????????????????????????????????????????????????????????????????????????????00????????????????00????00????????????????????0

*Bergisuchus*

1?0?????????????????????????????????????????????????????????1????????????1??????????????????11???00?????????1????????0????????0001??????????????0??0[23]01??0????????????0????0????0?0??0???????????0??????????????????????????????0?????????????1?????????????????????????????1?????????????????????????????????????????????????????????????????????????0???????????????????0????????????0????????????

*Iberosuchus* *macrodon*

1?0?0001??00111000111111?01?00??2??101?111?121?1010??0?1?0?0111??10?0?101?11????[12][1234]00??00???0????[01]0?20000???1101110101??1?0??100001001?0??001???[01]000??01000100101?0101??1?100000?0[01]00001001?0?11?100[01]001000000??0??0?212000?01?10?000??00?0?0001???00????0?0???0?1?[01]???0010[01]0?0??[01]00111??????????101[01]01110?????????????1111??????????????00?0?02???1??00??1???????????0?0000?00???????100?0??01?00???

*Bretesuchus* *bonapartei*

100?00112?00??20???????0?????????2?10102???????101?????????02?1?11?1001011??????????????????00??100????????1?0??10?0100???0???0001?1???????????[01]1??1301[01]10??????????1?110??00???010??01??0?00?1??00??????????11?????212?001?1010?101?[01]?0??????10??0?????02???10???0110?00[12]1?1?0010????????????????????????????????????????????????????00???????????0000??11?[01]?????2110?0000??00???0???0????0???10??1

*Barinasuchus* *arveloi*

?00000?1??00?????????????????????2?10110???????????????????02?1???????1211???????????????????1??100?????????1?0?0???10????????0001?????????????????1?0??0?????????1???0????000??0?0??0???0?0??1??0??????????????????0?2?????10?0?000??????????1?????????????????????????????1???1?????????????????????????????????????????????????????????????????????????????????????????0??0????????0?????0???0??0

*Sebecus* *huilensis*

???????????????????????????????????????????????????????????0???????????1?0???????????????????1???????????????0??0?????????????0??2??????????????1??130???0??????????????????????0????0?????????????????????????????????????????????????????????????????????????????????????????????????????????????????????????????????????????????????0?????????????00?????????????????000?000????????????????????0

*Sebecus* *icaeorhinus*

100?0??12000?1100011[01]110???1001122?101111111211?010010?110302111110000[12]010??1?2?2400?????????1011002?0000211?0010??00001??00?00002011010??01??00110130?1?0001100[12]0??1?[01]10??00??00???00?000100011100?001?0000011???0?0?200??0101??00??000?0000010??0?1?????000000?011000[01]0011100011??1?11110101011101??00111111111101101111101111021000?0000000???010000???????1?0??1?0??000?000???1?010000?0?1??0??0

*Sebecus* *querejazus*

100?????2???1?1??011??10?????????2?10101?????????1??????????2?1?????????[01]????????????????????????00??0???????101?????101??00??0002?[01]???0???????0??????????1?[12]?????????010?[01]00???0?????1??0?0?01??00??????0????????????200?00101???????0??0????10??00?????1?????0???[01]0???????10001????????????????????????????????????????????????????????0?????????00?????????????????????0??0????0????00??0?0?0????

*Ayllusuchus* *fernandezi*

[12]01?00112?00????????????????????????????????????????????????2??????????21???????????????????0???10[012]?0???????????1??001????????001[01]???????????????????????00?????20????1????00????????????????????00???????????????0?2020?0?????0??????????????10????????????????????????12110??????????????????????????????????????????????????????????????????????00????????????????0????????????0???0?0??????10???

*Lorosuchus* *nodosus*

102012112?001?100011[12]10[01]???10?0??2?1010111???1?1??00?0?1???0211?1?01??201?????????????????????01100?????????11010?101100????0?001101[01]?1-??01???001?0?11100??0??1??1?1?01??000??00000001000?0??1?100?0010?00?000???0?002?00??1010?000?00???????10??00?????21??100???0?0?01[01]1?0000100???????????????????????????????????????????????????00?0?0???????0000??2??00110?0110?0000??00???0?000000?000?00??0

*Pabhwehshi*

??0?0011??00???????????????????????????????????????????????021????????11[12]0???????????????????1??20??????????1???10101?????????0??1?????????????[01]????????0???????????????????001?00?????????????????????0????????????20???0????11?000??????????1????0????????????????????120??????????????????????????????????????????????????????????????????????????0?????1?????????0?000??0?????????0?????[01]??10??1

*Araripesuchus* *gomesii*

201000?220001110001011111011100122110101111121?10000?0?11030111101010?201?1011211[234]000100010011011002010002101101[01]010[01]10000001001110021000?110?00111110?101002100[12]00?10100011000101[01]1101010100011100100000000000000010000000010000000?000000000100000??010?01?1?11100000?1??0000000110111[01]1[01]11010?00001100??????10111100000??1001?200??000001002000100000011?0011010100?0000?000?0??0001000?0??000000

*Araripesuchus* *wegeneri*

201??0122100111000111111?011000122110101111121?10000?0?1?030111???0???221????????????10001?011?1000??1??????10011010110000001001110021[01]00011???01???10?10100110020??1?10001100??000010101010?01?1001001000000??0000?1000000010000000?000000000100?00????020?0110??000??011000000001???????????????000?????1????1??????????????????????0?0000002000100?0??1???????????0???[01]0?000?0000001000?0?0000001

*Anatosuchus minor*

203000?021001110?011111??01100012211010?11???1?10000?0?1?0?011111?000120101?????[01]?00?10001?01101000?01??1?1?1101001?100000????0110002?[01]??01?0???10?0?0??0?00210?20????1000[01]1010?0?00101010100011100[01]000?0000???0000?0?00000011?00?10?0??00000010??000??1??0?01?0?1?000?????000?0000?????????10???????1??0?????????????????????????????000??000200010?00??1??0011010100??000??00????0001000?001?00001

*Montealtosuchus* *arrudacamposi*

201??0012100111000100111001110112211010111112111010010?1?0301111110000121111????1[234]00?10?0?1011011002010???1110011010100000001000110011100?01??0011?1301100001100000?1?0000100010000000100010001?100000[01]110000010??0??0000000100?0000?200000000100?00???100010110??0100?111100000000?????????1010?0??0?1????????1??????????????????????000000002001100001111[0 1]0011010100?0000?000???1?001000?00??00001

*Uberabasuchus* *terrificus*

201000?1?100??1000101111001100012211????11???1????????????3011?11?01001211???1?????0?1020??0010200020??0??111[01]01101?10000????00011001?1???0????001?1?0??0?00110000???00??0?100100?0??0????1?0??1100?00?110000010??0?2?0000????010000??0000?0001?0?00??????0???????0??0?1??1000???01?????111????0????????0??????1???????????????????????00??0002????0?0011???001101?100??000??00?0???000??0??0???0101

*Lomasuchus*

201????12100111000101111?01100112211010111?121?10100?0?1?03?111????000??11??????????????????1?01?00?0100?1?0?001??10100000???00011001110000?????01??3011000021???0??1?1000?[01]0??00?0000100010001?100000111000001???0???000000100?0?00?200000000100?00?????101???0??0100??1???0000001???????1?1?1000000??0????????????11?0????101102????000000002?01100??11????0110??10?????0?00????1?00?00??0???0?00?

*Gasparinisuchus* *peirosauroides*

203?00?12?00??????10[01]1???????00??2?10???????????0????????????1??????0??211???????????????????10?000?01??????10011?10100?????0?0010?????????????[01]01?13?1?000?????[02]0??1?10???[01]0??0000??0?????0?????000???1??????????0???0?00?0??0?0000????????0010???0??????????1????0???1011?0??????????????????????????????????????????????????????????0?????????????00??????0??0??1?0??0?0?00????1????????????0???1

*Hamadasuchus* *rebouli*

201000112000111000101111?01100011211010111112111010010?11?[23]0111???????[12]221??????????????????11?1100?01??????10011011100100000000110011100001???[01][01]1?130?10000110000??1?10001100??000000100010?01?1000001000000?????0?0100000010010000?200000000100?00?????10?01[01]0??010??111100000001???????????????????????????????????????????????????1?000000200?100????????????????0???00??0????1?001000??0??1?000

*Mahajangasuchus* *insignis*

103?1[12]?021?01211?01011110011001142110101011?21?10100101110??1111110101?0111?122?140000000??01102[012]00?0100?11111010?121?00?000000011102110000????20111300100002100[12]00?1?[01]1011[01]0??0000100100010001110000010?0000000??020?0000001?000000???000000010??01?????11?[01]??0?10100??1?01000001011110011?10???0000110[01]001110?01111110101[01]??????00??0000?0???10110000?????0011000100??000??0100?0?110000?1??0???01

*Kaprosuchus* *saharicus*

1031121121001?10?0111?11??11001142110101?1???1?10?0???????3011101??0011121??????????????????1002200?0??????[01]?10?0?1201000000?00011002?1??00????[12]11?[01]300?00001100?0????1100110??00?0100100011001?100100???00?0?0???0[12]01000000100?0000?[01]0000?000100?01????010??1-1??00?0?10211000001????????????????????????????????????????????????????0?00?00021??100?020[0 1]??0011010100?0000??0??0?0?110000???0?01?01

*Stolokrosuchus* *lapparenti*

201000012000111[01]00101111?00100111211????111121?1010010?1?0?011?1????00?21???????????????????1??1000?00??????1101111011010000000010001?101101?????1???11100002101000?1?1??0[01]0100?000?00?00?1000??10000010000000????0?010000????000000?10?100000?00?00?????2010?1???01???11011-00?001???????????????????????????????????????????????????0?0000?0200010000??10?0????????0?00010?0????0??000?0????01000?

*Theriosuchus*

20310111200011000011011110011001?21101001?11?111000?????1?20111??100102011101[12]210[234]1021100100110100020?10??101[01]0[01]001?1100?00?0?00110??01??0?00??10[01]00210100[0 1]02?00?00?1?10001110[01]?0??0?010?010?01?1000?0??0000?2????0?0?0???0?10??0000?0???000??100?00??0?0011?1??0?000?0?????00?0?00???????????????????????????????????????????????????0?????00?0?010000??????????????0??0????0????0?00?0?0?0???0??00

*Goniopholis* *simus*

203?12112010111000100111?001001102?10100?11121?1010?10?1?021211??10010?0211?1???0?00?1100?11?10?00020010??1[01]11010-101100?000010010001?1???0000111000311000102110010?101000111??00000001000100011110?001000000000000?0000000?10000[01]00?000?0?000100?00?????00????????100??0???000000???????????????????????????????????????????????????????0?0?0?????00????????????????0??0110?0??????0?1????0????1?0?

*Eutretauranosuchus* *delfsi*

203?????10?0111000100111?00?000101110?00?11121?1010??0?1?0??21101?00002?2011????0??0?1???????1??000?00?????0?101????110???????0?100???1???0????110?0311??010211001????1000??1???000000100??00?1?110??01?0000000???0?000?00??100?0?00??00?0??00100?00??0??2010??0??0100??????00?0001???????????????????????????????????????????????????0??00??0????10??0??????????????0??0?0??0????0?001000????????01

*Calsoyasuchus* *valliceps*

203?020[01]1010?110?01021111001000?01?1???0?11121??0?0??0?1?0?0111????????21???????????????????0???000?00???????1010-11110?0???0?0010001?1???00????????????001021?00?????1????01???0??00???????????1100?0100000??????0?0000000???000000??0?100?0?100?00??????010??0??0?????0[12]??-?0???????????????????????????????????????????????????????????????????00??????????????????????????????0?0?????????????0?

*Sunosuchus*

203?02011110?110001001111001000102210100111121110100?0?11021211??100012011??121??20001100?11?1??????????????????????????????????????????????????????????????????????????????????????????????????????????????????????????????????????????????????????10????011???000100?001??-0????0?????????????????????1???????0?????????????????????????????????????0?????????????????0???????????0???????0??????1

*Shamosuchus* *djadochtaensis*

203????0?1?011100011011100010001021101?11111?111010000?110??210?1100102?1??????11[34]1?[01]20?0?10?100[012]00200[01]????01100??1?11?0?0000000100011101?00001??0?0?10?000021[01]??1??1?1000?100000?0?00100010?01?10?0001000000210?10???000?0?100?0??0??00?000001?0?000??1??1101??0011101?????00?0?0?0???????????????????????????????????????????????????000000020101000011????0010?0010????0??0????0?000000?????0??0?

*Hylaeochampsa* *vectiana*

00??????21??11????1?01???0????1?02?1?102????2??101??1??1????210??????????????????????????????????????0???????10????????????0??0??????0???1???????????????00021?00??????00?1?2???0?????????????????????????????????0???????0?1???0???????????00??0????????0110??????00????????00000????????????????????????????????????????????????????0?00??0?00[01]?1[01]0?????????????????????????????0?0??????0??00??0?

*Boverisuchus* *vorax*

200?02?120001110001011110001001102110??2111121?10100?0?1?1?1210?????0?201?1?122??311?1?????1?1?0000?001?????10010-1?1100-00000001100101001000??[01]???0300100002??001001?[0 1]001?1?00?000?00100010??111000001000000??0??0?0000000?10000000?00??00000100?0000110011111???0???0?00??000000???????????????????????????????????????????????????????????????????????????????????0????0?000?????0??????????????0

*Gavialis* *gangeticus*

212?1211201111110110111111000001021101020111211101101011111121002100013201111221131111021100?100000200100001?1010-12-100-00000001000101-1100001-1000010000102110[02]1?0100000[12]1??00000?0010001000111000001?00000000000?000??00?10001000?[13]00?0000010001011100010110?0000010001??-00000100?01000?0??0????????1???????0???1????????00011011100000001001000000[01][01]00?0011000000?0000??0??0?0?000000?00?000100

*Crocodylus*

2030121120[01]01110001021111001000102110?021111211101001011111121002100010021011221131112021100?100000200100001?10101121100-000000010001010110000111000310100102110[02]100111000211?0000000010001000111000001000000000000?0000000?10000000??00?0000010000010110011111?0000000001??00000000????000000000000001010001001000010000001000011011100000000001010000[01][01]00?0011010100?0000??0??0?0?000000?00?000000

*Diplocynodon*

203?12102010111000101111?001000102110?02111121110100?0?110?12100?100010020??1221131112021110?1000002001????1110101111[01]00-?0000001000101?110000111000310?00102100210?1?1000?11?00000?001000100?11100?0010?00000000?0?0000000?100?00???000?00000100?0000110201111?0001100?????00???0???????????????????????????????????????????????????????????????????????????????????0???????0????0?0??????????????1

*Alligator*

203112?02?001110001021111001000?021101021111211101001011111021101100010021011221131112021100?100000200100001110101111000-00000001000[12]1101100001110003101001021000100111000211?0000000010001000111000001000000000000?0000000?10000000?000?000001000000011021111100001100001??000000100001000000000000001010001001000010000001000011011100000000101010000[01][01]00?0011010000?0000??0??0?0?000000?00??00001

*Pelagosaurus* *typus*

202?[01]11120110201010000000000001102110100000111?1001001?10001101111000032001101??00000110001111011002001???01?1010-10-100000?000010101100?[01]00???-10000??000110100000?100200?0??0?0?000010101000110000001??0000000??0???20?00?10[01]000000310001100110?00?????200?1????0000??02??-00?001???0???????00?1??????????????1[01]00??????????????????0000?00?00?000?0010???0????????0?0?????0????0?0000?0?0???00001

*Steneosaurus*

[012]02?[01]11?20110201000000100000001102110100?00111?1001011?1?00110101?000?30001101110000?110001[01]11011?0200[01]000001101?-10-10000??0?0010?1??00?000001-0000010000110?000001100200?0?00000000010[12]0100[01]1100010010?0000000000?0020?00?100000000?1000010?0[01]0100??00?200?11?0?00000??1??-00??010000010000?00?1?????????????11000???0?001??????????00???0???010?0?00?????00110?0000??0110?0????0?0??0?0?0??????01

*Metriorhynchus*

0?2?1??0?011?20?0010?010??00????02?10100?0??11?????0?????0?1101????010?000??????????????????0101??0?20??????1101?-1?-1?0??????0010?????0??0????-????0???00110??0??????0??0?0?0??0?00?0??1?????1?00??001????0??????0???2?????1?0?00?01???111?110?0?00??????0????????0?0??????-00?00??????????????????????????????????????????????????????????????1?00000??????????????????????0??????0?0???????????00

*Cricosaurus*

002012?02011120100100010000000110211010000?111?10010[01]1?1?000100-2100103000??????0000????????01012?0?2010???11101?-10-1001???010010?[01]2?00??00???-?0?0010000110?0000011?0??0[01]0?00?000?00??1?100?1?000?0010?000000???0?0020000???00000003111111110?1?00????0?0???????0000?001?--00??0??????????0??????????????????1??????????????????????000000??001000000[02]0????0110??100?0000??00???0?0?0000?0???00?00

*Dakosausus* *andiniensis*

001????02011?200001000100000000?02?1010000??11????10???1?000100-110010?000??????????????????00012?0?20?????11001?-1?-1?01???010011112?00??000010?0?00??000110?00?0??1?0??0?0????0???00??[0 1]?1?001?00??00???000?0????0???2000??1????0001?111111110?1?00????0?0???????0?00??????-00??01?????????????????????????????????????????????????????00?0??0??0000?011?????00?????0?0000??00?0?0?0000?0??0???010?

*Hyposaurus*

?02?12?02?1???????1????0?101????02???????1?121?101??10?1?1?120???1010?3000??112???00?????????101??0??00?????110??-??-?????????0?1001??00??00???-?1000100?0????00??0?1??????0?000000?00??0????????0???01?????0?????0???????????2???????????0???0????1????0?0?1?????0??0??????-00??0???????????????????????????????????????????????????????????????01???????????????????????1[0 1]?0??????????????0??????0

*Pholidosaurus*

212?12102?1??1110?10011??001000112110101011121?101??10?100?1211111000?3000??1?2?0??0??100????1?1??0?00???????1?0?-1?110?????0?00100????0??00???-??000?0000102?100???1?0000?0??0000?00?100?10001?100?0010??0000?0??0?010?000?10?01?01?00??00000100?00??????0?????????????????-00??01???????????????????????????????????????????????????0000?0?000100??0011?0?0011000000?0000?????0?0?000000?00???0000

*Sarcosuchus*

203?12?020101?10001001100101000112?1010101?121?10100?0?100?1211111?00032011?112??[01]00?1100?00?101010?00[01]00????101?-121100??00000010010?10??00???-10?0[03]11010[01]021[01]0000010000000?000000?001000100?1?100?0010?00000000?0?0100000?10001111?000?0?000100?10????020?1100?00100?001??00000011000?100?????????????????????0?????????????????????0000?0010?1?000001100?0011000000?0000??0??0?0?000000?00??00001

*Terminonaris* *browni*

202?[01]2?02010?11??01001???101001112?10101?????1?1010??0??00?1201111000?320011112?0000?1100?10?10?01020?100?0?1101?-1?110??0??0?0010????10??00001-100?[03]???101[01]2110????1?0000?0?0000?0000?000?0??11100?00?0??00???00?0?0100000?10201111?1?0?0?00?100??00???0?0111?00000000001??-00??0?????????????????????????????????????????????????????????0?0??????00???????011000??0?00110?0??0???0?00?0?00??00001

*Rugosuchus* *nonganensis*

203??[12]??2000??1??0110111????000112110?01?11121?1010000?1?0?121??110010201????????????2?[12]0??0?10?000??0??????1?01?-1?1000?0?00??0100?1?1???00???-00?031?100??2???010???10???110000?0?0???0?10001?100?0010?000020???0?0?00000?1?0??000?000?00000100?01????0??????????111??????000??0??????????????????????????????????????????????????????0????0???11?00???????0????0??0?00?1??0??0???????00?00???0??1

*Bayomesasuchus* *hernandezi*

20[01]??????????????????1????1?????22??0????????????????????????1??1?01??2??1???????????????????10??0??????????10????????0???00??00110????????????011?1??11?[01]?0?1??????1???0????010000??????0?0?????0?????????0??1???0???????????0??????????????01?????????0??????????1??????????????????????????????????????????????????????????????????000????0[12]????0000??????0????01???0000?00????1??????????????001

*Itasuchus* *jesuinoi*

1?????????????100?????11????????????????11?????????????????????0????0?2??1???????????????????10??1????00???1110??????????????00??0?00?1???0????-11113111????[12]?0?????1????????00?000??0???????????????????????????????????????????????????????????????????????????????0????????????????????0????0????????????????0000??000????????????????????????????00?????????????????011000100?0?????????0???????

*Pepesuchus* *deiseae*

201????120?0?????01111???01?000102110101?1??2??1????????????110???????2011??????????????????010?010?0??????11101001??100-?????001[01]???1?0???????-1??13111?0?0????00??1?1?01?1100?000??0??????????10????????????????0???0?0?????0???????????????1?1?00????0[12]0????????1[01]?11???1-0????????????0?????00?00[01]?0???????????????????????????????????0?0???????00??????????????0?0011000100?0??????0??0?????00

*Roxochampsa* *paulistanus*

??????????????????????????????????????????????????????????????????????2??????????????????????1???1?????????111????????????????0??0?????????????-???13111????????????1????????00?000??0????????????????????????????????????????????????????????????????????????????????????????????????????????????????????????????????????????????????????????????????0?????????????????011000100?0?????????0???????

*Sahitisuchus* *fluminensis*

100000112000??2000110111101100012211010111??2111011001?01030111-1100102211????????0?????????1101100?010????11001101100000?00?1000101[1 2]?10?001?10[01][01]1?13011001011[01]0201-1?1101?1?0000???021?101000??100?100??000011???0????000000?0??????00??000001?1?0?????0[12]0?110???0100?0011000000000[01]?????????????????????????????????????????????????000??11011011000011???0011010000?00000000?0??????0?0??1?0?1000

*Caririsuchus* *camposi*

?01000?1?000?100?01011?0?01110011????????1?????1??????????[23]011?0??000??211?0?1?1?????10[12]1110?1?1110?0?0??????[01]0?0????1?0-????0001??00??0??0????0????3??1??00[01]?0?2????01??1?1???00????0????1?0???100?00?000000000110?000000????000?????0??0?0000?1?01????0?????????0??0????1-00???00?????????????????????????????????????????????????????0??0??0??????0?20???000??????0?00???????0?????00?0????0?100?

*Barreirosuchus* *franciscoi*

20[13]??????[01]????1000101111?011000142110101?1??21?1010000?1?0[23]?210?????????????????????????????????????0000??????01??????????01?10????10110?001?????[01]?????1?0[01]011010[01]?????000??????0???0?100010?0??100?001??0000?????0???00000011??0?????0??0?000??1?0??????[01]0?10????011??????-?000000????????1??????????????????????????????????????????0?00?0?1010?1000???????????????0??????????0??????0?0?0?????00?

**Autapomorphies and Synapomorphies common to 225 MLT’s**

List with the characters that support each clade from the consensus cladogram based on 225 MLT’s.

*Gracilisuchus stipanicicorum*:

All trees:

**No autapomorphies.**

*Terrestrisuchus* *gracilis*:

All trees:

**Char. 344:** 1 --> 0

*Dibothrosuchus* *elaphros*:

All trees:

**Char. 13:** 0 --> 1

**Char. 72:** 0 --> 2

**Char. 73:** 0 --> 1

**Char. 81:** 0 --> 2

**Char. 287:** 0 --> 1

Some trees:

**Char. 31:** 0 --> 1

**Char. 94:** 1 --> 0

**Char. 115:** 0 --> 1

**Char. 128:** 0 --> 1

**Char. 187:** 0 --> 1

*Protosuchus*:

All trees:

**Char. 15:** 0 --> 1

**Char. 125:** 0 --> 1

**Char. 128:** 0 --> 1

**Char. 199:** 0 --> 1

*Hemiprotosuchus* *leali*:

All trees:

**No autapomorphies.**

*Orthosuchus* *stormbergi*:

All trees:

**Char. 10:** 0 --> 1

**Char. 96:** 1 --> 2

**Char. 99:** 0 --> 4

**Char. 121:** 0 --> 1

**Char. 132:** 1 --> 0

**Char. 153:** 0 --> 1

**Char. 199:** 0 --> 1

**Char. 254:** 1 --> 0

**Char. 265:** 1 --> 0

**Char. 312:** 0 --> 1

*Zaraasuchus* *shepardi*:

All trees:

**Char. 187:** 0 --> 1

*Gobiosuchus* *kielanae*:

All trees:

**Char. 334:** 0 --> 1

*Sichuanosuchus* *shuhanensis*:

All trees:

**Char. 20:** 0 --> 1

**Char. 128:** 0 --> 1

**Char. 193:** 1 --> 0

**Char. 206:** 0 --> 1

**Char. 207:** 0 --> 1

*Zosuchus* *davidsoni*:

All trees:

**Char. 30:** 0 --> 1

**Char. 97:** 1 --> 2

**Char. 99:** 0 --> 3

**Char. 150:** 0 --> 1

**Char. 154:** 0 --> 1

**Char. 162:** 0 --> 1

**Char. 249:** 0 --> 1

Some trees:

**Char. 155:** 1 --> 0

*Hsisosuchus*:

All trees:

**Char. 14:** 1 --> 0

**Char. 93:** 1 --> 0

**Char. 110:** 1 --> 0

**Char. 118:** 1 --> 0

**Char. 194:** 0 --> 1

*Uruguaysuchus* *aznarezi*:

All trees:

**Char. 50:** 0 --> 1

**Char. 146:** 0 --> 1

**Char. 165:** 1 --> 0

**Char. 178:** 0 --> 1

**Char. 180:** 0 --> 1

**Char. 260:** 0 --> 1

**Char. 261:** 0 --> 1

*Candidodon* *itapecuruense*:

All trees:

**Char. 24:** 1 --> 0

**Char. 99:** 0 --> 1

**Char. 130:** 1 --> 0

*Libycosuchus* *brevirostris*:

All trees:

**Char. 72:** 2 --> 1

**Char. 73:** 1 --> 0

**Char. 94:** 1 --> 0

**Char. 265:** 1 --> 0

**Char. 266:** 1 --> 2

Some trees:

**Char. 97:** 1 --> 0

**Char. 388:** 0 --> 1

*Simosuchus* *clarki*:

All trees:

**Char. 5:** 0 --> 1

**Char. 9:** 2 --> 0

**Char. 14:** 1 --> 0

**Char. 15:** 1 --> 0

**Char. 24:** 1 --> 0

**Char. 34:** 2 --> 1

**Char. 72:** 2 --> 0

**Char. 73:** 1 --> 0

**Char. 86:** 01 --> 2

**Char. 88:** 0 --> 1

**Char. 94:** 1 --> 0

**Char. 116:** 0 --> 2

**Char. 118:** 1 --> 2

**Char. 123:** 0 --> 1

**Char. 140:** 1 --> 0

**Char. 144:** 0 --> 2

**Char. 165:** 1 --> 0

**Char. 197:** 0 --> 1

**Char. 206:** 0 --> 1

**Char. 227:** 0 --> 1

**Char. 245:** 0 --> 1

**Char. 332:** 0 --> 1

**Char. 333:** 0 --> 1

**Char. 343:** 0 --> 1

**Char. 360:** 0 --> 1

**Char. 386:** 0 --> 1

Some trees:

**Char. 93:** 1 --> 0

**Char. 135:** 0 --> 1

*Malawisuchus* *mwakasyungutiensis*:

All trees:

**Char. 24:** 1 --> 0

**Char. 130:** 1 --> 0

**Char. 260:** 0 --> 1

*Pakasuchus* *kapilimai*:

All trees:

**Char. 10:** 0 --> 1

**Char. 61:** 1 --> 2

**Char. 157:** 0 --> 1

**Char. 222:** 0 --> 1

**Char. 369:** 0 --> 1

Some trees:

**Char. 96:** 2 --> 1

*Notosuchus*:

All trees:

**Char. 61:** 2 --> 1

**Char. 72:** 2 --> 1

**Char. 132:** 0 --> 1

**Char. 169:** 0 --> 1

**Char. 266:** 1 --> 0

**Char. 329:** 1 --> 0

**Char. 348:** 1 --> 0

*Comahuesuchus* *brachybuccalis*:

All trees:

**Char. 72:** 2 --> 0

**Char. 116:** 0 --> 2

**Char. 144:** 0 --> 1

**Char. 157:** 0 --> 2

**Char. 172:** 0 --> 1

Some trees:

**Char. 218:** 0 --> 1

**Char. 249:** 0 --> 1

*Mariliasuchus*:

All trees:

**Char. 27:** 1 --> 0

**Char. 40:** 0 --> 1

**Char. 89:** 0 --> 2

**Char. 157:** 0 --> 2

**Char. 344:** 1 --> 0

Some trees:

**Char. 20:** 1 --> 0

**Char. 216:** 0 --> 1

**Char. 218:** 0 --> 1

**Char. 249:** 0 --> 1

**Char. 273:** 0 --> 1

**Char. 327:** 0 --> 1

*Caryonosuchus* *pricei*:

All trees:

**No autapomorphies.**

*Sphageosaurus* *huenei*:

All trees:

**No autapomorphies.**

*Armadillosuchus arrudai*:

All trees:

**Char. 74:** 0 --> 1

Some trees:

**Char. 198:** 0 --> 1

*Caipirasuchus* *stenognathus*:

All trees:

**Char. 72:** 2 --> 1

**Char. 375:** 0 --> 1

Some trees:

**Char. 196:** 0 --> 1

**Char. 216:** 0 --> 1

*Caipirasuchus* *montealtensis*:

Some trees:

**Char. 273:** 0 --> 1

*Caipirasuchus* *paulistanus*:

All trees:

**Char. 382:** 1 --> 0

Some trees:

**Char. 260:** 0 --> 1

*Yacarerani* *boliviensis*:

All trees:

**Char. 27:** 1 --> 0

Some trees:

**Char. 20:** 1 --> 0

*Adamantinasuchus* *navae*:

All trees:

**Char. 72:** 2 --> 1

**Char. 96:** 2 --> 1

**Char. 112:** 1 --> 0

**Char. 203:** 0 --> 1

**Char. 347:** 1 --> 0

Some trees:

**Char. 120:** 1 --> 0

**Char. 216:** 0 --> 1

*Campinasuchus* *dinizi*:

All trees:

**Char. 40:** 0 --> 1

**Char. 265:** 1 --> 0

**Char. 306:** 1 --> 0

**Char. 314:** 0 --> 1

**Char. 345:** 0 --> 1

*Pissarrachampsa* *sera*:

All trees:

**Char. 99:** 3 --> 4

*Baurusuchus* *albertoi*:

Some trees:

**Char. 319:** 0 --> 1

*Baurusuchus* *pachecoi*:

All trees:

**No autapomorphies.**

*Baurusuchus* *salgadoensis*:

All trees:

**No autapomorphies.**

*Stratiotosuchus* *maxhechti*:

All trees:

**Char. 128:** 1 --> 0

**Char. 201:** 0 --> 1

**Char. 329:** 1 --> 0

**Char. 336:** 1 --> 0

**Char. 375:** 1 --> 0

Some trees:

**Char. 386:** 1 --> 0

*Pehuenchesuchus* *enderi*:

Some trees:

**Char. 94:** 1 --> 0

*Bergisuchus*:

All trees:

**No autapomorphies.**

*Iberosuchus* *macrodon*:

Some trees:

**Char. 7:** 1 --> 0

**Char. 24:** 0 --> 1

**Char. 111:** 0 --> 1

**Char. 125:** 0 --> 1

**Char. 132:** 1 --> 0

**Char. 135:** 1 --> 0

**Char. 146:** 1 --> 0

**Char. 152:** 1 --> 0

**Char. 186:** 0 --> 1

**Char. 190:** 0 --> 1

**Char. 213:** 0 --> 2

*Bretesuchus* *bonapartei*:

All trees:

**Char. 40:** 1 --> 2

**Char. 93:** 1 --> 0

**Char. 94:** 1 --> 0

**Char. 153:** 0 --> 1

**Char. 178:** 0 --> 1

**Char. 213:** 0 --> 2

**Char. 219:** 0 --> 1

**Char. 226:** 0 --> 1

**Char. 228:** 0 --> 1

**Char. 261:** 0 --> 1

**Char. 355:** 0 --> 2

**Char. 384:** 0 --> 1

**Char. 388:** 0 --> 1

*Barinasuchus* *arveloi*:

All trees:

**Char. 40:** 1 --> 0

**Char. 72:** 0 --> 2

*Sebecus* *huilensis*:

All trees:

**Char. 72:** 0 --> 1

*Sebecus* *icaeorhinus*:

All trees:

**No autapomorphies.**

*Sebecus* *querejazus*:

All trees:

**Char. 130:** 1 --> 2

*Ayllusuchus* *fernandezi*:

All trees:

**Char. 161:** 0 --> 2

**Char. 215:** 0 --> 2

*Lorosuchus* *nodosus*:

All trees:

**Char. 3:** 0 --> 2

**Char. 23:** 1 --> 0

**Char. 120:** 1 --> 0

**Char. 129:** 0 --> 1

**Char. 269:** 1 --> 0

Some trees:

**Char. 5:** 0 --> 1

**Char. 6:** 0 --> 2

**Char. 71:** 1 --> 2

**Char. 150:** 0 --> 1

**Char. 214:** 1 --> 0

**Char. 251:** 0 --> 1

**Char. 346:** 1 --> 2

**Char. 374:** 1 --> 0

*Pabhwehshi*:

All trees:

**Char. 72:** 2 --> 1

**Char. 266:** 1 --> 2

**Char. 384:** 0 --> 1

*Araripesuchus* *gomesii*:

All trees:

**Char. 29:** 0 --> 1

**Char. 68:** 0 --> 1

**Char. 146:** 0 --> 1

**Char. 148:** 0 --> 1

**Char. 178:** 0 --> 1

**Char. 180:** 0 --> 1

**Char. 332:** 0 --> 1

*Araripesuchus* *wegeneri*:

All trees:

**Char. 110:** 1 --> 0

**Char. 157:** 2 --> 1

**Char. 199:** 0 --> 1

*Anatosuchus minor*:

All trees:

**Char. 3:** 1 --> 3

**Char. 113:** 1 --> 0

**Char. 118:** 1 --> 0

**Char. 130:** 1 --> 0

**Char. 174:** 0 --> 1

**Char. 222:** 0 --> 1

**Char. 227:** 0 --> 1

**Char. 382:** 0 --> 1

*Montealtosuchus* *arrudacamposi*:

All trees:

**Char. 21:** 1 --> 0

**Char. 29:** 0 --> 1

**Char. 167:** 1 --> 0

**Char. 250:** 1 --> 0

**Char. 275:** 1 --> 0

*Uberabasuchus* *terrificus*:

All trees:

**Char. 167:** 1 --> 0

*Lomasuchus*:

All trees:

**Char. 108:** 1 --> 0

**Char. 145:** 1 --> 0

**Char. 157:** 1 --> 2

*Gasparinisuchus* *peirosauroides*:

All trees:

**Char. 3:** 1 --> 3

**Char. 130:** 1 --> 0

*Hamadasuchus* *rebouli*:

All trees:

**Char. 33:** 2 --> 1

**Char. 73:** 1 --> 2

**Char. 116:** 0 --> 1

**Char. 120:** 0 --> 1

**Char. 327:** 0 --> 1

**Char. 384:** 0 --> 1

*Mahajangasuchus* *insignis*:

All trees:

**Char. 8:** 1 --> 0

**Char. 16:** 0 --> 1

**Char. 68:** 0 --> 1

**Char. 131:** 0 --> 1

**Char. 145:** 1 --> 0

**Char. 157:** 1 --> 2

**Char. 251:** 0 --> 1

**Char. 267:** 1 --> 0

**Char. 354:** 1 --> 0

*Kaprosuchus* *saharicus*:

All trees:

**Char. 64:** 1 --> 0

**Char. 73:** 1 --> 2

**Char. 94:** 1 --> 0

**Char. 117:** 1 --> 0

**Char. 188:** 0 --> 1

**Char. 196:** 0 --> 1

**Char. 256:** 0 --> 1

**Char. 260:** 1 --> 0

*Stolokrosuchus* *lapparenti*:

All trees:

**Char. 7:** 1 --> 0

**Char. 27:** 1 --> 0

**Char. 31:** 0 --> 1

**Char. 97:** 1 --> 0

**Char. 114:** 0 --> 1

**Char. 120:** 0 --> 1

**Char. 138:** 0 --> 1

**Char. 157:** 1 --> 2

**Char. 275:** 0 --> 1

*Theriosuchus*:

All trees:

**Char. 6:** 2 --> 1

**Char. 15:** 1 --> 0

**Char. 20:** 0 --> 1

**Char. 29:** 0 --> 1

**Char. 50:** 1 --> 0

**Char. 60:** 1 --> 0

**Char. 61:** 2 --> 1

**Char. 69:** 0 --> 1

**Char. 76:** 1 --> 0

**Char. 91:** 1 --> 0

**Char. 149:** 3 --> 2

*Goniopholis* *simus*:

All trees:

**Char. 5:** 0 --> 1

**Char. 31:** 0 --> 1

**Char. 69:** 0 --> 1

**Char. 73:** 1 --> 2

**Char. 363:** 0 --> 1

*Eutretauranosuchus* *delfsi*:

All trees:

**Char. 73**: 1 --> 2

*Calsoyasuchus* *valliceps*:

All trees:

**Char. 21:** 0 --> 2

**Char. 61:** 2 --> 1

**Char. 339:** 1 --> 0

*Sunosuchus*:

All trees:

**Char. 10:** 0 --> 1

**Char. 35:** 1 --> 2

**Char. 70:** 0 --> 1

*Shamosuchus* *djadochtaensis*:

All trees:

**Char. 10:** 0 --> 1

**Char. 112:** 1 --> 0

**Char. 173:** 1 --> 0

**Char. 207:** 0 --> 1

*Hylaeochampsa* *vectiana*:

All trees:

**Char. 1:** 2 --> 0

**Char. 10:** 0 --> 1

**Char. 253:** 1 --> 0

*Boverisuchus* *vorax*:

All trees:

**Char. 21:** 0 --> 1

**Char. 110:** 1 --> 0

*Gavialis* *gangeticus*:

All trees:

**Char. 2:** 0 --> 1

**Char. 3:** 3 --> 2

**Char. 12:** 0 --> 1

**Char. 16:** 0 --> 1

**Char. 18:** 0 --> 1

**Char. 26:** 0 --> 1

**Char. 28:** 1 --> 0

**Char. 41:** 1 --> 0

**Char. 51:** 0 --> 1

**Char. 71:** 0 --> 3

**Char. 72:** 0 --> 2

**Char. 73:** 2 --> 0

**Char. 86:** 2 --> 1

**Char. 149:** 3 --> 0

**Char. 152:** 1 --> 0

**Char. 167:** 1 --> 0

**Char. 225:** 0 --> 1

**Char. 243:** 0 --> 1

**Char. 246:** 0 --> 1

**Char. 248:** 1 --> 0

**Char. 252:** 1 --> 0

**Char. 255:** 1 --> 0

**Char. 262:** 0 --> 1

**Char. 334:** 0 --> 1

**Char. 339:** 1 --> 0

**Char. 354:** 1 --> 0

**Char. 386:** 0 --> 1

*Crocodylus*:

All trees:

**Char. 356:** 0 --> 1

*Diplocynodon*:

All trees:

**Char. 74:** 1 --> 0

**Char. 161:** 0 --> 2

**Char. 251:** 1 --> 0

*Alligator*:

All trees:

**Char. 60:** 1 --> 0

**Char. 63:** 0 --> 1

**Char. 118:** 1 --> 0

**Char. 134:** 0 --> 1

**Char. 335:** 0 --> 1

*Pelagosaurus* *typus*:

All trees:

**Char. 18:** 0 --> 1

**Char. 23:** 1 --> 0

**Char. 53:** 1 --> 0

**Char. 72:** 0 --> 2

**Char. 132:** 1 --> 0

**Char. 239:** 0 --> 1

**Char. 266:** 1 --> 2

*Steneosaurus*:

All trees:

**Char. 145:** 1 --> 0

**Char. 196:** 0 --> 1

*Metriorhynchus*:

All trees:

**No autapomorphies.**

*Cricosaurus*:

All trees:

**Char. 65:** 1 --> 2

**Char. 344:** 1 --> 02

*Dakosausus* *andiniensis*:

All trees:

**Char. 3:** 2 --> 1

**Char. 94:** 1 --> 0

**Char. 110:** 1 --> 0

**Char. 130:** 0 --> 1

**Char. 351:** 1 --> 0

**Char. 352:** 1 --> 0

*Hyposaurus*:

All trees:

**Char. 26:** 0 --> 1

**Char. 58:** 0 --> 1

**Char. 68:** 0 --> 1

**Char. 103:** 1 --> 0

**Char. 146:** 0 --> 1

**Char. 223:** 0 --> 2

**Char. 244:** 0 --> 1

*Pholidosaurus*:

All trees:

**Char. 2:** 0 --> 1

**Char. 112:** 1 --> 0

*Sarcosuchus*:

All trees:

**Char. 3:** 2 --> 3

**Char. 74:** 0 --> 1

**Char. 91:** 1 --> 0

**Char. 260:** 0 --> 1

**Char. 334:** 0 --> 1

*Terminonaris* *browni*:

All trees:

**Char. 31:** 0 --> 1

**Char. 62:** 1 --> 0

**Char. 223:** 0 --> 2

**Char. 230:** 0 --> 1

**Char. 362:** 0 --> 1

**Char. 363:** 0 --> 1

*Rugosuchus* *nonganensis*:

All trees:

**Char. 33:** 0 --> 1

**Char. 118:** 1 --> 0

**Char. 244:** 0 --> 1

**Char. 262:** 0 --> 1

**Char. 338:** 0 --> 1

**Char. 363:** 0 --> 1

*Bayomesasuchus* *hernandezi*:

All trees:

**Char. 71:** 1 --> 2

*Itasuchus* *jesuinoi*:

All trees:

**Char. 1:** 2 --> 1

*Pepesuchus* *deiseae*:

All trees:

**Char. 20:** 0 --> 1

**Char. 33:** 1 --> 0

**Char. 72:** 2 --> 0

**Char. 97:** 1 --> 0

*Roxochampsa* *paulistanus*:

All trees:

**No autapomorphies.**

*Sahitisuchus* *fluminensis*:

All trees:

**Char. 21:** 1 --> 0

**Char. 51:** 0 --> 1

**Char. 53:** 1 --> 0

**Char. 54:** 0 --> 1

**Char. 56:** 1 --> 0

**Char. 69:** 0 --> 1

**Char. 71:** 1 --> 2

**Char. 116:** 0 --> 1

**Char. 117:** 1 --> 0

**Char. 126:** 0 --> 1

**Char. 182:** 0 --> 2

**Char. 185:** 0 --> 1

**Char. 197:** 0 --> 1

**Char. 199:** 1 --> 0

**Char. 221:** 1 --> 0

**Char. 253:** 0 --> 1

**Char. 276:** 1 --> 0

**Char. 332:** 0 --> 1

**Char. 333:** 0 --> 1

**Char. 335:** 2 --> 1

**Char. 356:** 1 --> 0

**Char. 381:** 0 --> 1

*Caririsuchus* *camposi*:

All trees:

**Char. 15:** 1 --> 0

**Char. 24:** 1 --> 0

**Char. 29:** 0 --> 1

**Char. 161:** 0 --> 2

**Char. 239:** 1 --> 0

**Char. 244:** 0 --> 1

*Barreirosuchus* *franciscoi*:

All trees:

**No autapomorphies.**

Crocodylomorpha:

All trees:

**No synapomorphies.**

Crocodyliformes:

All trees:

**Char. 1:** 0 --> 2

**Char. 17:** 0 --> 1

**Char. 22:** 0 --> 1

**Char. 41:** 0 --> 2

**Char. 43:** 0 --> 1

**Char. 46:** 0 --> 1

**Char. 49:** 0 --> 1

**Char. 61:** 0 --> 1

**Char. 62:** 0 --> 1

**Char. 75:** 0 --> 1

**Char. 86:** 0 --> 1

**Char. 112:** 1 --> 0

**Char. 185:** 0 --> 2

**Char. 191:** 0 --> 1

**Char. 192:** 0 --> 1

**Char. 239:** 0 --> 1

**Char. 309:** 0 --> 1

**Char. 334:** 1 --> 0

Some trees:

**Char. 14:** 0 --> 1

**Char. 28:** 0 --> 1

**Char. 35:** 0 --> 1

**Char. 48:** 0 --> 1

**Char. 56:** 0 --> 1

**Char. 80:** 0 --> 1

**Char. 90:** 0 --> 1

**Char. 100:** 1 --> 2

**Char. 133:** 1 --> 0

**Char. 318:** 1 --> 0

(*Hemiprotosuchus* + *Protosuchus*):

All trees:

**Char. 37:** 0 --> 1

**Char. 52:** 0 --> 1

**Char. 67:** 1 --> 2

**Char. 68:** 0 --> 1

Some trees:

**Char. 31:** 0 --> 1

**Char. 94:** 1 --> 0

(*Orthosuchus* ((*Gobiosuchus* + *Zaraasuchus*) ((*Zosuchus* + *Shichuanosuchus*) (*Hsisosuchus* + Mesoeucrocodylia))):

All trees:

**Char. 3:** 0 --> 1

**Char. 107:** 0 --> 1

**Char. 110:** 0 --> 1

**Char. 129:** 0 --> 1

**Char. 288:** 1 --> 0

**Char. 304:** 0 --> 1

**Char. 335:** 0 --> 1

((*Gobiosuchus* + *Zaraasuchus*) ((*Zosuchus* + *Shichuanosuchus*) (*Hsisosuchus* + Mesoeucrocodylia)):

All trees:

**Char. 33:** 0 --> 2

**Char. 45:** 0 --> 2

**Char. 78:** 0 --> 1

**Char. 339:** 0 --> 1

Some trees:

**Char. 133:** 0 --> 2

(*Gobiosuchus* + *Zaraasuchus*):

All trees:

**Char. 1:** 2 --> 1

**Char. 30:** 0 --> 1

**Char. 69:** 0 --> 1

**Char. 88:** 0 --> 1

**Char. 193:** 1 --> 0

**Char. 201:** 0 --> 1

**Char. 202:** 0 --> 1

**Char. 204:** 0 --> 1

**Char. 206:** 0 --> 1

**Char. 207:** 0 --> 1

**Char. 208:** 0 --> 1

**Char. 209:** 0 --> 1

**Char. 210:** 0 --> 1

**Char. 211:** 0 --> 1

((*Zosuchus* + *Shichuanosuchus*) (*Hsisosuchus* + Mesoeucrocodylia)):

All trees:

**Char. 23:** 0 --> 1

**Char. 34:** 0 --> 12

**Char. 36:** 0 --> 1

**Char. 38:** 0 --> 1

**Char. 73:** 0 --> 1

**Char. 115:** 0 --> 1

**Char. 140:** 0 --> 1

(*Zosuchus* + *Shichuanosuchus*):

All trees:

**Char. 189:** 0 --> 1

**Char. 198:** 0 --> 1

**Char. 336:** 0 --> 1

**Char. 388:** 0 --> 1

(*Hsisosuchus* + Mesoeucrocodylia):

All trees:

**Char. 15:** 0 --> 1

**Char. 49:** 1 --> 0

**Char. 67:** 1 --> 0

**Char. 71:** 0 --> 2

**Char. 109:** 0 --> 1

**Char. 112:** 0 --> 1

**Char. 133:** 2 --> 1

**Char. 138:** 1 --> 0

**Char. 158:** 0 --> 1

**Char. 190:** 1 --> 0

**Char. 221:** 0 --> 1

**Char. 352:** 0 --> 1

Mesoeucrocodylia:

All trees:

**Char. 17:** 1 --> 0

**Char. 183:** 0 --> 1

**Char. 187:** 0 --> 1

**Char. 195:** 1 --> 0

Neosuchia:

All trees:

**Char. 6:** 0 --> 2

**Char. 33:** 2 --> 0

**Char. 60:** 0 --> 1

**Char. 61:** 1 --> 2

**Char. 87:** 0 --> 1

**Char. 102:** 1 --> 0

**Char. 103:** 0 --> 1

**Char. 106:** 1 --> 0

**Char. 140:** 1 --> 0

**Char. 150:** 0 --> 1

**Char. 265:** 1 --> 0

**Char. 316:** 0 --> 1

**Char. 337:** 0 --> 1

(Pholidosauridae (*Hyposaurus* + Thalattosuchia)):

All trees:

**Char. 5:** 0 --> 1

**Char. 8:** 1 --> 0

**Char. 41:** 1 --> 0

**Char. 57:** 1 --> 0

**Char. 71:** 2 --> 3

**Char. 73:** 1 --> 0

**Char. 74:** 1 --> 0

**Char. 107:** 1 --> 0

Pholidosauridae:

All trees:

**Char. 33:** 0 --> 1

**Char. 40:** 0 --> 1

**Char. 159:** 0 --> 1

**Char. 214:** 0 --> 1

**Char. 225:** 0 --> 1

**Char. 228:** 0 --> 1

(*Terminonaris* + *Sarcosuchus*):

All trees:

**Char. 26:** 0 --> 1

**Char. 72:** 0 --> 2

**Char. 153:** 0 --> 1

**Char. 227:** 0 --> 1

**Char. 388:** 0 --> 1

(*Hyposaurus* + Thalattosuchia):

All trees:

**Char. 62:** 1 --> 0

**Char. 135:** 1 --> 0

**Char. 239:** 1 --> 0

Thalattosuchia:

All trees:

**Char. 28:** 1 --> 0

**Char. 42:** 1 --> 0

**Char. 45:** 2 --> 1

**Char. 50:** 1 --> 0

**Char. 54:** 0 --> 1

**Char. 61:** 2 --> 1

**Char. 131:** 0 --> 1

**Char. 185:** 0 --> 1

(*Steneosaurus* + *Pelagosaurus*):

All trees:

**Char. 6:** 2 --> 1

**Char. 13:** 1 --> 0

**Char. 19:** 1 --> 0

**Char. 388:** 0 --> 1

(*Metriorhynchus* (*Dakosaurus* + *Cricosaurus*)):

All trees:

**Char. 1:** 2 --> 0

**Char. 69:** 0 --> 1

**Char. 93:** 1 --> 0

**Char. 101:** 0 --> 2

**Char. 234:** 0 --> 1

**Char. 237:** 0 --> 1

**Char. 238:** 0 --> 1

(*Dakosaurus* + *Cricosaurus*):

All trees:

**Char. 60:** 1 --> 0

**Char. 63:** 1 --> 0

**Char. 241:** 0 --> 1

(Goniopholididae (*Theriosuchus* + Eusuchia)):

All trees:

**Char. 78:** 1 --> 2

**Char. 144:** 0 --> 1

**Char. 149:** 0 --> 3

**Char. 167:** 0 --> 1

Goniopholididae:

All trees:

**Char. 92:** 0 --> 1

**Char. 151:** 0 --> 1

**Char. 159:** 0 --> 1

**Char. 194:** 0 --> 1

**Char. 260:** 0 --> 1

**Char. 375:** 0 --> 1

(*Sunosuchus* (*Calsoyasuchus* + *Eutretauranosuchus*)) :

All trees:

**Char. 7:** 1 --> 0

**Char. 9:** 2 --> 1

(*Calsoyasuchus* + *Eutretauranosuchus*):

All trees:

**Char. 34:** 2 --> 1

**Char. 253:** 1 --> 0

(*Theriosuchus* + Eusuchia):

All trees:

**Char. 83:** 0 --> 1

**Char. 152:** 0 --> 1

**Char. 251:** 0 --> 1

Eusuchia:

All trees:

**Char. 40:** 0 --> 2

**Char. 63:** 1 --> 0

**Char. 84:** 0 --> 1

**Char. 96:** 1 --> 0

**Char. 138:** 0 --> 1

**Char. 247:** 0 --> 1

(*Boverisuchus* + *Hylaeochampsa*):

All trees:

**Char. 31:** 0 --> 1

((*Rugosuchus* + *Shamosuchus*) Crocodylia):

All trees:

**Char. 8:** 1 --> 0

**Char. 86:** 1 --> 2

**Char. 137:** 0 --> 1

**Char. 260:** 0 --> 1

**Char. 261:** 0 --> 1

**Char. 388:** 0 --> 1

(*Rugosuchus* + *Shamosuchus*):

All trees:

**Char. 20:** 0 --> 1

**Char. 40:** 2 --> 1

**Char. 53:** 1 --> 0

**Char. 69:** 0 --> 1

Crocodylia:

All trees:

**Char. 70:** 0 --> 1

**Char. 71:** 2 --> 0

**Char. 73:** 1 --> 2

**Char. 89:** 0 --> 1

**Char. 108:** 0 --> 1

(*Alligator* (*Crocodylus* + *Gavialis*)) :

All trees:

**Char. 58:** 0 --> 1

**Char. 91:** 1 --> 0

(*Crocodylus* + *Gavialis*):

All trees:

**Char. 8:** 0 --> 1

**Char. 65:** 1 --> 2

**Char. 116:** 1 --> 2

**Char. 159:** 0 --> 1

**Char. 245:** 0 --> 1

**Char. 260:** 1 --> 0

**Char. 261:** 1 --> 0

**Char. 388:** 1 --> 0

Ziphosuchia:

All trees:

**Char. 21:** 0 --> 1

**Char. 72:** 0 --> 2

**Char. 81:** 0 --> 1

**Char. 82:** 0 --> 4

**Char. 113:** 0 --> 1

**Char. 142:** 0 --> 1

**Char. 147:** 0 --> 1

**Char. 152:** 0 --> 1

**Char. 253:** 1 --> 0

**Char. 257:** 0 --> 1

**Char. 276:** 0 --> 1

**Char. 279:** 0 --> 1

**Char. 282:** 0 --> 1

**Char. 285:** 0 --> 1

**Char. 294:** 0 --> 1

**Char. 322:** 1 --> 2

**Char. 346:** 0 --> 1

**Char. 356:** 0 --> 1

Some trees:

**Char. 155:** 1 --> 0

Notosuchia:

All trees:

**Char. 8:** 1 --> 0

**Char. 74:** 1 --> 0

**Char. 125:** 0 --> 1

**Char. 128:** 0 --> 1

**Char. 154:** 0 --> 1

Some trees:

**Char. 106:** 1 --> 2

**Char. 161:** 0 --> 2

**Char. 213:** 0 --> 1

**Char. 307:** 0 --> 1

Uruguaysuchidae:

All trees:

**Char. 196:** 0 --> 1

**Char. 212:** 2 --> 1

**Char. 280:** 0 --> 1

Some trees:

**Char. 139:** 0 --> 1

(*Candidodon* + *Uruguaysuchus*):

All trees:

**Char. 110:** 1 --> 0

**Char. 175:** 0 --> 1

(*Araripesuchus* *gomesii* (*Anatosuchus* + *Araripesuchus* *wegeneri*)):

All trees:

**Char. 40:** 0 --> 1

**Char. 133:** 1 --> 2

**Char. 149:** 0 --> 1

**Char. 157:** 0 --> 2

**Char. 167:** 0 --> 1

**Char. 172:** 0 --> 1

**Char. 185:** 0 --> 1

**Char. 258:** 0 --> 1

Some trees:

**Char. 160:** 1 --> 0

**Char. 182:** 1 --> 0

(*Anatosuchus* + *Araripesuchus* *wegeneri*):

All trees:

**Char. 10:** 0 --> 1

**Char. 20:** 0 --> 1

**Char. 97:** 1 --> 0

**Char. 388:** 0 --> 1

((*Pakasuchus* + *Malawisuchus*) (*Simosuchus* (Baurusuchidae + advanced notosuchians))):

All trees:

**Char. 1:** 2 --> 1

**Char. 68:** 0 --> 1

**Char. 95:** 0 --> 1

**Char. 353:** 0 --> 1

Some trees:

**Char. 82:** 4 --> 1

**Char. 141:** 0 --> 1

(*Pakasuchus* + *Malawisuchus*):

All trees:

**Char. 112:** 1 --> 0

**Char. 182:** 1 --> 2

**Char. 184:** 0 --> 1

**Char. 328:** 0 --> 1

(*Simosuchus* (Baurusuchidae + advanced notosuchians)):

All trees:

**Char. 20:** 0 --> 1

**Char. 31:** 0 --> 1

**Char. 175:** 0 --> 1

**Char. 190:** 0 --> 1

**Char. 329:** 0 --> 1

(Baurusuchidae + advanced notosuchians):

All trees:

**Char. 39:** 0 --> 1

**Char. 61:** 1 --> 2

**Char. 71:** 2 --> 1

**Char. 110:** 1 --> 0

**Char. 161:** 2 --> 1

**Char. 215:** 0 --> 1

**Char. 219:** 0 --> 1

**Char. 222:** 0 --> 1

**Char. 291:** 0 --> 1

**Char. 295:** 1 --> 0

**Char. 303:** 0 --> 1

**Char. 334:** 0 --> 1

**Char. 338:** 0 --> 1

**Char. 354:** 1 --> 0

**Char. 382:** 0 --> 1

Baurusuchidae:

All trees:

**Char. 3:** 13 --> 0

**Char. 8:** 0 --> 1

**Char. 98:** 1 --> 0

**Char. 213:** 1 --> 2

**Char. 223:** 0 --> 1

(*Campinasuchus* (*Pissarrachampsa* (*Stratiotosuchus* + *Baurusuchus* *salgadoensis* + *Baurusuchus* *pachecoi* + *Baurusuchus* *albertoi*))):

All trees:

**Char. 74:** 0 --> 1

**Char. 375:** 0 --> 1

**Char. 385:** 0 --> 1

(*Pissarrachampsa* (*Stratiotosuchus* + *Baurusuchus* *salgadoensis* + *Baurusuchus* *pachecoi* + *Baurusuchus* *albertoi*)):

All trees:

**Char. 78:** 2 --> 0

**Char. 244:** 0 --> 1

Some trees:

**Char. 386:** 0 --> 1

(*Stratiotosuchus* + *Baurusuchus* *salgadoensis* + *Baurusuchus* *pachecoi* + *Baurusuchus* *albertoi*):

All trees:

**Char. 182:** 1 --> 0

**Char. 215:** 1 --> 2

**Char. 333:** 0 --> 1

**Char. 376:** 0 --> 2

**Char. 379:** 0 --> 1

Advanced notosuchians:

All trees:

**Char. 60:** 0 --> 1

**Char. 101:** 0 --> 1

**Char. 117:** 1 --> 0

**Char. 158:** 1 --> 0

**Char. 217:** 0 --> 1

**Char. 346:** 1 --> 2

(*Notosuchus* (*Mariliasuchus* + Sphagesauridae)):

All trees:

**Char. 73:** 1 --> 0

**Char. 110:** 0 --> 2

**Char. 127:** 0 --> 1

**Char. 182:** 1 --> 2

**Char. 265:** 1 --> 0

**Char. 342:** 0 --> 1

**Char. 363:** 0 --> 1

**Char. 369:** 0 --> 1

(*Mariliasuchus* + Sphagesauridae):

All trees:

**Char. 49:** 0 --> 1

**Char. 184:** 0 --> 1

**Char. 250:** 2 --> 0

**Char. 331:** 0 --> 1

**Char. 332:** 0 --> 1

**Char. 333:** 0 --> 1

**Char. 345:** 0 --> 1

**Char. 355:** 0 --> 1

**Char. 367:** 0 --> 1

Some trees:

**Char. 29:** 0 --> 1

**Char. 362:** 0 --> 1

Sphageosauridae:

All trees:

**Char. 138:** 0 --> 1

**Char. 275:** 0 --> 1

**Char. 343:** 0 --> 1

**Char. 359:** 1 --> 0

**Char. 361:** 0 --> 1

**Char. 368:** 0 --> 1

**Char. 372:** 0 --> 1

**Char. 386:** 0 --> 1

Some trees:

**Char. 213:** 1 --> 0

**Char. 360:** 0 --> 1

**Char. 365:** 0 --> 1

(*Adamantinasuchus* + *Yacarerani*):

All trees:

**Char. 363:** 1 --> 0

**Char. 366:** 0 --> 1

Some trees:

**Char. 218:** 0 --> 1

**Char. 249:** 0 --> 1

**Char. 362:** 1 --> 0

(*Caipirasuchus* *paulistanus* + *Caipirasuchus* *montealtensis*):

All trees:

**Char. 332:** 1 --> 0

**Char. 386:** 1 --> 0

Some trees:

**Char. 212:** 2 --> 1

(*Armadilhosuchus* + *Sphageosaurus* + *Caryonosuchus*):

All trees:

**Char. 97:** 1 --> 3

**Char. 133:** 1 --> 0

**Char. 154:** 1 --> 0

**Char. 336:** 1 --> 2

Some trees:

**Char. 114:** 0 --> 1

**Char. 135:** 0 --> 1

**Char. 358:** 1 --> 0

Sebecia:

All trees:

**Char. 40:** 0 --> 1

**Char. 108:** 0 --> 1

**Char. 146:** 0 --> 1

**Char. 148:** 0 --> 1

**Char. 149:** 0 --> 3

**Char. 151:** 0 --> 1

**Char. 157:** 0 --> 1

**Char. 167:** 0 --> 1

**Char. 260:** 0 --> 1

**Char. 264:** 0 --> 1

**Char. 267:** 0 --> 1

(*Stolokrosuchus* ((*Barreirosuchus* + *Ayllusuchus*) Itasuchidae)):

All trees:

**Char. 33:** 2 --> 1

**Char. 102:** 1 --> 0

**Char. 150:** 0 --> 1

**Char. 268:** 0 --> 1

**Char. 363:** 0 --> 1

**Char. 384:** 0 --> 1

((*Barreirosuchus* + *Ayllusuchus*) Itasuchidae):

All trees:

**Char. 93:** 1 --> 0

**Char. 133:** 1 --> 0

**Char. 241:** 0 --> 1

**Char. 335:** 2 --> 0

(*Barreirosuchus* + *Ayllusuchus*):

All trees:

**Char. 61:** 1 --> 2

Itasuchidae:

All trees:

**Char. 98:** 0 --> 1

**Char. 113:** 1 --> 0

**Char. 170:** 0 --> 1

**Char. 172:** 0 --> 1

(Peirosauridae (Mahajangasuchidae + Sebecidae)):

All trees:

**Char. 71:** 2 --> 1

**Char. 172:** 0 --> 1

**Char. 338:** 0 --> 1

Peirosauridae:

All trees:

**Char. 224:** 0 --> 1

**Char. 230:** 0 --> 2

**Char. 275:** 0 --> 1

**Char. 371:** 0 --> 1

((*Lomasuchus* + *Montealtosuchus*) (*Bayomenasuchus* (*Gasparinisuchus* +*Uberabasuchus*))):

All trees:

**Char. 10:** 0 --> 1

**Char. 200:** 0 --> 1

**Char. 201:** 0 --> 1

**Char. 388:** 0 --> 1

(*Lomasuchus* + *Montealtosuchus*):

All trees:

**Char. 31:** 0 --> 1

(*Bayomenasuchus* (*Gasparinisuchus* +*Uberabasuchus*)):

All trees:

**Char. 68:** 0 --> 1

(*Gasparinisuchus* +*Uberabasuchus*):

All trees:

**Char. 145:** 1 --> 0

(Mahajangasuchidae + Sebecidae):

All trees:

**Char. 1:** 2 --> 1

**Char. 161:** 0 --> 2

**Char. 168:** 0 --> 1

**Char. 336:** 0 --> 1

**Char. 374:** 0 --> 1

Mahajangasuchidae:

All trees:

**Char. 5:** 0 --> 1

**Char. 6:** 0 --> 2

**Char. 10:** 0 --> 1

**Char. 31:** 0 --> 1

**Char. 33:** 2 --> 4

**Char. 70:** 0 --> 1

**Char. 96:** 1 --> 2

**Char. 113:** 1 --> 0

**Char. 116:** 0 --> 2

**Char. 133:** 1 --> 2

**Char. 144:** 0 --> 2

**Char. 151:** 1 --> 0

**Char. 180:** 0 --> 1

**Char. 244:** 0 --> 1

**Char. 268:** 0 --> 1

**Char. 274:** 0 --> 1

**Char. 373:** 0 --> 1

**Char. 388:** 0 --> 1

Sebecidae:

All trees:

**Char. 129:** 1 --> 0

**Char. 132:** 0 --> 1

**Char. 163:** 0 --> 1

**Char. 206:** 0 --> 1

**Char. 264:** 1 --> 0

(Bretesuchus ((*Barinasuchus* (*Sebecus icaeirhinus* + *Sebecus huilensis*)) (*Lorosuchus* + *Sebecus* *querejazus* + *Iberosuchus* + *Bergisuchus* + *Pehuenchesuchus*))):

All trees:

**Char. 24:** 1 --> 0

**Char. 172:** 1 --> 0

**Char. 223:** 0 --> 1

**Char. 269:** 0 --> 1

**Char. 273:** 0 --> 1

**Char. 357:** 0 --> 1

Some trees:

**Char. 61:** 1 --> 2

((*Barinasuchus* (*Sebecus icaeirhinus* + *Sebecus huilensis*)) (*Lorosuchus* + *Sebecus* *querejazus* + *Iberosuchus* + *Bergisuchus* + *Pehuenchesuchus*))):

All trees:

**Char. 167:** 1 --> 0

**Char. 266:** 1 --> 0

(*Barinasuchus* (*Sebecus icaeirhinus* + *Sebecus huilensis*)):

All trees:

**Char. 39:** 0 --> 1

(*Sebecus icaeirhinus* + *Sebecus huilensis*):

All trees:

**Char. 74:** 1 --> 0

**Char. 130:** 1 --> 2

(*Lorosuchus* + *Sebecus* *querejazus* + *Iberosuchus* + *Bergisuchus* + *Pehuenchesuchus*):

All trees:

**Char. 145:** 1 --> 0

**Char. 148:** 1 --> 0

**Char. 160:** 0 --> 1

**Char. 265:** 0 --> 1
